# Efficient Integration of Ultra-low Power Techniques and Energy Harvesting in Self-Sufficient Devices: A Comprehensive Overview of Current Progress and Future Directions

**DOI:** 10.3390/s24144471

**Published:** 2024-07-10

**Authors:** Rocco Citroni, Fabio Mangini, Fabrizio Frezza

**Affiliations:** Department of Information Engineering, Electronics and Telecommunications, “Sapienza” University of Rome, 00184 Rome, Italy; rocco.citroni@uniroma1.it (R.C.); fabio.mangini@uniroma1.it (F.M.)

**Keywords:** Internet of Things (IoT), ultra-low-power design techniques (ULPDT), micro energy harvesting techniques (MEHT), power management unit (PMU), energy storage (ES), ultra-low power wireless communication protocol for IoT (ULP WCP)

## Abstract

Compact, energy-efficient, and autonomous wireless sensor nodes offer incredible versatility for various applications across different environments. Although these devices transmit and receive real-time data, efficient energy storage (ES) is crucial for their operation, especially in remote or hard-to-reach locations. Rechargeable batteries are commonly used, although they often have limited storage capacity. To address this, ultra-low-power design techniques (ULPDT) can be implemented to reduce energy consumption and prolong battery life. The Energy Harvesting Technique (EHT) enables perpetual operation in an eco-friendly manner, but may not fully replace batteries due to its intermittent nature and limited power generation. To ensure uninterrupted power supply, devices such as ES and power management unit (PMU) are needed. This review focuses on the importance of minimizing power consumption and maximizing energy efficiency to improve the autonomy and longevity of these sensor nodes. It examines current advancements, challenges, and future direction in ULPDT, ES, PMU, wireless communication protocols, and EHT to develop and implement robust and eco-friendly technology solutions for practical and long-lasting use in real-world scenarios.

## 1. Introduction

**Figure 1:**
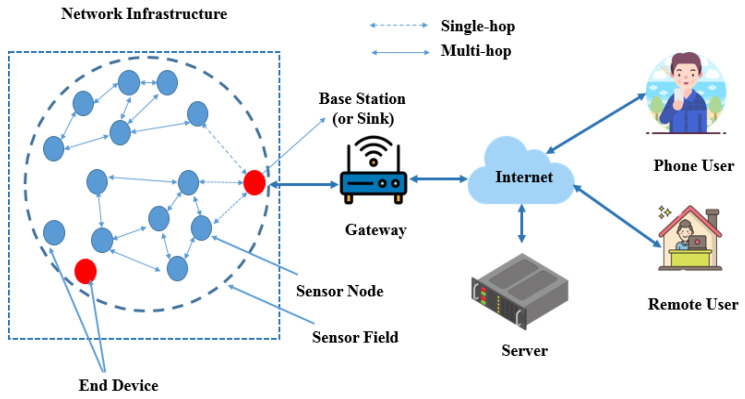
A typical WSN architecture.

The Internet of Things (IoT) is a network that links physical objects equipped with sensors, software, and wireless communication technologies to facilitate seamless and efficient communication. These wireless sensor nodes gather and transmit data within a wireless sensor network (WSN). A typical communication scenario for a WSN is reported in Figure 1. Communication within the network can be single-hop (transmitting data directly to a base station or sink for collection and processing, then sending them to a gateway and a remote server after) or multi-hop (the nodes relay data through neighboring nodes before reaching the base station). The multi-hop method expands network coverage and overcomes the range limitations of individual sensor nodes [1].

**Figure 2:**
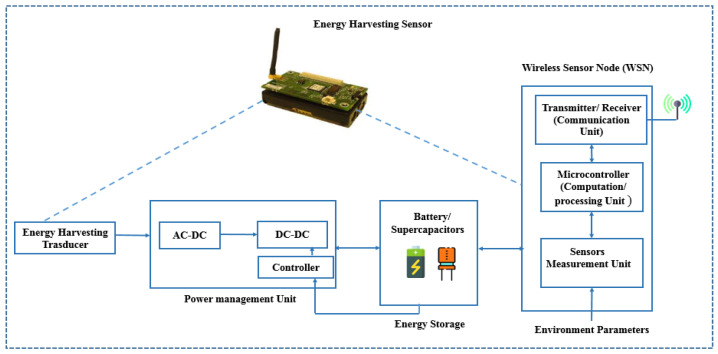
Generic IoT–wireless sensor node block diagram combined with energy harvesting.

Figure 2 shows a basic IoT–wireless sensor node structure, comprising four primary components: a sensing unit for data acquisition, a processing unit for local data processing, a wireless communication unit for data transmission, and an energy storage unit (typically a rechargeable battery/supercapacitor with limited energy capacity) for powering the sensor nodes. A power management unit (PMU) regulates and distributes power within each block of the sensor node, and an energy harvesting transducer converts ambient energy sources into electrical energy.

Today, batteries are the main energy source for sensor nodes, operating within a limited energy budget. Despite advancements in recent years, battery technology remains inefficient, with issues such as short lifespan, long recharge times, limited capacity, high-current pulses, and leakage. These factors influence performance and longevity. Recharging or replacing batteries in a hostile environment with numerous nodes is a significant challenge, requiring operational readiness for extended periods. ULPDT is utilized to optimize hardware, communication protocols, and data processing algorithms, reducing energy consumption and extending sensor node operation without frequent battery changes. Table 1 demonstrates that battery life in commercial sensor nodes is influenced by communication and processing subsystems. A continuous power supply is essential during active mode for data transmission and processing, while sufficient power is needed during inactive mode for passively sensing its environment.

**Figure 3:**
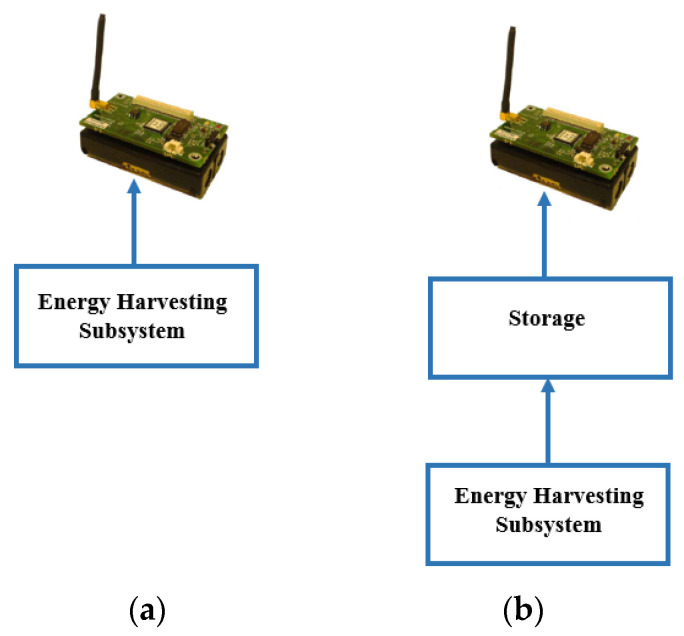
Typical architecture of an energy harvesting wireless sensor network (EHWSN). (**a**) EH without storage. (**b**) EH with storage.

ULPDT may not always be sufficient to extend battery life in all applications. Combining ULPDT with EHT can provide a more efficient solution. EHT involves capturing ambient energy and converting it into direct current (DC) using harvesters. This DC can power sensor nodes directly, eliminating the need for traditional batteries or be stored for later use. EH enables the use of sensor nodes in various environments and can extend network lifetime while reducing maintenance costs. In Figure 2, a sensor node equipped with EHT generates a new network called an energy harvesting wireless sensor network (EHWSN). Table 2 compares energy sources in the ambient, showing potential for efficient utilization within the 10 μW to 100 mW range, [8] aligning with the typical power requirements in Table 1. Despite benefits like inexhaustible sources and minimal environmental impact, EH poses challenges due to intermittent and unpredictable energy sources, as indicated in two situations [8,9]:

(a) If power consumption is lower than harvested power, EH can completely replace battery power. In this case, the sensor node may operate continuously and EH completely replaces the use of battery power, as illustrated in Figure 3a.
(b) If power consumption is higher, a buffer is needed to ensure continuous operation during times without power generation. EH can extend battery life and the enable perpetual operation of WSN, as illustrated in Figure 3b [8,9].

In the following sections, we will focus on techniques for energy saving combined with methods for producing power through energy harvesting, as well as effective strategies for managing and storing power efficiently.

### Organization of This Review

The paper is organized as follows: Section 2 explores existing techniques to minimize energy consumption in WSNs, including ultra-low-power design methodologies. Section 3 explores protocols for WSNs based on range, data rate, and specific use cases. Section 4 discusses power management techniques in WSNs and the relative challenges, as well as future directions. Section 5 reviews research on energy storage devices like rechargeable batteries and supercapacitors for continuous operation. Section 6 covers recent advances in harvesters based on electromagnetic fields, including working principles and materials. In addition, this paper highlights ongoing research challenges and provides insights into potential future directions for each harvester. Section 7 concludes the paper.

## 2. Investigation and Implementation of Ultra-Low-Power Design Technique (ULPDT) Applied for EHWSN

These techniques are particularly important in applications or scenarios where battery life or energy efficiency is critical. The trend towards smaller sensors leads to compact batteries being able to power them, but with limited energy capacity. ULPDT methods are used to reduce power consumption at different device levels (circuit design, system architecture, software algorithms, and power management), allowing you to extend the functionality of the device and reduce battery charging.

### 2.1. Operation States and Consumption Levels of a Typical Sensor Node

**Figure 4:**
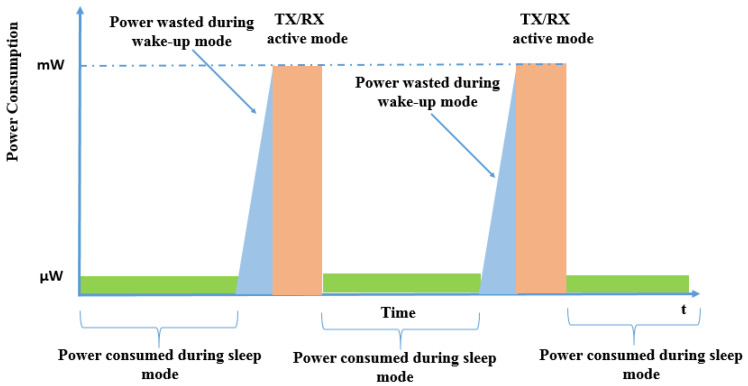
Different operational modes (sleep, wake-up and active mode) for a generic sensor node.

A sensor node in a network has three operational modes: sleep, wake-up, and active. Sleep mode conserves energy by minimizing power consumption and suspending functions, while the device does not transmit or receive data. Wake-up mode prepares the device to acquire or process data, acting as a transition state. Active mode is the most power-intensive, as the device actively transmits and receives data. Managing the transition between modes maximizes energy efficiency and extends the device’s lifespan, enabling effective performance in different environments. Figure 4 shows the operational modes for a generic sensor node. At a basic level, power consumption can be defined as the sum of the following factors [15]:

Total power consumed = Active mode power + Standby (sleep) mode power + Wake-up power

Sensing consumes less energy than communication in active mode, allowing the system to conserve energy during sleep mode for use in the more energy-demanding active mode (Table 3). ULPDT is mainly used to reduce energy consumption in communication subsystems during active operation, aiming to enhance efficiency and reduce overall power usage.

### 2.2. Identifying Sources of Power Dissipation in Circuits

This section offers a detailed explanation of ULPDT, with a specific emphasis on minimizing power usage within circuits. Power dissipation within circuits can be broken down into three main categories: dynamic, short-circuit, and static power dissipation, as outlined in Equation (1) [18,19,20,21,22,23,24,25,26,27,28,29,30,31,32,33,34,35,36,37,38,39,40,41,42,43].

(1) $$P=P_{Dynamic}+P_{SC}+P_{Static}$$

**Figure 5:**
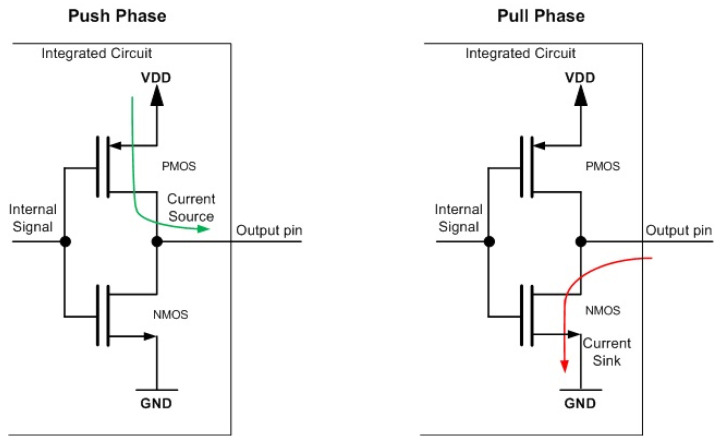
Dynamic power consumption for an inverter CMOS during the charging and discharging C. The green arrow indicates the charging process through the PMOS transistor. The red arrow indicates the discharge process through the NMOS transistor.

Dynamic power dissipation is the power consumed when a complementary metal oxide semiconductor (CMOS) circuit switches between logic states. In active mode, this phenomenon is modeled by Equation (2) and is evident during the charging and discharging of capacitive loads in the circuit (Figure 5) [18].

(2) $${\;P}_{switching}=\alpha C_{L\;}{V^{2}}_{DD}f_{SW}$$

where α is the switching activity of the gate, defined as the probability of the circuit node transitioning from 0 to 1, $C_{L}$ is the output load of the gate, $V_{DD}$ is the supply voltage, and $f_{SW}$ is the clock frequency.

**Figure 6:**
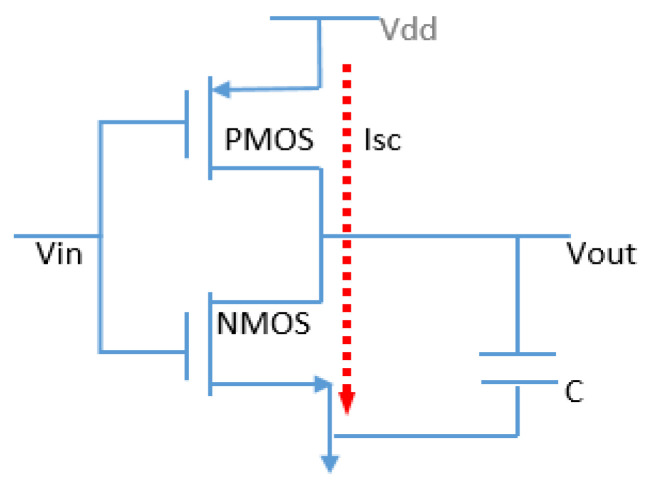
Short-circuit current path in an inverter CMOS during transients.

During time T, the load charges and discharges T·$f_{SW}$ times. Short-circuit dissipation occurs when both the positive-channel metal-oxide semiconductor (PMOS) and negative-channel metal-oxide semiconductor (NMOS) transistors conduct simultaneously, creating a temporary short circuit between $V_{dd}$ and the ground, highlighted in Figure 6 with red arrow.

**Figure 7:**
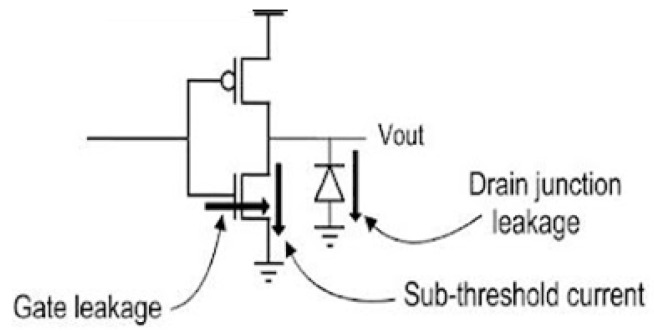
Leakage power components in an inverter CMOS (for $V_{GS}=0\;V$).

The phenomenon indicated in Figure 6 is modeled by the following Equation (3) [18,19,20,21,22,23,24,25,26,27,28,29,30,31,32,33,34,35,36,37,38,39,40,41,42,43]:

(3) $${\;P}_{sc}=t_{sc}V_{dd}I_{sc}f=C{V^{2}}_{dd}f$$

where $t_{sc}$ represents the time in which both devices are conducting, $I_{sc}$ is the direct-path current and $P_{sc}$ is the short-circuit power dissipation. Short-circuit dissipation is eliminated if NMOS and PMOS are never ON simultaneously, verifying the condition $V_{dd}<V_{THn}+\left|V_{THp}\right|$ (threshold voltage, commonly abbreviated as Vth for NMOS and PMOS, respectively). In CMOS technology, static power dissipation, represented as $P_{static}$, occurs when transistors are in a non-switching state, such as sleep mode. While the static power consumption in active mode is relatively low, it significantly increases in sleep mode. The main component of ${\;P}_{static}$ is leakage current. The equation for this phenomenon is [18,19,20,21,22,23,24,25,26,27,28,29,30,31,32,33,34,35,36,37,38,39,40,41,42,43]:

(4) $$P_{Static}=V_{DD}\cdot I_{Leakage}$$

where $I_{Leakage}$ epresents the total leakage current. This static power dissipation is primarily due to the three main currents reported in Figure 7. Subthreshold leakage current flowing through OFF transistors, gate leakage current across the gate dielectric, and junction leakage current through a reverse-biased semiconductor junction all play a significant role in static power consumption. Equation (4) can be written as [18,19,20,21,22,23,24,25,26,27,28,29,30,31,32,33,34,35,36,37,38,39,40,41,42,43]:

(5) $$P_{leak}≅\;P_{sub}+P_{gate}+P_{junc}=V_{DD}I_{sub}+V_{DD}I_{gate}+\left|V_{bs}\right|I_{j}$$

where $P_{sub}$, $P_{gate}$ and $P_{junc}$ represent the subthreshold, gate, and junction leakage power consumption, respectively. Similarly,$\;I_{sub}$, $I_{gate}$, and $I_{j}$ represent the subthreshold, gate, and junction leakage currents. $V_{DD}$ and $V_{bs}$ denote the supply and body bias voltages, respectively. Subthreshold leakage current in CMOS occurs when transistors operate in the subthreshold region, with gate-source voltage below the threshold needed to turn on the transistor ($V_{GS}<V_{TH}$).

Gate leakage current in CMOS occurs when electric current flows through the gate insulator of a transistor, even when the gate-source voltage is zero. This leakage current becomes more significant as transistors shrink and gate insulators become thinner. It is primarily caused by two mechanisms: Direct Tunneling (DT) and Fowler–Nordheim Tunneling (FNT) [44]. Direct tunneling is critical at lower voltages and with thin oxides, while Fowler–Nordheim tunneling is prominent at high voltages and with a moderate oxide thickness. Junction leakage current in CMOS is a small amount of current that flows between the source and drain terminals of a transistor, even when it is turned off. This type of leakage current occurs when the source and drain junctions of the transistor are reverse-biased.

#### Dynamic Power Reduction Approach

Dynamic power reduction utilizes techniques such as dynamic voltage scaling (DVS), dynamic frequency scaling (DFS), minimized switched capacitance, and reduced α, to decrease the power usage of CMOS transistors without compromising performance. Table 4 lists the pros and drawbacks of each technique for effective power reduction [18,19,20,21,22,23,24,25,26,27,28,29,30,31,32,33,34,35,36,37,38,39,40,41,42,43].

### 2.3. Static Power Reduction Techniques

A strategic method called static power reduction is used to decrease the power consumption in CMOS transistors within the steady state (all ON or all OFF). This method integrates various techniques into a CMOS circuit to reduce leakage current, some of which are reported as follows. Clock gating is a technique that involves selectively disabling the clock signal to unused or idle registers and clock trees within the design.

This helps to reduce power consumption and improves the overall efficiency in digital circuits. Power gating is a technique that selectively shuts off power to inactive or idle blocks in the design, effectively reducing both static and dynamic power consumption by eliminating leakage current in these areas. Voltage scaling is a method that involves reducing the operating voltage of a design in order to decrease power consumption. While this can result in an energy-efficient system, it may also influence performance. Therefore, it is essential to closely monitor and manage the voltage scaling to ensure that the design still meets its performance requirements. Sleep modes involve putting the design into a low-power state when it is idle or not in use. This can help reduce static power consumption by disabling or reducing power to certain blocks or components of the design. Using multiple threshold voltages for different parts of the design can help reduce static power consumption by optimizing voltage levels for different power modes or operating conditions. In Reverse Body Biasing (RBB), a voltage is applied to the body terminal of the transistor to change its threshold voltage. By adjusting this voltage, the transistor can operate at different speeds or power consumption levels. Duty cycling is a technique used to save power by periodically turning on and off a transmitter. Multiple sources of voltage ($V_{DD}$) is used to power different functional blocks at different voltages, optimizing performance and power consumption. This technique is useful in eliminating both static and dynamic power dissipation. Transistor stacking involves vertically stacking multiple transistors within a single semiconductor device to increase functionality and performance while minimizing size. It also improves power consumption, heat dissipation, and overall performance by reducing parasitic capacitance and resistance [18,19,20,21,22,23,24,25,26,27,28,29,30,31,32,33,34,35,36,37,38,39,40,41,42,43].

### 2.4. Software and System-Level Optimizations

Software and system-level optimizations refer to the various techniques and strategies used to improve the performance, efficiency, and overall functionality of software programs and systems. These optimizations can involve rewriting code, configuring settings, utilizing specific algorithms, and implementing best practices to ensure the software or system runs smoothly and efficiently.

### 2.5. Logic and Architecture-Level Optimizations

Optimizing logic and architecture at the logic level is crucial given the increasing complexity of modern digital devices. During optimization, the focus shifts to functionality and gate sizing within fixed technology parameters. Constrained power optimization aims to reduce power consumption without influencing the critical path length. Techniques like “path equalization” align signal paths in logic networks to minimize spurious switching activity. Adjusting gate sizes on fast paths can optimize power consumption by balancing propagation delays. Additional strategies at the gate level include re-factoring, remapping, and pin swapping. At the architecture level, techniques such as clock gating help conserve power by stopping inactive units, though this may potentially affect overall system performance [18,19,20,21,22,23,24,25,26,27,28,29,30,31,32,33,34,35,36,37,38,39,40,41,42,43].

## 3. Exploring Efficient Wireless Protocols for Low Power Connectivity: A Comparative Analysis

Energy-saving protocols have been created to improve sensor node functionality and optimize performance across different distances. We compare energy-efficient wireless protocols for both terrestrial and non-terrestrial connectivity, focusing on key parameters for each type. Our goal is to help designers choose the best protocol to maximize battery life in various applications.

### 3.1. Energy Saving Protocol (ESP) for EHWSN

**Figure 8:**
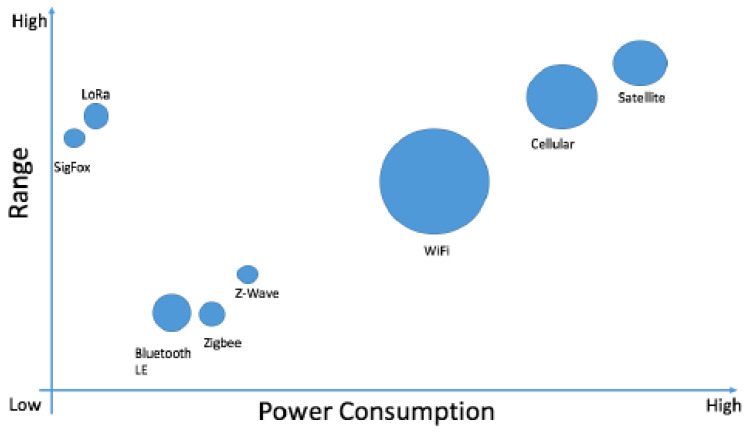
Power consumption vs. range for common communication protocols.

Reliable and secure connectivity among sensor nodes is imperative in network protocols for streamlined data transfer and effective communication. Energy-efficient protocols play a crucial role in extending the network’s lifespan by reducing the need for frequent battery replacement. This section delves into some promising energy-efficient protocols for sensor nodes and their effects on network efficiency and scalability. Key parameters for evaluating protocols include power consumption, distance coverage, data coding efficiency, complexity, and rates. Figure 8 illustrates terrestrial and non-terrestrial connectivity in terms of power consumption versus range, with bubble size representing data throughput.

Integrating Terrestrial and Non-Terrestrial Protocols (ITA NTP) offers seamless connectivity utilizing a variety of protocols, including short- and long-range wireless communication, advanced cellular technologies (2G, 3G, 4G, 5G, and future 6G), and satellite communication. More information on each type is available in the following sections. The article outlines eight different types of short-range wireless communication technology [45].

#### 3.1.1. Bluetooth

Bluetooth technology allows for wireless communication between sensor nodes over short distances. It has continued to evolve through various generations, from version 2.0 to the latest 5.3, incorporating features such as low energy (LE) with the introduction of version 4.0. These protocols utilize radio waves to transmit data within a range of approximately 10 m for most devices. However, the range can vary depending on the specific Bluetooth version and the surrounding environment. The latest Bluetooth version, Bluetooth 5, has an extended range of up to 240 m under ideal conditions. One notable aspect of this technology is the secure connection it establishes between devices, guaranteeing data protection through encryption. Key technical parameters for various versions of Bluetooth and Bluetooth Low Energy (BLE) can be found in Table 5 [46,47,48,49,50].

#### 3.1.2. Ultra-Wideband (UWB)

UWB technology utilizes minimal energy to transmit large amounts of data over short distances. It functions in the frequency range of 3.1~10.6 GHz, reducing interference and facilitating high-bandwidth communication. UWB splits its frequency band into numerous wide channels, allowing for data rates ranging from 53 Mbits/s to 480 Mbits/s. In contrast to conventional spread spectrum technologies, UWB’s transmission mode does not disrupt narrowband or carrier transmissions in the same frequency band [46,47,48,49,50].

#### 3.1.3. Wi-Fi (Wireless Fidelity)

Wi-Fi represents a popular wireless communication technology based on the IEEE 802.11 series standard. It is commonly used for local area networks (LANs) with a standard range of up to 100 m and high throughput, making it interoperable with nearby base stations. Wi-Fi has evolved through various generations, such as 802.11b, 802.11a, 802.11g, 802.11n, 802.11ac, 802.11ah, 802.11ax, and the latest, 802.11be. The technical parameters for different versions of Wi-Fi can be found in Table 6 [46,47,48,49,50].

#### 3.1.4. ZigBee

ZigBee is a low-power, low-data-rate wireless communication protocol operating on the IEEE 802.15.4 standard. It is used for small-scale networks, organized in a star or mesh topology. The protocol enables one central coordinator device to manage communication with multiple sensor nodes up to 10–20 m within the 2.4 GHz band. The technical parameters can be found in Table 7 [46,47,48,49,50].

#### 3.1.5. Z-Wave

Z-Wave, commonly used for sensor nodes, represents a wireless communications protocol that operates on a low-power, low-frequency radio band. It utilizes mesh networking technology, where every device serves as a “node” that communicates with other devices within the network. Each Z-Wave network can support up to 232 devices, with a maximum range of 100–300 m for point-to-point communication, ideal for short messages. The technical parameters can be found in Table 8 [46,47,48,49,50].

#### 3.1.6. IPv6 over Low-Power Wireless Personal Area Networks (6LoWPAN)

IPv6 over Low-Power Wireless Personal Area Networks (6LoWPAN) is an innovative technology that allows for the utilization of IPv6 on low-power, low-bandwidth wireless personal area networks. Specifically designed to function on a variety of low-power wireless technologies, such as IEEE 802.15.4 and BLE, 6LoWPAN can enable communication over distances of up to 100 m. A typical 6LoWPAN device may have an average current consumption of a few tens of μA during normal operation and a sleep current consumption of a few nA when the device is in a low-power sleep mode. The technical parameters are reported in Table 9 [51,52,53,54,55].

Long-range wireless communication technology is essential in modern society, allowing for communication over vast distances without the need for physical infrastructure. Five types of this technology include [51,52,53,54,55].

#### 3.1.7. LoRaWAN (Long-Range Low-Power Wide Area Network)

LoRaWAN is a low-power, wide-area networking protocol designed for the long-range communication of small amounts of data. It uses a chirp spread spectrum (CSS) modulation technique to achieve long-range communication while consuming minimal power. This allows battery-powered sensor nodes to operate for years without needing replacement. LoRaWAN operates in a star topology with bidirectional communication between end nodes and gateways—primarily at 900 MHz, 868 MHz, and 400 MHz, depending on country regulations. It has a line-of-sight range of up to 100 km with two-way communications, and up to 20 km in typical non-line-of-sight applications. The technical parameters can be found in Table 10 [51,52,53,54,55].

#### 3.1.8. SigFox

SigFox is a global network, which represents an energy-efficient alternative to traditional cellular networks for sending small amounts of data over long distances. The network is highly scalable and can support millions of connected devices with minimal infrastructure. The technical parameters can be found in Table 11 [51,52,53,54,55].

#### 3.1.9. Narrowband Internet of Things (NB-IoT)

NB-IoT is a low-power wide area network (LPWAN) technology that enables sensor nodes to communicate over long distances with minimal interference. It operates on licensed cellular networks and uses a narrowband frequency to transmit small amounts of data efficiently. This helps in conserving battery life. The technical parameters can be found in Table 12 [51,52,53,54,55].

#### 3.1.10. 2G, 3G, 4G LTE, and 5G Networks

The evolution of mobile networks from 2G to 5G has sparked a revolution in the way we communicate and stay connected. With each generation, there have been significant improvements in speed, coverage, and the number of connections possible. From the basic voice services of 2G to the high-definition video streaming capabilities of 4G and 4G LTE, mobile networks have continuously enhanced the way we interact with technology and each other (see Table 13). The latest 5G technology boasts ultra-fast data speeds, minimal latency, and extensive connectivity for the Internet of Things (IoT), leading to groundbreaking advancements in industries such as healthcare and smart cities [54,55].

Each data transmission protocol has its own set of advantages and disadvantages, making them suitable for different purposes and applications. It is important to consider the specific requirements and constraints of a network or system when choosing the appropriate protocol for data transmission. The analysis in Table 14 shows the progression from 2G to 5G cellular network technology, with 5G representing a major step forward [56].

6G networks promise faster speeds, lower latency, and more reliable connections compared to 5G. They are expected to support data rates up to 1 Tbit/s and enable new applications. The technical parameters are indicated in Table 15 [57].

#### 3.1.11. Satellite Communication and Integration of Low Earth Orbit (LEO) Satellite with 5G

Satellite communication is a versatile method of transmitting data, voice, and video signals through artificial objects placed into orbit around the Earth. These artificial objects, known as satellites, are equipped with transponders that receive and amplify signals from ground stations before retransmitting them to another ground station. The two main types of communication satellites are geostationary satellites (GEO) and Low Earth Orbit (LEO) satellites. GEO satellites remain fixed above a specific point on the Earth’s surface, approximately 35,786 km above it, while LEO satellites orbit closer to the Earth, approximately 2000 km, providing faster communication and lower latency. The technical parameters are reported in Table 16, highlighting the advantages and disadvantages of GEO and LEO satellites, respectively. One of the most significant developments in technology today is the merging of LEO satellites with 5G technology, which is revolutionizing internet connectivity by delivering high-speed, low-latency services to remote and underserved regions [58].

## 4. Optimizing Energy Efficiency: Key Concepts for Power Management Unit

To ensure the optimal functionality of sensor nodes, it is essential to integrate an energy harvester. This integration will create a seamless system that allows for continuous operation, ultimately maximizing efficiency and sustainability. It is important to keep in mind that the energy harvested may be intermittent and unpredictable. Therefore, it is crucial to guarantee that the harvested energy exceeds the system’s requirements, even in the face of constraints such as limited potential and low conversion efficiency. For this reason, a power management unit (PMU) is necessary for efficiently regulating power from variable sources and adapting to varying loads.

### 4.1. Mechanisms Proposed for Managing the Dynamics of EH: Energy-Neutral, Power-Neutral, and Intermittent Computing

In WSN applications, energy consumption during sensing is often underestimated compared to communication. Studies have shown that the power consumed during sleep mode can be significant, even with a very low duty cycle (less than 0.1%). Sleep mode may not always be the most efficient choice, as certain components still draw power for extended periods. On the other hand, active mode requires more power for data processing but for shorter durations. The efficient management of static and dynamic power is crucial for maximizing battery life. Various mechanisms, including energy-neutral, power-neutral, and intermittent computing, have been proposed in the literature to address the fluctuating levels of energy availability. Energy-neutral operation (ENO) involves consuming energy only when there is surplus energy from the harvesting source, aiming to maintain a balance between consumption and generation to maximize battery life. Power-neutral operation (PNO) focuses on matching power consumption with power generation to prevent imbalances that can affect system stability. However, in areas with limited resources, ENO and PNO may not be suitable, which can lead to disruptions and shutdowns. Intermittent computing (IC) is a power-saving technique that preserves system state during low energy levels, minimizing power consumption without interrupting regular operation [59,60,61,62]. The ultra-low power management unit (ULPMU) plays a key role in operating efficiently near the ENO state to maintain a balance between consumption and generation.

### 4.2. Maximizing Energy Efficiency: General Concept for PMU

**Figure 9:**
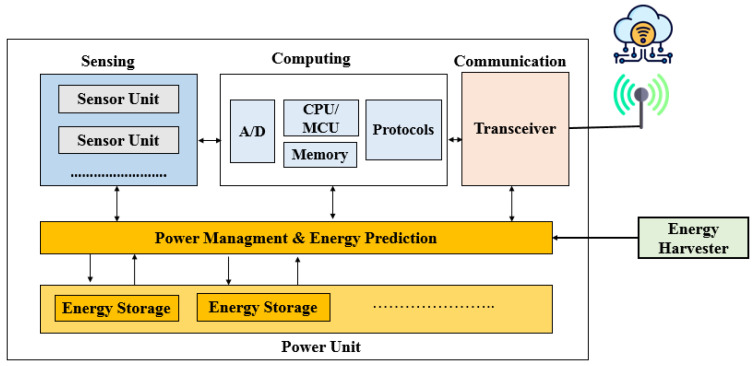
Generic wireless sensor node combined with EH.

A PMU helps regulate power levels from a harvester to maintain a consistent supply to a sensor node during operational status changes (sleep, wake-up, active mode, or shutdown). It balances power generation, load requirements, and operational status to extend the node’s lifetime. Figure 9 shows a generic wireless sensor node combined with EH. The PMU block is engineered to deliver consistent power input to the battery, ensuring stability and reliable performance, but this also includes controlling the power supply to the sensor, processor, transceiver, protocols, and memory, to ensure the optimal use of power and maximize the node’s battery life. In the context of communication protocols, the PMU and communication protocol must work together to optimize power usage during data transmission and reception, and coverage optimization strategies. Table 17 [1,8] shows some of the specific functions of a PMU within a wireless sensor node.

**Figure 10:**
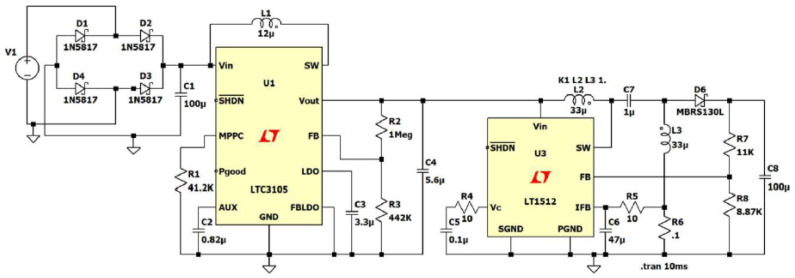
Electric circuit of a commercial ultra-low power management unit (ULPMU).

Figure 10 indicates a commercial ultra-low power management unit (ULPMU), simulated using LTspice XVII. This circuit includes an energy source, a full-wave bridge rectifier circuit, a DC-DC converter circuit, and a battery charging circuit, indicated with C8.

A generic harvester such as Radio Frequency (RF) or Infrared (IR) on the left side of Figure 10 generates alternating current (AC) output voltage, which needs to be converted to DC using an AC-DC converter stage. Energy harvesters typically have output voltages below 1 V. In this scenario, it is essential to use a rectenna array to simulate an external power source, V1, due to the insufficient output power and low voltage levels of a single rectenna. Typically, the single rectenna provides a few fW and μV. The Schottky diodes (D1, D2, D3, D4) are commonly used in rectifying bridge circuits due to their low forward voltage drop of 0.3~0.4 V. In addition, Schottky diodes are known for their faster recovery time and superior switching speed, making them the perfect choice for high-speed switching applications. Solar cells, thermoelectric generators, and pyroelectric generators produce a fluctuating DC output voltage. To efficiently utilize this energy source, a DC-DC converter, with an adjustable conversion factor and a controller, is necessary to maintain the appropriate bias for the storage. The energy collected by the harvester is transmitted to the storage unit through PMU, which is regulated by a microcontroller inside LTC3105 and LT1512. The microcontroller is equipped with various components, such as an analog-to-digital converter (ADC), a general-purpose input and output device (GPIO), and a digital-to-analog converter (DAC). The primary function of a DC-DC converter is to maintain a consistent output voltage that aligns with the requirements of the energy storage input (capacitor, supercapacitor, or rechargeable battery). Ultimately, among the wide array of DC-DC converters discussed in the literature, the LTC3105 and LTC3018 step-up converters emerge as the most efficient choices [8,16,62,63].

These converters have demonstrated exceptional performance in ensuring a stable output voltage even in the face of input voltage fluctuations. Particularly noteworthy is the LTC3018 converter’s capability to function with inputs as low as 20 mV. A detailed comparison of these two leading commercial DC-DC converters can be found in Table 18. To ensure the continuous operation of the wireless sensor node during high peak electricity demands, a storage element (capacitor C8) should be designed to effectively store the excess power accumulated over time [64,65,66].

## 5. Maximizing Sustainability and Reliability with Efficient Energy Storage Solutions

Energy Harvesting (EH) is indicated as a possible alternative to traditional battery-powered devices, offering numerous advantages described in the specific section. However, EH also exhibits a notable limitation due to the unpredictable and sporadic nature of energy sources, which makes continuous energy generation unsuitable for sensor nodes. Table 19 lists energy source types with detailed definitions [16].

Table 20 provides an overview of various energy sources and their characteristics investigated in this review [1].

A sensor node must be designed to operate indefinitely by consuming power within the limits of the harvester’s capabilities, as reported in Equation (6) [65,66].

(6) $$E\left(i,T\right)=\int_{t_{0}}^{t}[P_{s}\left(i,t\right)-P_{c}\left(i,t\right)]dt$$

In Equation (6), *E* represents energy generated by the harvester, $P_{s}\left(i,t\right)\;and\;P_{c}\left(i,t\right)$ represent power quantities. $P_{s}\left(i,t\right),$ represents the power generated by energy harvester, whereas $P_{c}\left(i,t\right)$ represents the power consumed by a generic sensor node i during the interval of (*t*, *t*_0_). Therefore, the designer’s goal is to ensure that a sensor node equipped with a non-ideal energy storage device works near ENO. This condition is verified by applying Equation (7) [65,66].

(7) $$B_{0}+ƞ\int_{0}^{t}[P_{s}\left(t\right)-P_{c}\left(t\right)]dt-\int_{0}^{t}[P_{c}\left(t\right)-P_{s}\left(t\right)]dt-\int_{0}^{t}P_{leak}dt\geq B\;\;\;\;\;\;\forall \;t\epsilon [0,\infty )$$

In Equation (7), $B_{0}$ represents the initial energy stored in the ideal energy buffer, *B* represents the energy buffer, η represents the charging efficiency of the storage component, which is always less than one, and $P_{leak}$ represents the energy lost associated with leakage.

### 5.1. Battery Options: Non-Rechargeable vs. Rechargeable

**Figure 11:**
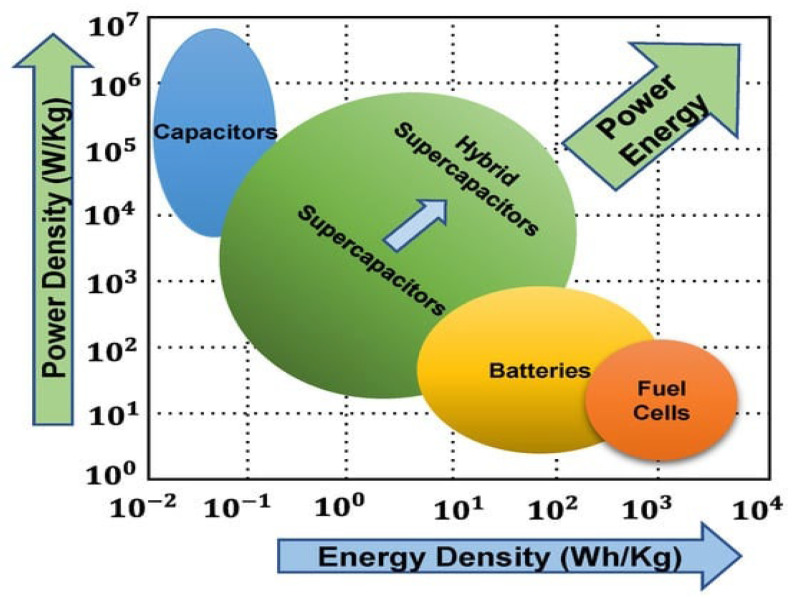
The Ragone plots for various energy storage devices. Reprinted with permission from ref. [68].

Energy storage (ES) is critical for efficient energy management, storing excess energy during low demand and releasing it during high demand. This section examines different ES technologies, applications, benefits, challenges, and their impact on the future of energy, as reported in Figure 11 [67].

Batteries and fuel cells are both devices that produce electricity through chemical reactions, although they function on distinct operating principles. A battery stores and releases electrical energy through a chemical reaction with an electrolyte, using electrodes and a separator to prevent direct contact. During charging, electrons are taken by the anode and released by the cathode, creating a voltage difference. The separator acts as a barrier to prevent short circuits, while still allowing ions to flow freely for chemical reactions to occur. On the contrary, a fuel cell functions by transforming chemical energy from a fuel into electricity through an ongoing chemical reaction with an oxidizing agent. Batteries are categorized into primary (non-rechargeable) and secondary (rechargeable) types. Non-rechargeable batteries, such as alkaline and zinc-carbon, need to be replaced when they are depleted. Both battery types are ideal for low-power devices, such as microsensors, that have a consumption of less than 50 μW [8]. Rechargeable batteries have superior energy density and can be used repeatedly, but their lifespan is limited by factors like temperature and charge cycles [1,60,62,68,69]. Table 21 reports the key parameters associated with rechargeable and non-rechargeable Batteries [10].

Lead-acid batteries (rechargeable) generate electrical energy through a chemical reaction between lead and sulfuric acid. They are durable and affordable, but have a lower energy density than lithium-ion batteries. They require frequent maintenance and emit harmful gases.

**Figure 12:**
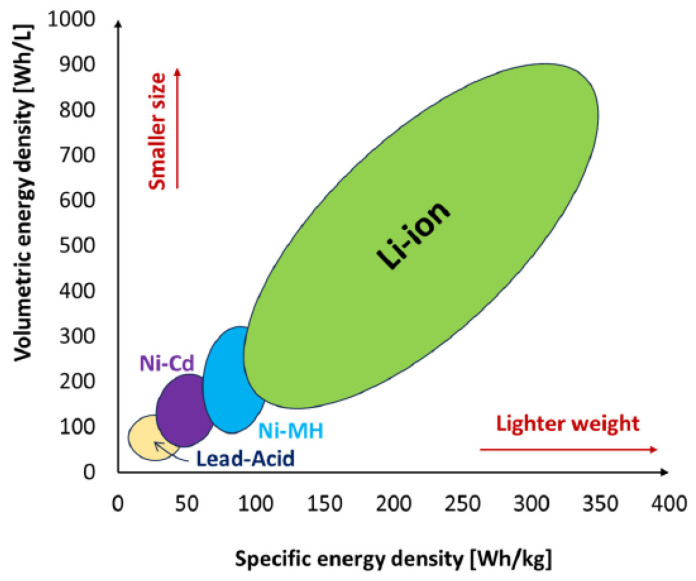
Some current commercial batteries are selected considering parameters such as specific energy density and volumetric energy density. Reprinted with permission from ref. [69].

The non-rechargeable manganese dioxide lithium (MnO_2_Li) battery uses manganese dioxide (MnO_2_) as the cathode material. This type of battery offers high energy density and stability, but the voltage decreases over time. The rechargeable Li-polymer battery features a polymer electrolyte, making it safe and flexible. However, it has a limited voltage range and is sensitive to heat. The non-rechargeable lithium thionyl chloride (LiSOCl_2_) battery with high capacity, low self-discharge rates, and a voltage of 3.6 V, is indicated for extreme temperatures. It offers outstanding energy density and longevity but must be disposed of properly. The lithium sulfide (LiO_2_S) battery represents a next-gen rechargeable battery, with high energy density and eco-friendly components. However, this type of battery has a limited lifespan, potential for overheating, and high manufacturing costs. Nickel-Cadmium (NiCd) battery is efficient and rechargeable, with a long cycle life. However, this type of battery has low energy density, memory effect, and cadmium toxicity, being replaced by lithium-ion batteries. Nickel-metal hydride (NiMH) batteries are rechargeable with high energy density, long cycle life, and low self-discharge rate. The rechargeable lithium-ion (Li-Ion) battery is known for its high energy density, long lifespan, and lightweight design, making it a popular choice for portable electronics. MnO_2_ batteries, also known as manganese dioxide batteries, are a type of primary (non-rechargeable) battery that utilizes manganese dioxide as the cathode material. The MnO_2_ battery typically has a longer shelf life and higher energy density compared to other types of primary batteries. It is also known for its stable voltage output and relatively low cost [70]. Figure 12 indicates some of the current commercial batteries reported in Table 21.

Table 22 reports an overview of the latest battery generations, highlighting their key features and specifications for convenient comparison, as well as the recent advancements [70,71].

### 5.2. Maximizing Efficiency: The Role of Capacitors and Supercapacitors in Energy Storage

#### 5.2.1. Capacitors

**Figure 13:**
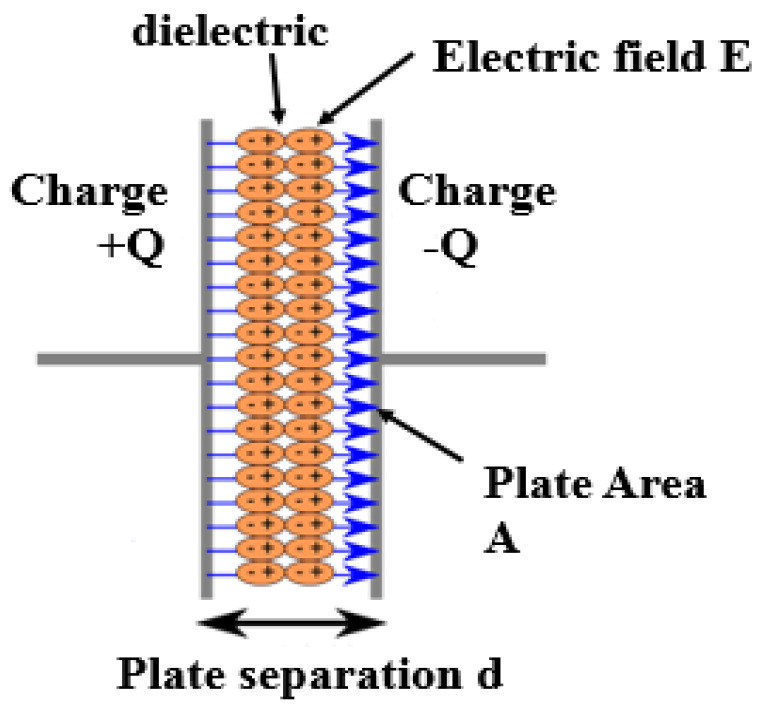
Basic structure and working principle of capacitor.

Although batteries and capacitors are both energy storage devices, they differ in construction, characteristics, and applications. Capacitors, indicated in Figure 13, function by storing electrical energy through the establishment of an electric field E between two plates separated by a dielectric material. When a voltage source is connected to a capacitor, current flows into the capacitor to charge it. The capacitor stores electrical energy as potential energy, with the amount directly proportional to the voltage and capacitance. When connected to a load, the capacitor discharges its energy, similar to a small rechargeable battery. For EH application, the energy is rectified and increased in voltage via boost converter DC-DC before being stored for later use. Table 23 [72] reports a comparison between batteries and capacitors based on temperature sensitivity, charging/discharging speed, and voltage output. These data are essential for determining the compatibility and performance of these energy storage devices in various applications. The information provided in Table 24 indicates that batteries are most appropriate for situations that require a continuous and reliable power source, while capacitors are better suited for applications where quick energy bursts and rapid energy transfer are necessary. For instance, capacitors are ideal for transmitting periodic data during active modes, such as in TX/RX burst transmission [72].

#### 5.2.2. Supercapacitors

**Figure 14:**
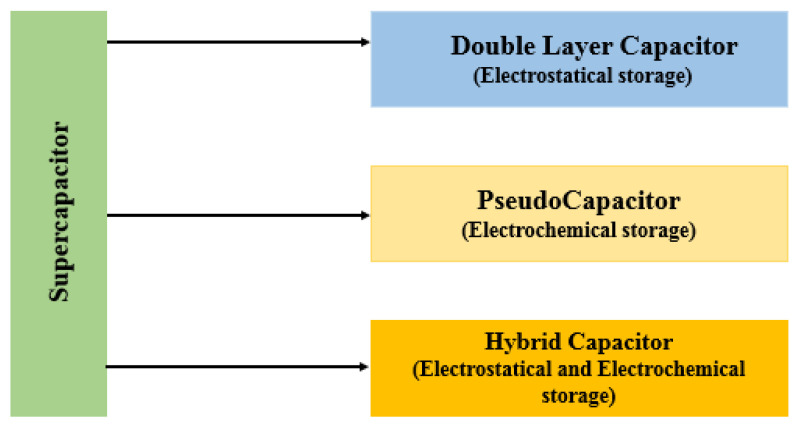
Tree of supercapacitor types.

**Figure 15:**
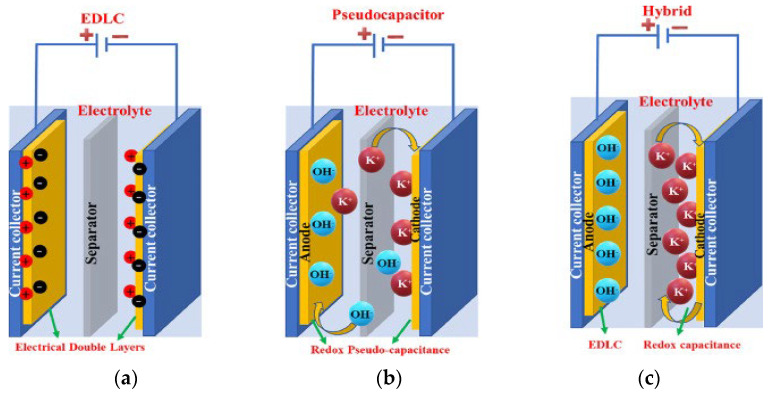
Schematic structure of supercapacitor types; (**a**) EDLC; (**b**) PC; (**c**) hybrid.

Supercapacitors, also known as ultra-capacitors, are advanced types of capacitors that have a higher energy storage capacity compared to traditional capacitors. This is achieved using both electrostatic and electrochemical processes, making them a powerful and efficient energy storage solution. As in Figure 14, they can be divided into electric double-layer capacitors (EDLCs), pseudo capacitors (PCs), and hybrid supercapacitors (HSCs). Supercapacitors serve as a crucial intermediary between batteries and capacitors, making them ideal for applications that require the rapid delivery of power for short durations, typically ranging from seconds to minutes. Rechargeable batteries, on the other hand, excel at storing larger amounts of energy over longer periods. In Figure 15a, an EDLC stores energy through the separation of charge at the interface between a thinner electrolyte dielectric and a high-surface-area electrode. This double-layer formation allows for high capacitance and fast charging/discharging rates. In Figure 15b, PC, on the other hand, relies on reversible redox reactions at the electrode–electrolyte interface to store energy. This mechanism allows for a higher energy density than EDLCs, however, this comes at a sacrifice in speed. In Figure 15c, HSC combines the best of both worlds, utilizing both EDCL and PC to achieve a balance between energy and power density. By optimizing the electrode materials and electrolyte composition, HSCs can deliver high-energy storage capacity while still maintaining rapid charge/discharge capabilities. These advanced ES technologies offer promising solutions for applications requiring high power output, rapid response times, and long cycle life. Their different working principles make them ideal candidates for various applications. A comparative analysis of different ES devices is provided in Table 25 [73].

Supercapacitors, as outlined in Table 25, offer numerous advantages over traditional batteries, including higher power density, faster charging and discharging rates, better performance in low temperatures, and a longer cycle life. However, they do come with certain limitations, such as higher cost and lower voltage ratings. The issue of lower voltage ratings can be addressed by using a DC-DC converter, though this may result in additional power consumption. In comparison, batteries are more cost-effective, have a higher energy density, and a lower self-discharge rate. Table 26 [71,73] provides a concise overview of the key strengths and weaknesses of the energy storage systems discussed in this section. The field of supercapacitors is rapidly evolving, with ongoing efforts focused on enhancing their performance. Table 24 presents the main parameters for these components, obtained from the literature [73,74,75,76,77,78,79,80].

Table 27 provides a comprehensive overview of various supercapacitors, highlighting their unique electrodes and electrolytes, as well as their energy and power densities [81].

### 5.3. New Trends in Energy Storage

The current trend in storage technology is moving towards advanced metal–air and solid-state batteries, which offer enhanced performance and increased longevity. These batteries show minimal degradation, low leakage, high energy density, and reliable operation in extreme temperatures [99].

#### 5.3.1. Metal–Air Batteries (MABs)

**Figure 16:**
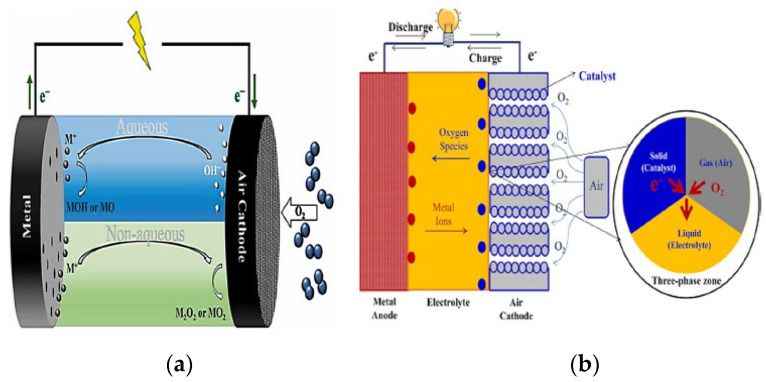
The schematic representation of metal–air batteries (**a**,**b**). Reprinted with permission from ref. [74].

MABs [100,101] operate on the principle of generating electricity through the reaction of a metal with oxygen from the air. These batteries typically consist of a metal anode such as zinc or lithium, a cathode made of porous carbon, and an electrolyte that allows ions to move between the anode and cathode, as seen in Figure 16. When the battery is discharged, the metal anode oxidizes and releases electrons, which flow through an external circuit to power a device. At the same time, oxygen from the air reacts with the metal ions at the cathode, producing oxide ions that combine with the electrons to form water or another byproduct. During the charging process, the metal ions at the anode are reduced back to their elemental form, while oxygen is released from the cathode. This cycle can be repeated multiple times, allowing the battery to be recharged and reused. Various electrolyte types—aqueous (protic), nonaqueous (aprotic), hybrid, and solid-state—can be used in these batteries. Lithium (Li), sodium (Na), and potassium (K) anode batteries are stable in non-aqueous systems, while magnesium (Mg), aluminum (Al), iron (Fe), and zinc (Zn) anode batteries are stable in aqueous environments and typically employ a hydrophobic protective layer to prevent electrolyte leakage. When the anode reacts with oxygen, electricity is generated. These batteries offer an exceptional energy density that outperforms traditional Li-ion batteries by 3–30 times, positioning them as a highly viable option for a dependable and enduring power supply.

These batteries have several advantages and disadvantages compared to traditional lithium-ion batteries some of them are reported in Table 28.

Table 29 provides an overview of recent advancements in six types of high-energy density MABs, each with a brief description. The aluminum–air (Al–air) battery uses aluminum as anode and oxygen as cathode, offering high energy density and a lightweight design. However, it has a short cycle life and is challenging to recharge. The calcium–air (Ca–air) battery demonstrates potential as a rechargeable battery utilizing calcium and oxygen, with the promise of achieving high energy density. Nevertheless, continued development is essential to improve efficiency and extend its lifespan. The magnesium–air (Mg–air) battery utilizes magnesium and oxygen, offering high energy density and lightness. However, further improvements are required to enhance its cycle life and efficiency. The iron–air (Fe–air) battery utilizes iron and oxygen, offering high energy density and lightness. However, it is still in the early stages of development, facing challenges with cycle life and efficiency. Lithium–air (Li–air) batteries utilize lithium metal and oxygen, offering incredible energy density potential. However, despite their promise, this battery is still in the research phase, encountering obstacles such as limited cycle life, low efficiency, and instability. Zinc–air (Zn–air) batteries utilize zinc with oxygen to generate electricity, are highly energy-dense but non-rechargeable, and have a short shelf life. Despite early development stages, they show potential for future energy storage with further research needed to overcome limitations and improve practicality for wider use [100,101]

#### 5.3.2. Thin Film Batteries (TFBs)

**Figure 17:**
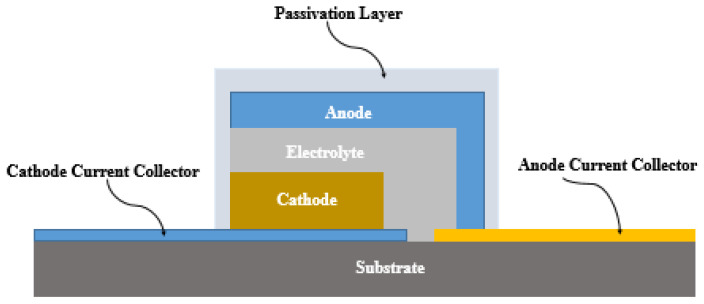
TFB basic construction.

Thin-film batteries (TFBs) represent advanced rechargeable solid-state batteries constructed using multiple thin layers of materials deposited on a substrate. These layers serve as the electrodes, electrolyte, and current collectors of the battery. The current collectors, typically composed of metals or conductive materials, are essential for collecting and conducting the current within the battery. Figure 17 provides a visual representation of the basic construction of a TFB [102].

**Figure 18:**
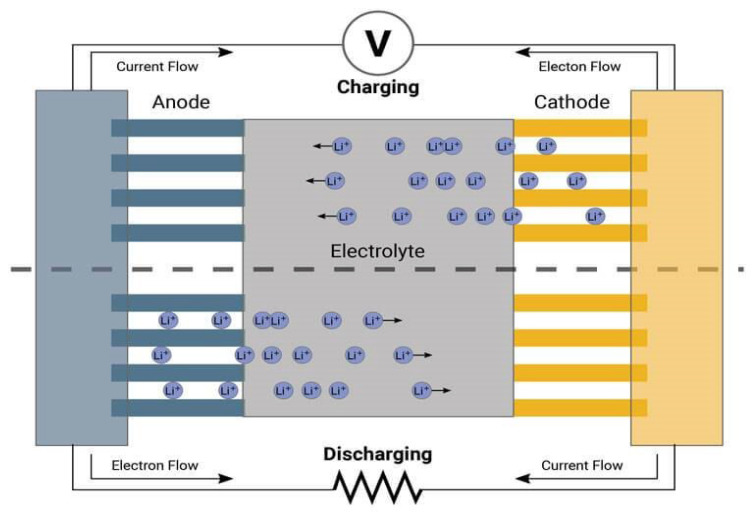
Basic process of charging/discharging battery schematic.

The working principle of a thin film battery involves the movement of ions between the electrodes through the electrolyte to generate electrical energy. When a thin film battery is charged, ions are extracted from one electrode and migrate through the electrolyte to the other electrode, where they are deposited. This process creates an electric potential difference between the electrodes, which can be used to power external devices. During discharge, the ions are released from the electrodes and travel back through the electrolyte, generating an electrical current. A basic process of charging/discharging the battery schematic is detailed in Figure 18. The electrode/electrolyte interfaces are intimately connected, with a thin electrolyte layer enabling rapid charging and discharging. Additionally, the electrode material is densely packed and streamlined, resulting in increased energy densities, minimal self-discharge (<1% per year), and an extended cycle life.

Table 30 [103] offers a comprehensive summary of the most recent developments in TFB technology, including advancements in current collectors, electrodes, and electrolyte materials.

TFBs are known for their high energy density and ability to deliver a powerful output, making them ideal for applications that need a compact energy solution. Compared to traditional batteries, TFBs have a significantly longer lifespan and exhibit greater resistance to degradation over time. When combined with EHT technology, TFBs can operate even more efficiently, maximizing their performance and longevity. It is crucial to consider factors like energy density, power output, lifespan, and cost when selecting a battery type to ensure optimal performance for your specific needs.

## 6. Enhancing WSN Performance through Energy Harvesting Techniques (EHT)

A sensor node typically uses rechargeable batteries as the main energy source when powering sensor nodes. However, rechargeable batteries show limitations, due to factors such as limited power capacity, leakage, power loss at higher temperatures, and short high-current pulses. To extend the WSN’s lifetime in remote areas, new sensor nodes are equipped with harvesters, which opportunistically acquire small amounts of energy within the immediate surroundings where these sensor nodes have been placed. Sensor nodes that support EH generate a new class of WSNs, referred to as Energy-Harvesting WSNs (EH-WSNs). By integrating a sensor node with a harvester, this enables the battery to be recharged indefinitely, ensuring a continuous power supply. The goal of EHT is to enable a sensor node to operate continuously without the need to replace the on-board battery. This is achievable when the sensor node is strategically designed to work in the proximity of ENO. Despite the benefits of EHT, challenges such as fluctuations, intermittence, low energy harvest, source unavailability, and low harvester efficiency must be addressed. Table 31 reports the detailed exploration of energy sources, harvester technologies, and material properties for efficient energy conversion. Hybrid EH systems and potential future research paths for sustainable integrated technology development are also highlighted. This section aims to bridge theoretical concepts with practical applications in EH-WSNs for environmental monitoring.

### 6.1. Radiant Energy

Radiant energy, also known as electromagnetic radiation, is a form of energy that travels through space in the form of waves. This ubiquitous energy emitted by sources like the Sun, and Earth, as well as human-made sources, plays a crucial role in providing light, heat, and power to numerous devices and technologies. In this section, we will delve into the different types of radiant energy, discuss their unique properties, and highlight their significance for environmental monitoring purposes.

#### 6.1.1. Solar Cell EH-WSN

**Figure 19:**
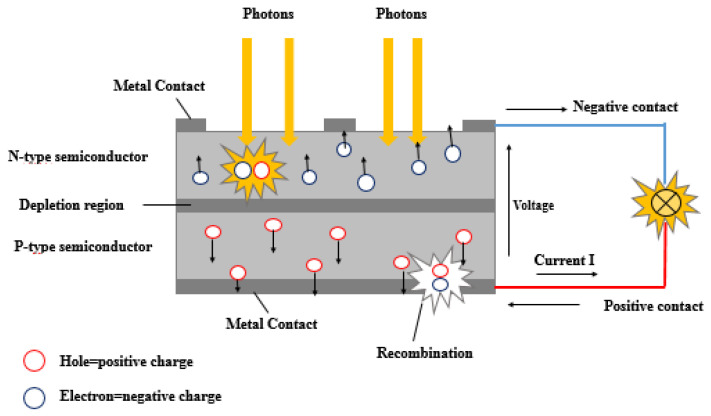
Principles of operation of a solar cell constituted by a single P–N junction.

**Figure 20:**
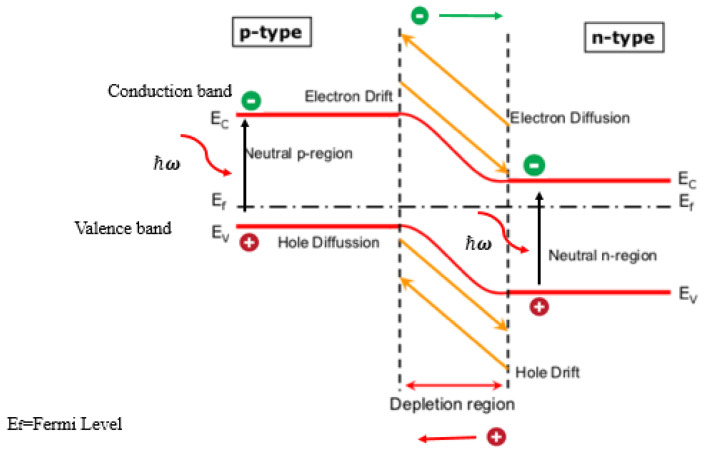
Bandgap structure of a P–N junction and the absorption process of photons.

Solar cells (SCs) operate by converting sunlight into electricity through the generation and separation of charge carriers within a material that is responsive to the photovoltaic effect (PVE), a physical process that enables the conversion of light into electrical energy. In Figure 19, a SC is formed by the combination of two unique layers, the P-type layer and the N-type layer, which combined create a junction known as the P–N junction. The P-type layer contains an abundance of positively charged holes (lack of electrons), while the N-type layer contains an excess of negatively charged electrons. When sunlight impacts on the SC surface, the photons with energy (hν) greater than that of the semiconductor’s bandgap Eg (hν > Eg) transfer energy to the electrons in the N-type layer and create electron–hole pairs as highlighted in Figure 20. Due to the built-in electric field at the P-N junction, the electrons are pushed towards the N-type layer, while the holes move towards the P-type layer. This separation of charges creates a voltage difference between the two layers. An external circuit is created by connecting electrical contacts to the P-type and N-type layers, enabling the generation of a voltage difference [116].

**Figure 21:**
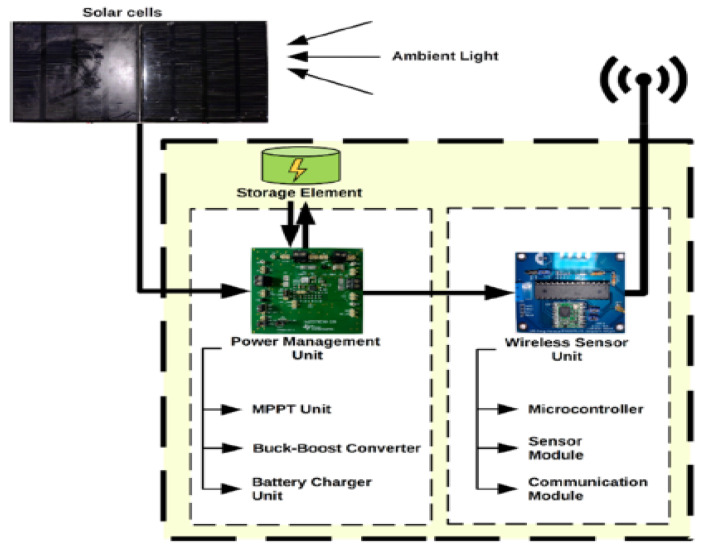
The proposed ULPD-WSN system diagram. Reprinted with permission from ref. [117].

In Figure 21, a SC represents a basic building block, which is then combined with other cells to form a module. Multiple modules are assembled to create a solar array. This array generates direct current (DC) to power sensor nodes without the need for extra circuitry to convert alternating current (AC) to DC. The PMU consists of a Maximum Power Point Tracking (MPPT) circuit, a buck-boost converter, and a battery charger. PMU aims to provide stable and efficient charging and discharging rates to the storage element while accommodating the load from the WSN.

**Figure 22:**
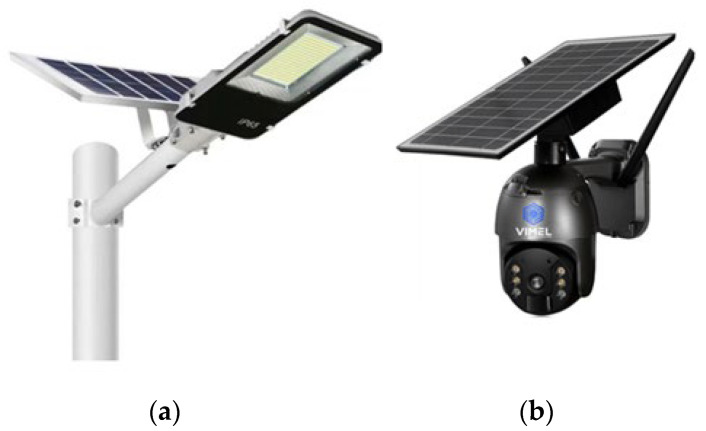
Solar cells integrated into outdoor lighting (**a**) and security cameras (**b**).

The use of a MPPT [118] controller is crucial in optimizing the performance of SCs under varying irradiance and temperature conditions, ensuring they operate at their maximum power point $P_{max}$. Furthermore, given the intermittent nature of solar energy, the inclusion of an energy storage system is essential to store excess energy for use during periods of low sunlight. Additionally, implementing a DC-DC converter is necessary to match the voltage output of the solar panels to the required voltage of the batteries, ensuring efficient and effective energy storage. On the other hand, the WSN includes a microcontroller unit (MCU), a sensor module, and a communication module, all working together to encode and transfer sensor data seamlessly. As reported in Table 31, solar power shows the highest power density in outdoor conditions, which makes it a top choice for sensor nodes. However, its power density may decrease when clouds eventually cover the cells or when the sensor is placed in indoor conditions. In outdoor conditions, solar cells can be integrated into outdoor lighting and security cameras, as in Figure 22a,b, and other equipment, providing a reliable and independent source of power.

The Shockley–Queisser limit sets the maximum efficiency η of a SC at around 30%. However, SCs with multiple layers of P–N junctions have the potential to surpass this limit, theoretically reaching efficiencies of up to 86.8%. Table 32 provides a description of the most important parameters $V_{oc}$, $J_{sc}$ and FF useful to increase solar cell efficiency.

#### 6.1.2. Evolution of SCs: From First to Third Generation

Currently, SCs are categorized into three generations based on the materials and technologies. Table 33 [116] offers an overview of the materials commonly utilized in SCs, highlighting their respective benefits, limitations, and comparative efficiencies. Despite significant advancements in SC technology, the current efficiency level remains below the theoretical Shockley–Queisser limit. The first-generation cells include monocrystalline, polycrystalline, and thin film silicon. The second generation includes a-Si, CdTe, CdS, CIS, and CIGS technologies. Third-generation cells use organic semiconductors, quantum dots, quantum wells, and perovskites to achieve high efficiency at a low cost, potentially exceeding the Shockley–Queisser limit. Single-crystalline silicon (c-Si) is known for high efficiency and stable performance, while microcrystalline silicon (mc-Si) offers a lower-cost alternative with good efficiency, though less stability. Thin-film silicon (tf-Si) is flexible and lightweight, suitable for weight and portability-sensitive applications. Amorphous silicon (a-Si) is cost-effective and easy to produce, but generally has lower efficiency compared to c-Si and mc-Si. In Table 34, each type of silicon-based solar cells has unique advantages, making them suitable for different applications and scenarios.

In Table 35, the second generation of solar cells represents a significant advancement in technology, providing higher efficiency, enhanced performance, and reduced production costs when compared to first-generation solar cells.

**Figure 23:**
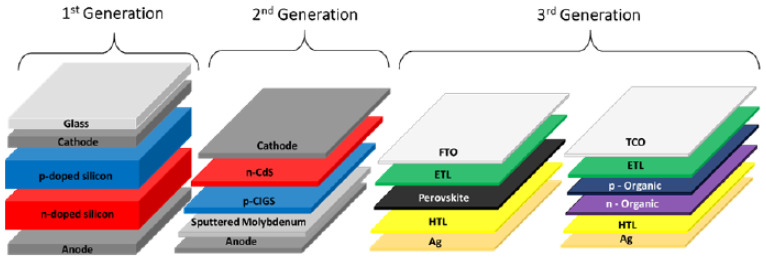
Overview of different generations of solar cells: 1st generation, 2nd generation (commercial thin films), and 3rd generation (emerging thin films). Reprinted with permission from ref. [144].

The SCs highlighted in Table 36 [140,141,142,143,144,145] are part of the third generation of SCs’ technology. These sophisticated SCs use advanced materials to improve energy conversion efficiency, while simultaneously reducing manufacturing costs. Figure 23 reports an overview of different generations of SCs: 1st generation, 2nd generation (commercial thin films), and 3rd generation (emerging thin films).

#### 6.1.3. Challenges and Future Directions for Emerging SCs Technologies

SCs represent a technology with great promise; however, challenges such as restricted efficiency, high manufacturing costs, and durability concerns must be addressed in order to effectively enhance SC technologies. To overcome these obstacles, it is crucial to explore innovative materials like perovskites, which show promise in boosting the efficiency of solar cells, as well as to adopt new fabrication methods and manufacturing processes [110].

### 6.2. Radio Frequency (RF)-EHWSN

**Figure 24:**
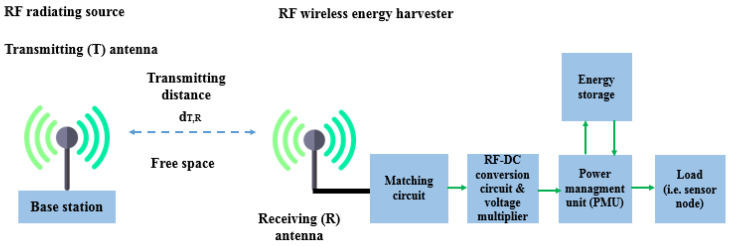
Schematic blocks of a radiofrequency energy harvesting system. It includes an antenna, a matching circuit, a rectifier, a voltage multiplier, a power management unit, an energy storage unit, and a load.

With the rapid growth of wireless technologies, the wireless power density is increasing due to various electromagnetic sources like mobile base stations, TV towers, and Wi-Fi routers. Using RF energy to power small sensor nodes that can operate on just microwatts of power has become popular to save costs and replace batteries. For this reason, signals from various sources, especially in urban areas, can be converted into electricity using a rectenna (rectifying antenna). This component efficiently converts RF signals into usable DC power. In Figure 24, the architectural design of a rectenna can be divided into distinct parts [110].

The antenna is essential to the rectenna, receiving radiofrequency signals when it is resonant at the wavelength of the electromagnetic wave. A matching network ensures impedance alignment with rectifying circuitry to prevent signal reflection. The rectifying circuitry converts AC radiofrequency signals into DC power. Advanced rectifier options, like Schottky diodes, tunnel diodes, and spin diodes, offer high efficiency with low incident power levels. Schottky diodes are preferred for RF energy harvesting due to their low threshold voltage and junction capacitance, enabling efficient operation at RF frequencies. Researchers are studying materials like graphene to improve rectification and efficiency, with Metal–insulator–graphene (MIG) [168] diodes achieving over 90% efficiency at 170 GHz. A filtering network is applied to smooth out any ripple or residual AC component in the rectified output. A PMU control ensures a consistent power supply, extending the battery lifetime. The PMU includes a DC-to-DC charge pump that steps up lower voltages from the battery to power device components. Energy storage components like capacitors or rechargeable batteries store the converted DC power for later use, ensuring a stable power supply for a sensor node. Energy densities can vary from 0.01 μW/cm^2^ to 100 μW/cm^2^, and in some cases, where a dedicated RF transmitter is available, the RF power density can reach 300 μW/cm^2^. The efficiency of each block is crucial for the overall power conversion efficiency of an RF-EHWSN. Therefore, all elements play an equally important role in achieving optimal performance [169]. To optimize overall efficiency, it is crucial to carefully evaluate the placement of the RF transmitter, as well as the distance and orientation between the harvester and the RF transmitter, and ensure that the receiving antenna is accurately aligned and matched about beam pointing and polarization. The different power densities of RF bands can be found in Table 37.

**Figure 25:**
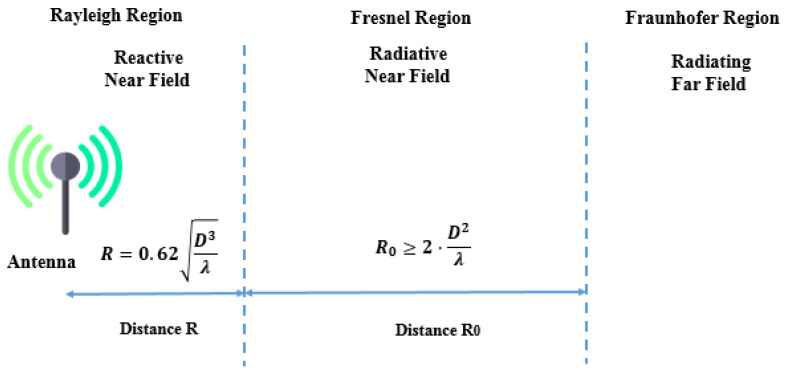
Near-field and far-field regions for an antenna.

The near field and far field, reported in Figure 25, are regions of the electromagnetic (EM) field around a transmitting antenna. These categories differ in the way they transfer energy across distances. Near-field sources work at close distances, usually within a few wavelengths of the transmitter, while far-field sources transmit energy over greater distances.

**Figure 26:**
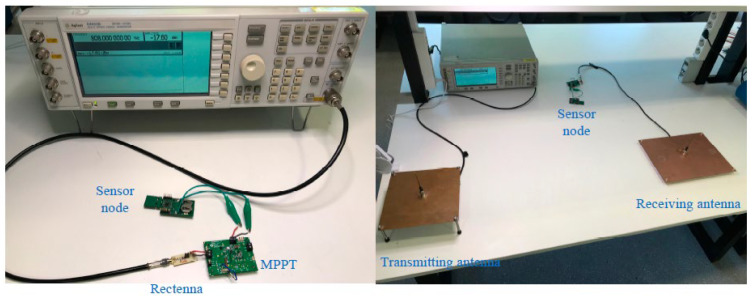
Image showing the setups for powering the sensor node using an RF generator (**left**) and a monopole antenna (**right**) for the RF harvester input. Reprinted with permission from ref. [173].

**Figure 27:**
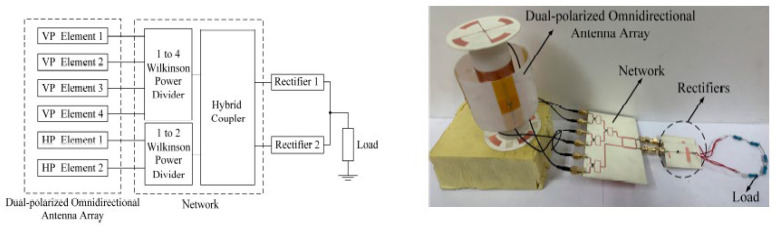
Image showing the setups for powering a load using a rectenna array designed at 2.45 GHz (**left**) and a fabricated rectenna array (**right**). Reprinted with permission from ref. [174].

**Figure 28:**
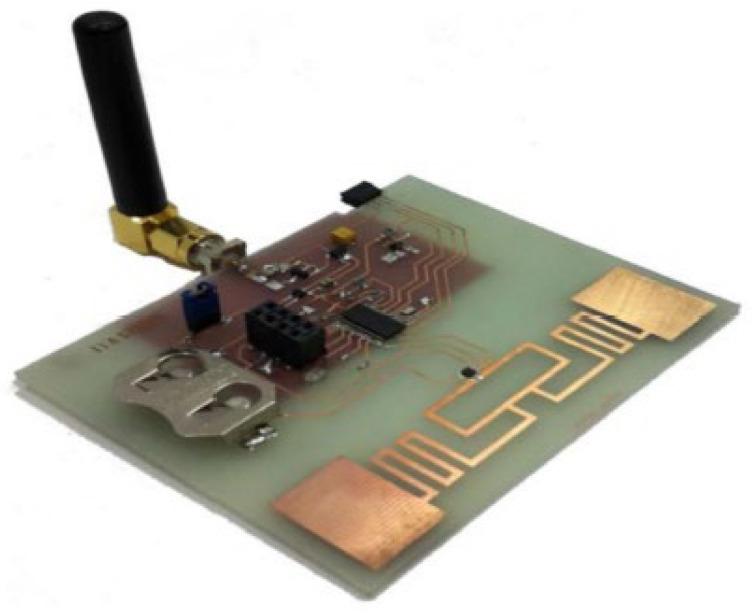
Module for smart environmental sensing (RAMSES) for agriculture IoT sensor. Reprinted with permission from ref. [175].

Electromagnetic induction and magnetic resonance are frequently utilized in near-field applications to generate electricity and power devices located within close proximity to each other. In reference [171,172], near-field RF-EH is detailed as taking unintentional RF energy ranging from 10 kHz to 1 GHz, and converting it into usable energy within a railway environment. By utilizing a metamaterial with a broad frequency range of 350 MHz and an 8.5 kΩ load, it was possible to achieve a remarkable energy transfer efficiency of up to 84%. In far-field applications, antennas can receive RF signals and convert them into power through rectifier circuits. However, the power received by omnidirectional antennas decreases with distance, limiting the amount of energy available for harvesting. RF energy harvesters are more effective in urban and suburban areas, where there are abundant energy sources in close proximity to RF sources. Figure 26, Figure 27 and Figure 28 show some examples of RF EH.

#### Challenges and Future Directions for Emerging RF-EHWSN Technologies

While RF-EH technology has made significant advancements in recent years, there are still several practical challenges that must be overcome. One major challenge is the limited range and efficiency of RF harvesters, which can limit the power supply of sensor nodes in real-world scenarios. The variability in RF signal strengths and frequencies across different locations also adds complexity to EH efforts. Furthermore, the integration of RF-EH with existing WSN infrastructure and traditional protocols poses another significant challenge that needs to be addressed. Future research directions for RF-EHWSN include improving the efficiency, optimizing antenna design and placement, developing novel materials with improved RF energy absorption properties, developing adaptive EH algorithms and protocols, exploring alternative energy sources for WSNs, and exploring new energy storage solutions that can better meet the power requirements of sensor networks. Table 38 provides an overview of RF-EHWSN design and implementations [110].

### 6.3. Infrared-Frequency Rectifying Antenna

**Figure 29:**
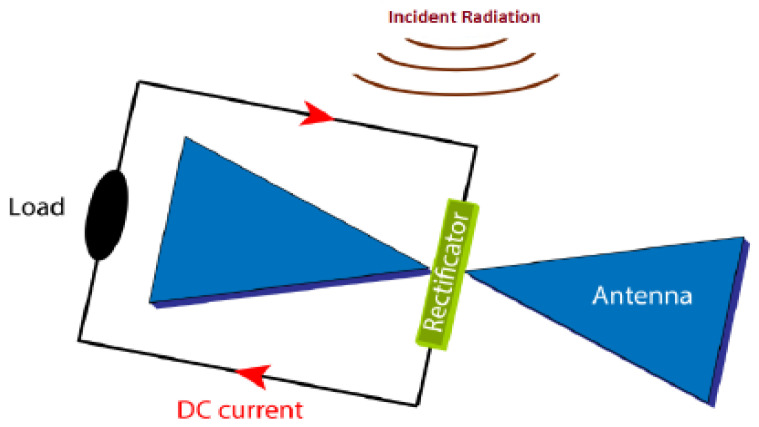
A basic architecture of a typical THz rectenna.

Contrary to solar energy, which can be limited by weather factors like clouds and rain, as well as the absence of sunlight at night, infrared radiation (MID-IR around 10 μm, 28.3 THz) is consistently available 24/7. Signals from various infrared sources can be converted into electricity using a rectenna (rectifying antenna) or an Infrared-frequency Rectifying Antenna (IRA), a type of antenna that, combined with an ultra-high-speed diode, converts infrared radiation into direct current (DC). This section offers a comprehensive overview on specific aspects, and advancements on IRA. The research focuses on quantum tunneling in both single insulator and multi-insulator diodes, ballistic transport as well as antennas at the nanoscale, current challenges, and future prospects. The working principle of an IRA involves capturing infrared radiation with an antenna, and converting it into electrical energy through rectification. This energy is subsequently utilized to power sensor nodes. The main components of an IRA in Figure 29 include an antenna, a rectifier, and a load [193,194,195,196,197].

**Figure 30:**
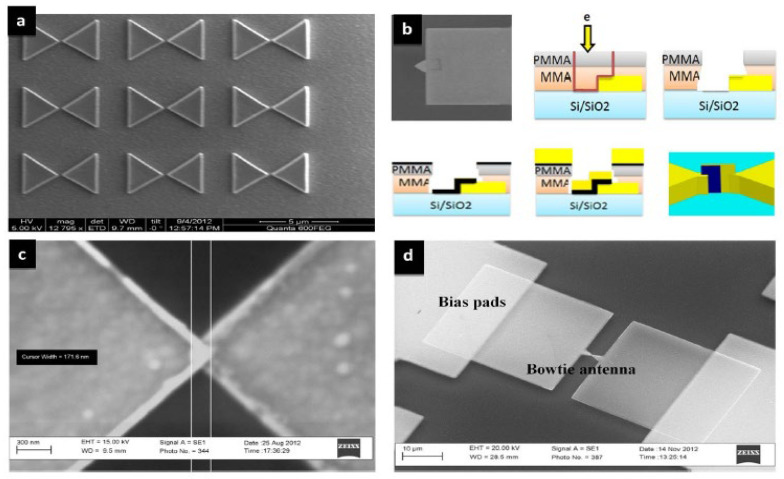
The fabrication process of nanoantennas and the rectenna device. Panel (**a**) highlights a SEM image of the nanoantenna array created using EBL. Panel (**b**) outlines the overlap fabrication steps: (i) creation of the first antenna arm, (ii) exposure of the second arm using electronic beam layer (EBL), (iii) removal of exposed resist with methyl isobutyl ketone (MIBK) and Isopropyl alcohol (IPA) developer mixture (ratio 1:5:3), (iv) deposition of 0.7 nm of oxide through atomic layer deposition (ALD), (v) sputtering of the second arm, (vi) completion of device after liftoff process with acetone. Panel (**c**) displays a SEM image of the fabricated overlap, while panel (**d**) presents a SEM image of the antenna-integrated diode. Reprinted with permission from ref. [219,220].

One of the significant advantages of using IRAs is their capability to capture energy from a wide range of ambient infrared sources, such as sunlight, waste heat from household appliances, Earth’s surface, and even body heat. When the size of the antenna is specifically designed to capture the MID-IR electromagnetic wave (EMW) in resonance condition, a flow of electrons known as Surface plasmon polaritons (SPPs) propagate along the interface between the metal and dielectric material. SPPs rapidly decay as the distance from the metal surface increases. This plasmonic signal creates an alternating current (AC) signal on the antenna surface, which is rectified through an ultra-fast diode (planar or geometric) generating a DC current. In the THz range, a diode requires a short response time (approximately 10^−15^ s RC time constant). One possible solution is to create a diode that activates when it reaches the open circuit voltage of the antenna, effectively improving power efficiency. A low-pass filter (LPF), is used in EH applications to stabilize the rectifier’s output voltage and ensure consistent DC voltage. A DC-DC converter is necessary to adjust voltage levels from the LPF for a storage device like a rechargeable battery. An optimal impedance match between the antenna and diode is key for efficient rectenna conversion. Planar and geometric diodes achieve the same function of allowing current to flow in one direction while blocking it in the other direction. For planar diodes, Metal-insulator-metal (MIM) and Metal Multi-Insulator Metal (MI^n^M, n represents the number of ultrathin insulator layers) play a crucial role in converting MID-IR electromagnetic radiation into electrical power. MIMs work by allowing electrons to tunnel through a thin insulating layer between two different metal layers, producing a nonlinear current-voltage characteristic. This makes MIM diodes ideal for high-frequency applications. The insulating layer acts as a barrier, controlling the flow of current in one direction while blocking it in the other. In MIM technology, as shown in Figure 30, antennas use the same metal material, except for the contacts at the tip, which are made from a different material. This generates an asymmetry between the materials, enhancing the rectification process [193,194,195,196,197]. The passage of electrons through the insulating layer creates current flow in the device, which can be regulated by the open circuit voltage at the antenna ends to capture and convert energy more effectively. Optimizing MIM rectifier performance involves adjusting insulating layer thickness, material properties, and electrode metals. Table 39 and Table 40 display the figures of merit (FOM) for the MIM/MInM diodes, including asymmetry (Asym), nonlinearity (NL), responsivity (S), current density (JON), turn-on voltage (TOV), and zero-bias resistance (ZBR). These parameters are crucial for evaluating the performance of MIM/MI^n^M diodes. In recent years, there has been significant progress in the development of MIM diodes for THz rectennas.

The theoretical conversion efficiency of this technology surpasses the limit set by Shockley–Queisser (S&Q) [198,199,200,201,202,203,204]. However, although there have been notable advancements in antennas and diode rectifiers, there are still several technological challenges that need to be overcome at 28.3 THz. The antenna design, materials utilized, and the rectification technology implemented determine the improvement of the IRA. Researchers have made several efforts to improve the efficiency and performance of MIM diodes for THz rectennas. Table 39 reports recent advancements in the field of MIM diode for THz rectennas, opening new possibilities in practical applications [198,199,200,201,202,203,204].

**Table 39:** Summary of recent achievements in the field of MIM diode for THz rectennas.

Material	Cut-Off Frequency	Thickness	J_ON_	Asym	NL	S (V^−1^)	Zero Bias S (V^−1^)
Cu (100 nm)-CuO-Au (100 nm) (0.0045 μm^2^) [199]	28.3 THz	CuO (0.7 nm) Au/Cu (100 nm)	-	-	-	6	4
Ti-TiO_2_-Al (21,287 µm^2^) [205,206,207,208]	Up to 150 THz	TiO_2_ (9 nm)	10^−1^ A/cm^2^	-	6.5	18	-
Ti-TiO_2_-Pt (21,287 µm^2^) [205,206,207,208]	Up to 150 THz	TiO_2_ (9 nm)	10^−0^ A/cm^2^	-	15	15	-
Nb/Nb_2_O_5_/Pt [205,206]	Up to 150 THz	Nb_2_O_5_ (15 nm)	-	1500	4	20	-
Nb/Nb_2_O_5_/Cu [205,206]]	Up to 150 THz	Nb_2_O_5_ (15 nm)	-	1500	8	20	-
Nb/Nb_2_O_5_/Ag [205,206]	Up to 150 THz	Nb_2_O_5_ (15 nm)	-	1500	8	20	-
Nb/Nb_2_O_5_/Au [205,206]	Up to 150 THz	Nb_2_O_5_ (15 nm)	-	1500	8	20	-
Au/Al_2_O_3_/Pt [205,206,207,208,209,210]	Up to 28.3 THz	Al_2_O_3_ (1.4 nm) Au/Pt (100 nm)	-	-	6	-	10
Ni-NiO-Ag (3.1 × 10^−4^ µm^2^) [211]	Up to 343 THz	NiO (6 nm)	-	5	3	8.5	5.8
Pt-SiCl_3_-(CH_2_)_17_-CH_3_ -Ti (100 μm^2^) [212]	Up to 150 THz	SiCl_3_-(CH_2_)_17_-CH_3_ (2.23 nm)	-	117.8	6.8	20.8	8.0
Nb/TiO_2_/Pt [213]	Up to 30 THz	TiO_2_ (13 nm)	-	80	3.5	-	-
Nb/Nb_2_O_5_/Ni [213]	Up to 150 THz	Nb_2_O_5_ (15 nm) Nb/Ni (90–100 nm)	1 × 10^−10^ A/cm^2^	396.5	7.1	8.5	-
Nb/Nb_2_O_5_ (15 nm)/Au [214]	Up to 150 THz	Nb_2_O_5_ (15 nm) Nb/Au (90–100 nm)	-	1430.8	8.0	7.0	-
SrTiO_3_ (STO)/Al_2_O_3/_SrTiO_3_ (STO) [215]	Up to RF	-	5 × 10^−9^ A/cm^2^	-	-	-	-
Cu-CuO-Cu (2 × 2 μm^2^) [216]	Up to 150 THz	CuO (2 nm) Cu (100 nm)	-	-	-	4.497	-
Pt/Al_2_O_3_/Al [217]	Up to 150 THz	Al_2_O_3_ (6 nm) Pt/Al (100 nm)	-	110 for AP-CVD 30 for PEALD	6 for AP-CVD 30 for PEALD	9 for AP-CVD 22 for PEALD	-
Al-Al_2_O_3_-Au [218]	Up to 60 THz	Al/Au (65 nm)	4.0 μA/cm^2^	-	-	14.46	-
Al-Al_2_O_3_-Cr [219]	Up to 28.3 THz	Al_2_O_3_ (3 nm) Al/Cr (100 nm)	2 × 10^−4^ A/cm^2^	-	3.1	-	-

**Figure 31:**
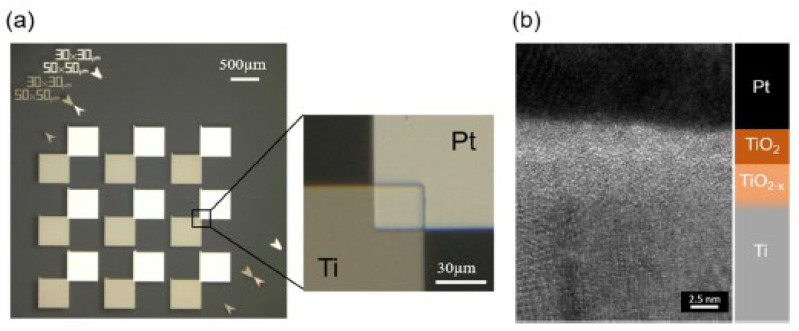
(**a**) MI^2^M fabricated on a silica substrate Si, where the metal pads, Pt and Ti, are overlapped for an area of 900 μm^2^, and (**b**) cross-sectional view of the diode part, obtained by TEM. Reprinted with permission from ref. [199].

On the other hand, MI^n^M diodes represent an advanced version of MIM rectifiers (Figure 31), where multiple insulating layers, incorporated between the metal electrodes, enhance the rectification efficiency and frequency response of the diode. The observed advantages over MIM are low leakage current, high breakdown voltage, and low operating voltage. The careful selection of insulators with extremely narrow bandgaps ensures that only electrons possessing a specific energy level can successfully tunnel through. This leads to a significantly enhanced rectification ratio. In addition, the metals used for contacts can be of the same nature, as long as the electron affinities of the insulators are different. Table 40 reports recent achievements in the field of Metal Multi-Insulator Metal (MI^n^M) diodes for THz rectennas.

**Table 40:** Summary of recent achievements in the field of Metal Multi-Insulator Metal (MI^n^M) diodes for THz rectennas.

Material	Cut-Off Frequency	J_ON_	Asym	NL	S (V^−1^)	Zero Bias S (V^−1^)	Resistance
W/Nb_2_O_5_ (3 nm)/Ta_2_O_5_ (1 nm)/W [221] W/Nb_2_O_5_ (1 nm)/Ta_2_O_5_ (1 nm)/W [221]	Up to 150 THz	- -	- -	- -	11 11	- -	- -
Cr (60 nm)/TiO_2_ (1.5 nm)/Al_2_O_3_ (1.5 nm)/Ti (60 nm) [222] Cr (60 nm)/TiO_2_ (0.75 nm)/Al_2_O_3_ (0.75 nm)/TiO_2_ (0.75 nm)/Al_2_O_3_ (0.75 nm)/Ti (60 nm) [222]	Up to 150 THz	- -	- -	6 7	3 90	- -	- -
Al (60 nm)/Ta_2_O_5_ (3–6 nm)/Al_2_O_3_ (1 nm)/Al (60 nm) [223] Al (60 nm)/Nb_2_O_5_ (3–6 nm)/Al_2_O_3_ (1 nm)/Al (60 nm) [223]	Up to 150 THz	10^2^A/m^2^	18	7.5	9	-	-
Co/Co_3_O_4_ (1.1 nm)/TiO_2_ (1.05 nm)/Ti [224]	Up to 30 THz	10^5^ A/cm^2^	-	-	4.4	2.2	18 KΩ
Ti/TiO_2_ (1 nm)/ZnO (0.5 nm)/Al [225]	Up to 17.4 THz	-	-	-	5.1	1.6	312 Ω
Cr/Cr_2_O_3_ (2 nm)/HfO_2_ (2 nm)/Al_2_O_3_ (2 nm)_/_Cr [224,225,226] Cr/Cr_2_O_3_ (2 nm)/Al_2_O_3_ (2 nm)/HfO_2_ (2 nm)/Cr [226]	Up to 30 THz	- -	5 4	4 5	- -	- -	- -
Pt (70 nm)/TiO_2_ (2 nm)/TiO_1.4_ (0.6 nm)/Ti (50 nm) [219]	Up to 30 THz	4.2 × 10^6^ A/m^2^	7.3	-	-	-	-
Cr (100 nm)/Cr_2_O_3_ (3 nm)/Al_2_O_3_ (3 nm)/Ag (100 nm) [227]	Up to 30 THz	3 mA/cm^2^	>280	-	-	-	-
Cr (100 nm)/Al_2_O_3_ (2 nm)/HfO_2_ (2 nm)/Cr [228]	Up to 30 THz	70 µA/cm^2^	9	10	4.8	-	-
ZCAN (ZrCuAlNi 150 nm)/HfO_2_ (5 nm)/Al_2_O_3_ (3 nm)/Al (150 nm) [229]	Up to 30 THz	-	>10	>5	-	-	-
Pt (150 nm)/HfO_2_ (1.5 nm)/TiO_2_ (1.5 nm)/Ti (150 nm) [230,231]	Up to 30 THz	-	10	>5.5	2 × 10^4^	-	0.1 MΩ
Pt (150 nm)/Al_2_O_3_ (1.5 nm)/TiO_2_ (1.5 nm)/Ti (150 nm) [230,231]	Up to 30 THz	-	17	>5.5	2 × 10^4^	-	0.1 MΩ
Ni (150 nm)/NiO (1.5 nm)/ZnO (1.5 nm)/Cr (150 nm) [232]	Up to 30 THz	-	16	-	-	-	-

**Figure 32:**
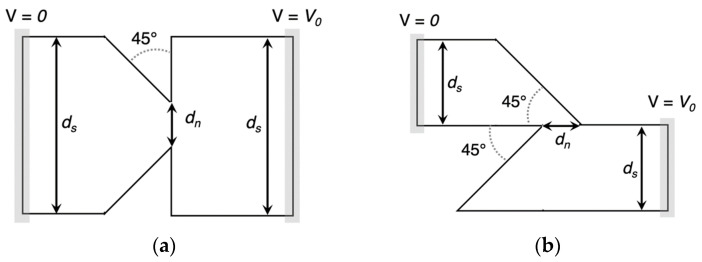
(**a**) Relevant dimensions of an inverse-arrowhead diode; and (**b**) a Z-diode. Reprinted with permission from ref. [233].

Graphene-based geometric diodes (GGD) are a promising technology for THz rectennas due to their high electron mobility, excellent thermal conductivity, and flexibility. In a graphene-based geometric diode, a graphene nanoribbon is precisely patterned into a specific geometric shape, such as a zigzag or armchair structure, as shown in Figure 32. This unique design introduces a bandgap into the graphene material [233].

The functionality of the diode relies on the asymmetrical tunneling process through the openings in the graphene sheet. When the MID-IR radiation is incident on the graphene surface, the electrons in the graphene sheet are excited and can move through the openings of width $d_{n}$, as shown in Figure 32a,b. This results in a selective flow of current in one direction while restricting it in the opposite direction, leading to a rectification effect. Table 41 reports recent achievements in the field of graphene-based geometric diodes for THz rectennas.

#### 6.3.1. Challenges and Future Directions in the Field of Antennas

Designing antennas for the MID-IR frequency range presents challenges due to unique spectrum characteristics. Special materials and design considerations are needed for efficient reception, which are still being explored. One main challenge is finding suitable materials that operate efficiently at these frequencies, as many traditional materials have high losses in the MID-IR range. Innovative antenna designs and optimization techniques are necessary to achieve high efficiency. Advanced nanofabrication techniques are required for creating miniaturized antennas in the THz range. Integrating THz antennas with diode rectifiers is essential for a functional rectenna system, but impedance matching remains a challenge. Surface reflection and scattering can result in losses, emphasizing the need to minimize radiation leakage and ohmic resistance. Future directions for THz antennas include exploring new materials like metamaterials, plasmonic materials, and graphene to enhance performance. Researchers are exploring new antenna shapes capable of capturing the MID-IR signal more easily. Advances in fabrication techniques, such as additive manufacturing, nanolithography, atomic layer deposition (ALD) and 3D printing, are enabling the development of complex and miniaturized antennas for the MID-IR frequency range. These techniques allow for precise control over antenna geometry, resulting in improved performance and efficiency [238,239,240,241].

#### 6.3.2. Challenges and Future Directions in the Field of THz Diode

Currently, most THz diodes operate up to the MID-IR frequency. One of the main challenges in the field of THz diodes is to push the operating frequencies to higher limits. Another challenge is to improve the efficiency of THz diodes, which is currently less than 1% [198]. This includes reducing power consumption, increasing output power, and maximizing the conversion efficiency of electrical power into THz radiation. Some potential areas of development and research include exploring different materials and material combinations for the diode layers to improve conductivity, bandgap, and electron transport properties, which can lead to higher energy conversion efficiencies. Improving the interface between the metal and insulator layers can help reduce leakage current, improve charge carrier injection, and enhance overall device performance. Integration and fabrication techniques for THz diodes often involve complex processes, such as depositing ultra-thin insulator layers, precise electrode patterning, and atomic layer deposition (ALD). Achieving excellent alignment between multiple layers is also important. Developing reliable and scalable fabrication techniques is crucial for consistent performance and enabling large-scale production [242,243].

#### 6.3.3. Implementing Machine Learning in Emerging EHT

Machine Learning (ML) for emerging energy harvesters is a topic that involves using artificial intelligence algorithms to optimize the efficiency and performance of EH devices [244]. ML algorithms can be used to analyze data from energy harvesters and make predictions or recommendations to improve their energy conversion efficiency. In particular, ML can be used to optimize the placement of solar panels to maximize sunlight exposure, or to adjust the operating parameters of a thermoelectric generator (TEG) based on real-time environmental conditions. In addition, ML can help to identify new materials and design strategies for EH devices that can further increase their performance and reliability.

ML algorithms in EH have various other applications, such as predicting energy source availability, managing harvested energy, and predicting energy intensity, as detailed in the survey in [245]. Predicting energy source availability is a key use of ML techniques in energy collection. In a research study, linear regression and artificial neural networks (ANN) were used to identify and switch between expected and unexpected radio frequency (RF) energy sources in order to enhance energy harvesting (EH) by adjusting the wake-up and sleep schedule as needed [246]. AdaEM [247] employs ML to forecast user behaviors, energy consumption trends, and the availability of energy sources for managing power in wearable devices. A proof-of-concept study revealed longer intervals between recharging by utilizing energy harvested from light and movement. ML has the potential to uncover innovative materials suitable for diverse thermoelectric energy harvesting devices [248]. Various ML algorithms, including linear regression, support vector machine, random forest, and decision tree, were utilized in a study [249] to forecast the presence of RF energy at different points within a WSN. Specifically, the linear regression model established a threshold within the RF frequency band, ranging from 1805 to 1880 MHz, to determine when to activate the energy harvester or switch to sleep mode for optimal efficiency [250]. EH is commonly employed in wearable and assistive devices to power them, with the additional integration of ML techniques to enhance their capabilities. The study in [251] presented the use of supervised ML methods to predict harvestable energy from indoor lights. The model incorporates spectral information to accurately classify energy sources, demonstrating its ability to predict energy output for GaAs solar cells with a mean absolute error of less than 5%. Another study in [252] demonstrated a distributed ML framework where connected devices collect energy from the environment. Various ML algorithms have been explored to improve MPPT performance, including techniques for solar energy harvesters [253,254,255,256,257] and TEGs [258]. However, the computational demands of ML pose a challenge, leading to increased energy consumption. Therefore, the implementation of ML techniques in EH-WSNs devices is still in its early stages. Finally, ML can be also used for dynamic power management in sensor nodes to predict and optimize power consumption. By predicting power consumption, it can adjust power settings dynamically to optimize energy efficiency. Optimization algorithms take into account current workload and performance requirements to adjust power levels in real-time. Anomaly detection ML algorithms can identify and address inefficient or malfunctioning components in a system by detecting anomalies in power consumption. This optimization helps reduce energy waste and improve system performance. Adaptive control ML algorithms continuously monitor and adjust power settings based on changing system conditions to optimize power consumption in real-time.

#### 6.3.4. Hybrid Energy Harvesting

Hybrid Energy Harvesting (HEH) combines various energy sources, such as solar, RF, IR, etc., to produce a higher electrical output. By leveraging the strengths of each energy source, HEH systems are able to operate more efficiently and effectively, making them ideal for a wide range of applications, including remote sensing, environmental monitoring, and IoT devices. For instance, while SCs only utilize a fraction of the incoming light, the photons that fall outside the material bandgap are typically lost as heat. By integrating thermoelectric or infrared techniques with photovoltaic systems, it is possible to capture a higher percentage of the incoming energy, boosting the overall power output [259]. The study in [260] demonstrated the mathematical modeling and numerical analysis of heat transfer and temperature distribution for the calculation of PV-TEG hybrid energy generation. It was found that a theoretical energy efficiency of 23% is achievable in PV-TEGs, which is higher than that of single energy harvesters. The system can achieve a power conversion efficiency of 16.3% by utilizing a 15 °C temperature gradient in a TEG combined with a Photovoltaic (PV) system [261]. HEH presents a promising solution by combining the advantages of various energy harvesting techniques. On the other hand, this approach introduces complexity in designing the coupling interface and power conditioning circuit. The difficulties associated with the coupling effect between Triboelectric Nano Generator (TENG)-Thermoelectric Generator (TEG) and TENG-Photovoltaic (PV) systems were explored for a metal-semiconductor interface in [262]. This paper [263] describes the creation of a hybrid RF-Solar EH unit that utilizes both RF waves from the GSM 900 band and solar power. The hybrid system efficiently captures RF waves and solar energy to enhance the power generation capability and improve the tagging distance of the EM4325-based active Radio-Frequency Identification (RFID) tag operating at 919 MHz. Demonstrations have indicated the successful utilization of multi-source energy harvesters [264] for powering various electronic devices and sensors in real-world applications.

## 7. Conclusions

EHWSNs are becoming increasingly important in various applications, revolutionizing data collection and decision-making processes. To the best of our knowledge, only 30 papers have comprehensively detailed a completely self-sustainable technology focused on micro-scale energy systems. This paper reviews the advancements enabling the perpetual operation of EHWSNs. Despite efforts to reduce energy consumption, the energy required for continuous operation remains high. Various energy-saving techniques were explored, with strengths and weaknesses identified. PMU and DC-DC boost converters play a crucial role in maximizing efficiency. Advanced ES solutions are being researched to ensure continuous operation. Different protocols are compared for energy efficiency, with harvesters explored for unlimited EHWSN lifetime. One potential future research direction to enhance EHWSNs’ efficiency is to develop techniques that can utilize multiple energy sources simultaneously. By combining different types of energy harvesters, EHWSNs could increase overall energy generation and storage capabilities, improving longevity and sustainability. Open issues and future research directions are being addressed to enhance efficiency and longevity. Today’s technology may not be sufficient, but potential paths towards efficiency are discussed.

## Figures and Tables

**Table 1:** Typical power requirements of some sensor nodes.

	IRIS [2]	MicaZ [3]	IMote2 [4]	SunSpot [5]	Waspmote [6]	WiSMote [7]
Radio standard	802.15.4/ZigBee	802.15.4/ZigBee	802.15.4	802.15.4	802.15.4/ ZigBee	802.15.4/ZigBee/6LoWPAN
Microcontroller	ATmega 1281	ATMEGA 128	Marvell PXA271	ARM 920 T	Atmel ATmega 1281	MSP430F5437
Sleep	8 μA	15 μA	390 μA	33 μA	55 μA	12 μA
Processing	8 mA	8 mA	31–53 mA	104 mA	15 mA	2.2 mA
Receive	16 mA	19.7 mA	44 mA	40 mA	30 mA	18.5 mA
Transmit	15 mA	17.4 mA	44 mA	40 mA	30 mA	18.5 mA
Idle	–	–	–	24 mA	–	1.6 mA
Supply	2.7–3.3 V	2.7 V	3.2 V	4.5–5.5 V	3.3–4.2 V	2.2–3.6 V
Average	–	2.8 mW	12 mW	–	–	–

**Table 2:** Compares of some energy sources available in the ambient.

Reference	Energy Source	Method	Merit	Power Density	Efficiency	Weakness	Applications
[10,11,12]	Light Energy	Photovoltaic	Predictable, Mature	5–100 mW/cm^2^ (Outdoor) 0.5–1000 μW/cm^2^ (Indoor)	30% 15%	Expensive, light not steadily available, Maximum Power Point Tracking (MPPT) is needed.	Biometric, agriculture, monitoring, ZNE building, indoor and portable devices
[13]	Radio-frequency energy	Rectenna	Continuously available, can carry and process information simultaneously	0.01–0.3 μW/cm^2^	±50%	Efficiency decreases with distance, impedance matching is needed	Sensor, nuclear, wirelessly powering
[14]	Thermal radiation is emitted by objects at moderate temperatures, typically within the range of 300 to 4000 K. This also encompasses the radiation emitted by the Earth’s surface.	Rectenna	Continuous available, waste heat can be used	60 mW/cm^2^	1%	Limited conversion efficiency, thermal losses, narrow bandwidth, challenges in design and fabrication, impedance matching needed	Energy harvesting, thermal imaging, remote sensing, communications, spectroscopy

**Table 3:** Operation states and consumption levels of a typical sensor node for EH-WSN [16,17].

State			
Module	Operation	Classifier	Consumption
Communication	RX (RF)	Active	Tens of mA
	TX (RF)	Active	Tens of mA
	Sleep	Inactive	μA
Computation	Processing	Active	Hundreds of μAMHz^−1^
	Memory access	Active	mA
	Sleep	Inactive	μA
Sensing	Sampling	Active	μA—hundreds of mA
	Warm-up	Active	μA—hundreds of mA
	Sleep	Inactive	μA

**Table 4:** Dynamic power reduction approach.

	Pros	Drawbacks
Supply voltage scaling V_DD_	scaling V_DD_ ⇒ scaling P_Switching_ quadratically	scaling V_DD_ ⇒ lower circuit speed (decreasing circuit performance)
Frequency Scaling f_SW_	scaling f_Clock_ ⇒ scaling P_Switching_ linearly	scaling f_Clock_ ⇒ lower circuit speed (decreasing circuit performance)
Minimization of switched capacitance C_L_ (small transistors, short wires, smaller fan-out)	Scaling C ⇒ scaling P_Switching_ and heat dissipation;	Potential degradation of signal quality; limited flexibility for system modifications or upgrades
Switching activity α	Lower switching activity α ⇒ more energy-efficient; lower switching activity α ⇒ reduced Electromagnetic Interference (EMI)	Extremely low switching activity α ⇒ increased propagation delays; extremely low switching activity α ⇒ instability and issues like signal crosstalk in the circuit; optimizing switching activity α ⇒ careful design considerations

**Table 5:** Comparison of Bluetooth versions.

Specifications	Bluetooth Classic BR/EDR ^1^	BLE	
		Bluetooth 4.x	Bluetooth 5
Radio freq. (MHz)	2400 to 2483.5	2400 to 2483.5	2400 to 2483.5
Channels	79 (1 MHz)	40 (2 MHz)	40 (2 MHz)
Distance (m)	Up to 100	Up to 100	Up to 200
Latency (ms)	100	<6	<6
Data rate (Mbps)	1, 2, 3	1	0.5, 0.125, 1, 2
Max active nodes	8	Unlimited	Unlimited
Massage size (bytes)	Up to 358	31	255
Max payload (bytes)	1021	37,255	255
Peak current (mA)	<30	<15	<15

^1^ Bluetooth Basic Rate/Enhanced Data Rate (BR/EDR).

**Table 6:** The evolution of Wi-Fi technology and standards.

Amendment	Naming Convention	Year	Operating Band	Max Bandwidth	Max Data Rate	PHY	Low Latency	Low Power
802.11b	Wi-Fi 1	1999	5 GHz	22 MHz	11 Mbps	DSSS	yes	yes
802.11a	Wi-Fi 2	1999	2.4 GHz	20 MHz	54 Mbps	OFDM	yes	yes
802.11g	Wi-Fi 3	2003	2.4 GHz	20 MHz	54 Mbps	MIMO-OFDM	yes	yes
802.11n	Wi-Fi 4	2008	2.4/5 GHz	40 MHz	600 Mbps	OFDM	yes	yes
802.11ac	Wi-Fi 5	2014	5 GHz	40 MHz	6.39 Gbps	256-QAM, OFDM, DL MIMO, channel bounding	yes	yes
802.11ah	Wi-Fi HaLow	2017	Sub-1 GHz	16 MHz	347 Mbps	OFDM, DL-MU MIMO	yes	yes
802.11ax	Wi-Fi 6	2019 2020 (6E)	2.4/5 GHz, 6 GHz for Wi-Fi 6E	160 MHz	9.6 Gbps	OFDMA, UL/DL MIMO, Channel Bounding	yes	yes
802.11be	Wi-Fi 7	2024	2.4/5/6 GHz	20 MHz	40 Gbps	4096-QAM, Coordinated OFDMA, UL/DL MIMO	yes	yes

**Table 7:** ZigBee technology parameters.

Parameters	ZigBee
Standard	IEEE 802.15.4
Frequency band	868/915 MHz and 2.4 GHz
Modulation type	BPSK/OQPSK
Spreading	DSSS
Number of RF channels	1, 10, and 16
Channel bandwidth	2 MHz
Power consumption in TX mode	Low (36.9 mW)
Data rate	20, 40, and 250 kbps
Latency	(20–30) ms
Communication range	100 m
Network size	65,000
Cost	Low
Security capability	128 bits AES
Network Topologies	P2P, tree, star, mesh
Application	WPANs, WSNs, and Agriculture
Limitations	line-of-sight (LOS) between the sensor node and the coordinator node must be available

**Table 8:** Z-Wave technology parameters.

Parameters	Z-Wave
Standard	ITU-T G.9959 (PHY and MAC)
RF Frequency Range	868.42 MHz in Europe, 908.42 MHz in US
Data rate	9.6, 40, 100 Kbps
Maximum Nodes	232
Architecture	Master and slave in mesh mode
MAC layer	CSMA/CA
RF PHY modulation	FSK (for 9.6 kbps and 40 kbps), GFSK with BT = 0.6 (for 100 kbps)
Coding	Manchester (for 9.6 kbps), NRZ (for 40 and 100 kbps)
Distance	30 m in indoors, 100 m the outdoors

**Table 9:** IPv6 over Low-Power Wireless Personal Area Networks (6LoWPAN) technology parameters.

Standard	Network	Topology	Power	Frequency Bands	Data Rate	Range	Spreading	Security	Common Applications
IEEE 802.15.4	WPAN	Star, Mesh	Low	868 MHz (EU), 915 MHz (USA), 2.4 GHz (Global)	250 kbps	10–100 m Short Range	DSSS	AES-128	Monitor and Control via Internet

**Table 10:** LoRaWAN technology parameters.

Coverage	Payload	Data Rate (Max)	Frequency Range	Security	Transmission Power
15 Km	243 Bytes	<50 kbps	125 kHz	AES Encryption	20 dBm

**Table 11:** SigFox technology parameters.

Coverage	Payload	Data Rate (Max)	Frequency Range	Security	Transmission Power
13 Km	12 Bytes	<100 kbps	868/915 MHz	None	13.5 dBm

**Table 12:** Narrowband Internet of Things (NB-IoT) technology parameters.

Modulation	Band	Data Rate	Range	MAC	Topology	Payload Size	Proprietary Aspects	Deployment Model
QPSK	Licensed 700–900 MHz	158.5 kbps (UL) ^1^, 106 kbps (DL) ^2^	15 km	FDMA/OFDMA	Star	125 B (UL), 85 B (DL)	Full stack	Operator-based

^1^ Uplink (UP); ^2^ Downlink (DL).

**Table 13:** 4G and 4G LTE technology parameters.

Technology	Frequency Band	Range	Maximum Data Rate	Channel Bandwidth	Modulation	Scalability	Reliability	Low Latency	Low Power
LTE-M (Rel13)	1.7–2.1 GHz, 1.9 GHz, 2.5–2.7 GHz	12 km	1 Mbps	1.4 MHz	BPSK/QPSK	Yes	Yes	Yes	Yes
LTE-M (Rel14)	1.7–2.1 GHz, 1.9 GHz, 2.5–2.7 GHz	12 km	4 Mbps	5 MHz	BPSK/QPSK	Yes	Yes	Yes	Yes

**Table 14:** Comparison of recent generations of mobile networks.

	2G	3G	4G	5G
Year of Introduction	1993	2001	2009	2018
Technology	GSM	WCDMA	LTE, WiMAX	MIMO, mmWaves
Access System	TDMA, CDMA	CDMA	CDMA	OFDM, BDMA
Switching Type	Circuit, packet	Circuit, packet	Packet	Packet
Network	PSTN	PSTN	Packet Network	Internet
Internet Service	Narrowband	Broadband	Ultrabroadband	Wireless World Wide Web
Bandwidth	25 MHz	25 MHz	150 MHz	700 MHz (Europe)
Speed	64 Kbps	8 Mbps	300 Mbps	10–30 Gbps
Latency	300–1000 ms	100–500 ms	20–30 ms	1–10 ms
Mobility	60 km	100 km	200 km	500 km

**Table 15:** A comparative analysis of 6G, 4G, and 5G mobile communication systems.

Parameters	4G	5G	6G
Peak data rate/device	1 Gbps	10 Gbps	1 Tbps
Latency	100 ms	1 ms	0.1 ms
Max. spectral efficiency	15 bps/Hz	30 bps/Hz	100 bps/Hz
Energy efficiency	<1000× relative to 5G	1000× relative to 4G	>10× relative to 5G
Connection density	2000 devices/km^2^	1 million devices/km^2^	>10 million devices/km^2^
Coverage percent	<70%	80%	>99%
Positioning precision	Meters precision (50 m)	Meters precision (20 m)	Centimeter precision
End-to-end reliability	99.9%	99.999%	99.9999%
Receiver sensitivity	Around −100 dBm	Around −120 dBm	<−130 dBm
Mobility support	350 km/h	500 km/h	>1000 km/h
Satellite integration	No	No	Fully
AI	No	Partial	Fully
Autonomous vehicle	No	Partial	Fully
Extended Reality	No	Partial	Fully
Haptic Communication	No	Partial	Fully
THz communication	No	Limited	Widely
Service level	Video	VR, AR	Tactile
Architecture	MIMO	Massive MIMO	Intelligent surface
Max. frequency	6 GHz	90 GHz	10 THz

**Table 16:** Essential parameters of satellite constellations: a comprehensive overview [57].

Parameter	GEO	LEO
Altitude	36,000 km	500 to 1200 km
Coverage area	Vast	Narrow
Downlink and uplink rate (signal speed)	Slow	Fast
Ground station spacing	Distant	Local
Antenna	Stationary	Complex tracking and terrestrial network
Band		
Frequency	band (4–8 GHz), Ku-band (12–18 GHz), and Ka-band (26.5–40 GHz)	L-band (1–2 GHz)
Capacity uplink	10–50 Mbps	100 Mbps for traditional LEO; 1 Gbps for advanced LEO
Capacity downlink	100–500 Mbps	500 Mbps for traditional LEO; 10 Gbps for advanced LEO
Latency	550 ms	25–50 ms
Advantages	Global coverage for communication and monitoring; High data transmission rates for high-speed internet; Stable and continuous coverage over a specific geographic area; Used for telecommunication, weather forecasting, navigation, and military surveillance; Easily accessible signals for remote and rural areas with small ground antennas.	Lower latency and faster communication speeds compared to GEO satellites; Coverage in areas without traditional communication networks; Easy deployment and repositioning for adaptation to changing needs; Cost-effective construction and launch compared to GEO satellites.
Disadvantages	Building and launching GEO satellites is expensive; The high orbit altitude of GEO satellites causes signal delays, affecting real-time communication services; GEO satellites can be affected by signal interference, like jamming and solar flares, which disrupt communication services; GEO satellites have limited bandwidth capacity, which can be a bottleneck for high-demand services; GEO satellites are limited in coverage due to fixed orbital positions.	LEO satellites have a shorter lifespan, lasting around 5–7 years; LEO satellites have limited bandwidth compared to geostationary satellites, leading to slower speeds and lower data capacity; LEO satellites require more frequent maintenance and repositioning, increasing operational costs; LEO satellites need constant signal handoffs as they move, causing potential interruptions and delays; LEO satellites have less coverage than geostationary satellites, potentially necessitating a higher quantity of satellites for complete global coverage.

**Table 17:** Specific functions of a PMU within a wireless sensor node.

Functions	Descriptions
Monitoring and managing the battery level	The PMU monitors the battery level of the node to maintain optimal power supply
Power gating	The PMU controls power supply to node components, turning them on or off to save power
Sleep modes	PMU can optimize power usage by putting the node into low-power sleep modes when not in use, thus conserving battery life.
Voltage regulation	PMU regulates node component voltage to ensure optimal operation and reduce power wastage
Power optimization	PMU uses power-saving algorithms and techniques like duty cycling and voltage scaling to optimize node power consumption.

**Table 18:** Comparison between LTC3108, LTC3105.

DC-DC Converter	Minimum V_IN_	Maximum V_IN_	Vout	MPPC/MPPT
LTC3108	20 mV	500 mV	2.35 V to 5 V	No
LTC3105	250 mV (start-up mode) 225 mV (regime mode)	5 V	3.3 V	Yes

**Table 19:** Example of energy sources available in the environment.

Energy Source Types	Description
Uncontrolled but predictable	Although unpredictable, renewable energy can be accurately planned out, allowing us to anticipate its availability within a certain margin of error.
Uncontrolled and unpredictable	Unpredictable and inconsistent, this energy source is difficult to regulate or manage due to its natural variability and intermittent availability.
Fully controllable	Energy can be generated when desired
Partially controllable	This energy source shows some level of control over the output, but is not fully controllable or predictable.

**Table 20:** Characteristics of some energy sources.

Energy Source	Predictable	Unpredictable	Controllable	Non-Controllable
Solar	✓			✓
RF	✓		✓	
Thermal		✓	✓	
Pyroelectric		✓	✓	

**Table 21:** Detailed comparison of rechargeable and non-rechargeable batteries’ technology and characteristics.

Type	Rated Voltage (V)	Capacity (Ah)	Temperature Range (°C)	Cycling Capacity	Specific Energy (Wh/kg)
Lead-Acid	2	1.3	−20–60	500–1000	30–50
MnO_2_Li	3	0.03–5	−20–60	1000–2000	280
Li poly-carbon	3	0.025–5	−20–60	-	100–250
LiSOCl_2_	3.6	0.025–40	−40–85	-	350
LiO_2_S	3	0.025–40	−60–85	-	500–700
NiCd	1.2	1.1	−40–70	10,000–20,000	50–60
NiMH	1.2	2.5	−20–40	1000–20,000	60–70
Li-Ion	3.6	0.74	−30–45	1000–100,000	75–200
MnO_2_	1.65	0.617	−20–60	-	300–610

**Table 22:** The latest innovations in the field of battery technologies.

Battery Generation	Technology/Electrode Active Materials	Cell Chemistry/Type	Implementation Date/Forecast Market Deployment
Gen 1	Cathode: NFP, NCA, LCO ^1^ Anode: Carbone/Graphite	Lithium-Ion	1991
Gen 2a	Cathode: NMC111, LMO_2_^2^ Anode: Carbone/Graphite	Lithium-Ion	1994
Gen 2b	Cathode: NMC532, NMC622 ^3^ Anode: Carbone/Graphite	Lithium-Ion	2005
Gen 3a	Cathode: NMC622, NMC 811 ^4^ Anode: Graphite + 5/10% Si	Lithium-Ion	2020
Gen 3b	Cathode: High Energy NMC, High Voltage Spinel—5 V Anode: Silicon/Carbon	Optimized Lithium-Ion	2025
Gen 4a	Cathode: NMC Anode: Silicon/Carbon Solid Electrolyte	Solid State Lithium-Ion	2025
Gen 4b	Cathode: NMC Anode: Lithium metal Solid Electrolyte	Solid State Lithium-Metal	>2025
Gen 4c	Cathode: High Energy NMC, High Voltage Spinel Anode: Lithium metal Solid Electrolyte	Advanced Solid State	2030
Gen 5	LiO2 Li–Air/Metal–Air Li-Sulphur New ion-based systems (Na, Mg, Zn or Al)	Metal–Air	>2030
		LiS	
		New ion-based insertion chemistries	

^1^ NFP (Lithium Nickel Fluorophosphate), a promising cathode material for lithium-ion batteries, which has high energy density and stability. NCA (Lithium Nickel Cobalt Aluminum Oxide) is known for its high energy density and thermal stability. LCO (Lithium Cobalt Oxide), known for its energy density and power output; ^2^ NMC111 and LMO_2_ are both cathode materials for lithium-ion batteries. NMC111 (nickel manganese cobalt oxide), with a balanced mix of nickel, manganese, and cobalt, offers a good combination of energy density, power density, and cycle life. It is commonly used in high-performance batteries. On the other hand, LMO_2_ (lithium manganese oxide) is known for its stability, safety, and cost-effectiveness. ^3^ NMC532 refers to a cathode material composed of 5 parts nickel, 3 parts manganese and 2 parts cobalt to make NMC532, which may have slightly lower thermal stability. ^4^ NMC 811 (Nickel Manganese Cobalt) provides a good balance of energy density, power density, and thermal stability. NMC622 offers higher energy density.

**Table 23:** Comparison between batteries and capacitors based on temperature sensitivity, charging/discharging speed, and voltage output.

Capacitor	Battery
Electric field for storage	The chemical reaction for storage
Submissive component	Active component
Energy density is low	Energy density is high
Charging/discharging is fast	Charging/discharging is slow
Provides unstable voltage	Provides constant voltage
Operating temperature range is −3 °C to +125 °C	20 °C to 30 °C during charging and 15 °C to 25 °C during discharging
Higher cost	Low cost
Contrived of metal sheets	Contrived of metals, chemicals

**Table 24:** Basic parameters of supercapacitors.

Supercapacitor	Life Cycle (-)	Specific Energy (Wh/kg)	Operating Temperature (°C)	Cell Voltage (V)
Maxwell PC10	500,000	1.4	−40–70	2.50
Maxwell BCAP0350	500,000	5.1	−40–70	2.50
Green-cap EDLC	>100,000	1.47	−40–60	2.70
EDLC SC	1,000,000	3–5	−40–65	2.70
Pseudo SC	100,000	10	−40–65	2.3–2.8
Hybrid SC	500,000	180	−40–65	2.3–2.8

**Table 25:** Comparison of batteries, fuel cells, capacitors and supercapacitors.

Property	Batteries	Fuel Cells	Capacitors	Supercapacitors
Weight	1 g–*>*10 kg	20 g–*>*5 kg	1 g–10 g	1 g–230 g
Operating temperature	−20 to 65 °C	25 to 90 °C	−20 to 100 °C	−40 to 85 °C
Operating voltage	1.25–4.2 V	0.6 V	6–800 V	2.3–2.7 V
Power density	0.005–0.4 KW/Kg	0.001–0.1 KW/Kg	0.25–10.0 KW/Kg	10–120 KW/Kg
Energy density	8 to 600 Wh/Kg	300 to 3 Wh/Kg	0.01 to 0.05 Wh/Kg	1–10 Wh/Kg
Pulse load	~5 A	~150 mA/cm2	~1000 A	~100 A
Life cycle	50,000 h + Unlimited Cycles	>100,000 cycles	1500–10,000 h	150–1500 cycles
Capacitance	-	-	10 pF–2.2 mF	100 mF–1500 F
Charge/discharge time	1–10 h	10–300 h	Picoseconds—milliseconds	Millisecond—seconds
Columbic efficiency	70–85%	-	About 100%	Up to 99%
Charge method	Current and voltage	-	The voltage across the terminal, i.e., from a battery	The voltage across the terminal, i.e., from a battery

**Table 26:** Technical comparison of various energy storage technologies.

Energy Storage Type	Energy Density (Wh/kg)	Advantages	Disadvantages	References
Lead acid	25–50	Technologically matured; Low material cost; No memory effect; Low self-discharge rate; Investment costs are relatively modest; Ease of maintenance.	Short cycle life; Low energy/power density; Significant charging time; Environmentally unfriendly; Poor low-temperature properties; Not as reliable.	[81]
NiCd	40–75	Long life; Recyclable; Wide temperature range.	Disposal issues due to cadmium toxicity; High memory effect; High cost.	[82]
NiMH	70–100	Environmentally friendly; Long life cycle; High operating temperature range.	Service life reduces if deeply discharged; Very expensive; Memory effect; Poor recycling.	[83]
Li-Ion	150–350	Nearly 100% round-trip energy storage efficiency; Very high energy/power density; Relatively fast charging; Long cycle life; Highly reliable; Low self-discharge; No memory effect; Lightweight.	High investment costs; Complicated charge management; Safety issues due to thermal runaway; Susceptible to damage at high voltages.	[84]
Capacitors	0.01–0.05	High power density; No charging circuit needed.	Low energy density; Voltage proportional to the stored energy.	[85]
Supercapacitors	2–5	Wide working temperature range (−40–60 °C); Quick charging time; High charge/discharge cycle efficiency; Eco-friendly; High number of charge and discharge cycles, virtually an unlimited number of times.	Very high self-discharge rate; High cost; Requires complex electronic control; Low cell voltage; Voltage varies with the energy stored; Low energy density; Higher leakage current; Large size.	[86]

**Table 27:** Performance of supercapacitors with various configurations, based on data extracted from articles published between 2020 and 2023.

Device Configuration	Electrolyte	Electrode Type	Energy Density (Wh/kg)	Power Density (W/kg)	Publication Year	Reference
WO_3_-WS_2_-MWCNT/Ni foam// AC/Ni foam	3 M KOH	(+) PC//EDLC (−)	86 24	848 11,828	2023	[87]
Ni-Co-Mg MOF/MoS_2_/Ni foam// AC/Ni foam	1 M KOH	(+) PC//EDLC (−)	107.32	1350	2023	[88]
NH_4_MnPO_4_@Graphene QD/Graphite//rGO/Graphite	3 M H_2_SO_4_ 3 M H_2_SO_4_ + 0.025 M (KI/VOSO_4_)	(+) PC//EDLC (−)	199 311	450 450	2022	[89]
Ni_3_(PO_4_)_2_-MWCNTs/Ni foam// AC/Ni foam		(+) PC//EDLC (−)	94.4 24.82	340 10,200	2022	[90]
Mn-V-Sn oxyhydroxide/Ni foam// N-carbon/Ni foam	1 M KOH	(+) PC//EDLC (−)	70.6 17.1	1372.4 18,861.3	2022	[91]
NH4OH-ZIF/Ni foam//GO/Ni foam	6 M KOH	(+) PC//EDLC (−)	4.16	20,000	2022	[92]
CoS-Co_3_(PO_4_)_2_/Ni foam//AC/Ni foam	1 M KOH	(+) PC//EDLC (−)	34.68 63.93	13,600 850	2021	[93]
Fe_3_O_4_@N-carbon-rGO/Ni foam// rGO/Ni foam	6 M KOH	(+) PC//EDLC (−)	46 10	750 7500	2021	[94]
MWCNT-NiMnPO_4_/Ni foam// AC/Ni foam	2 M KOH	(+) PC//EDLC (−)	698 43	78 5780	2020	[95]
graphitic carbon nitride (g-C_3_N_4_)-BiVO_4_/Graphite paper (symmetric)	3.5 M KOH	(+) PC//PC (−)	61 7.2	1996 16,200	2020	[96]
Zn-Carbon cloths//S/P doped carbon (S/p-C)/graphite rod	0.5 M K_2_SO_4_ 1 M KBr	(+) PC//PC (−)	270 181	185 9300	2020	[97,98]

EDLC → electrical double-layer capacitive type; PC → pseudo capacitive type.

**Table 28:** Advantages and disadvantages of metal–air batteries (MAB).

Advantages	Disadvantages
High energy density	Limited cycle life
Long range	Slow recharge rate
Low cost	Limited power output
Rechargeable	Corrosion
Environmentally friendly	Limited availability

**Table 29:** Recent advances in six types of high energy density MABs.

Metal Oxygen	Open Circuit Potential (Volts)	Energy Density (Wh/kg)	Energy Density (Wh/L)
Al–air	2.71	4116	14,100
Ca–air	3.10	2980	9960
Mg–air	3.08	3991	12,200
Fe–air	1.35	763	1431
Li–air	2.96	3458	6102
Zn–air	1.68	1054	5960

**Table 30:** The latest advancements in advanced materials utilized for TFB technology, including current collectors, electrodes, and electrolyte materials.

Current Collectors	Electrode	Electrolyte	Voltage Window	Reference
Au	Cathode	1 M LiClO_4_ in PC ^1^ 1 M LiPF_6_ in EC/DMC ^2^	3–5 3–4.4	[104]
Ag	Cathode	1 M LiClO_4_ in PC 1 M LiPF_6_ in EC/DMC	3–3.7	[105]
Al	Cathode	1 M LiClO_4_ in PC 1 M LiPF_6_ in EC/DMC	1.5–5.5 1.5–5	[106]
Ni	Cathode	1 M LiPF_6_ in EC/DMC	3–4.5	[107]
Stainless steel	Cathode	1 M LiPF_6_ in EC/DMC	3–4.5	[108]
Stainless steel	Cathode	1 M LiPF_6_ in EC/DMC	1.5–5.5	[109]
Cr	Cathode/anode	1 M LiPF_6_ in EC/DMC	0–4	[110]
Ti	Cathode/anode	1 M LiPF_6_ in EC/DMC	0–4	[111]
TiN	Cathode/anode	1 M LiPF_6_ in EC/DMC	0–4.12	[112]
Carbon fiber paper	Cathode/anode	1 M LiPF_6_ in EC/DMC	1.5–3	[113]
Stainless steel	Cathode/anode	1 M LiPF_6_ in EC/DMC	2–3.4	[114]
Fe	anode	1 M LiPF_6_ in EC/DMC	0–3.2	[115]
Cu	anode	1 M LiPF_6_ in EC/DMC	0–3	[116]

^1^ Lithium perchlorate (LiClO_4_) in propylene carbonate (PC) is a common electrolyte solution able to improve the conductivity and stability of the battery; ^2^ A 1 M LiPF6 in EC/DMC solution contains lithium hexafluorophosphate dissolved in ethylene carbonate (EC) and dimethyl carbonate (DMC), commonly used as an electrolyte in lithium-ion batteries for ion movement between cathode and anode during charge and discharge.

**Table 31:** Compare the power densities of different sources and discuss their respective advantages and disadvantages.

Energy Source	Technology	Power Density	Advantages	Disadvantages	Application Domain
Solar [114]	PV cell 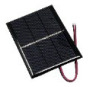	10–100 mW/cm^2^ (outdoor) <100 μW/cm^2^ (indoor)	High output voltage Low fabrication costs Predictable	Unavailable at night Non-controllable	Environment monitoring, healthcare, agriculture
RF [115]	Rectenna 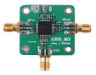	0.01–0.1 μW/cm^2^ 1–10 mW/cm^2^	Available anywhere, anytime Predictable Controllable	Distance dependent Low power density Interference	Environment monitoring
MID-IR [15]	Rectenna 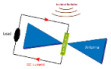	60 mW/cm^2^	Sustainable and reliable Available Controllable	Low power density Low efficiency	Environment monitoring, healthcare,

**Table 32:** Key parameters and their descriptions.

Full Name	Designation	Description
Efficiency or power conversion efficiency (PCE)	ƞ	It indicates how much energy can be extracted from sunlight by a single P–N junction
Open-Circuit Voltage	Voc	It indicates the maximum voltage that a solar cell can generate under illumination
Short-Circuit Current	Isc	It represents the maximum current that a solar cell can produce under full sunlight exposure
Fill Factor	FF	FF represents how efficiently a solar cell converts sunlight into electricity
Maximum Power Point	Pmax	Pmax represents the maximum electrical power output that a solar cell can generate
Voltage at Maximum Power	Vmpp	The voltage at which the solar cell operates to produce the maximum power output.
Current at Maximum Power	Impp	The current at which the solar cell operates to produce the maximum power output.
Shunt Resistance	Rsh	A higher Rsh value signifies reduced leakage current and enhanced efficiency.
Series Resistance	Rs	Lower Rs values lead to better performance.

**Table 33:** Overview of the materials commonly utilized in SCs, highlighting their respective benefits, limitations, and comparative efficiencies.

Material	Sub-Material	ƞ	Advantages	Problems
	Single crystal	20% [119]	(1) Monocrystalline solar cell is generally used due to its high-efficiency level as compared to the multi-crystalline cell [120]. (2) Occupies a small space [121].	(1) (c-Si) technology is too expensive for large-scale operation [122]. (2) (c-Si) modules have a weak interaction with light, as Si is an indirect bandgap semiconductor [120]. (3) Any form of dust or moisture on the Si solar module would break the whole circuit or cause a dramatic loss in power unless an effective cleaning technique is developed to clean the solar panels [117,123,124,125].
c-Si	Polycrystal	16% [126]	Cost-effective as compared to the monocrystalline module [120].	(1) Less efficiency than the monocrystalline module as it is made of impure Si [126]. (2) Occupies a larger area [121].
	a-Si	11.3% [127]	(1) Much cheaper than (Si-c) technology [127]. (2) Produced in a range of sizes and shapes [120].	(1) Lower efficiency than the mono-crystalline and poly-crystalline solar cells [128]. (2) Long-term instability [129].
	CdTe/CdS	18.3% [130]	(1) Good match with the solar spectrum to obtain energy at shorter wavelengths compared to Si modules [130]. (2) Cadmium is abundant [131].	(1) Approximately less efficiency than the c-Si solar cell [129]. (2) Te is an extremely rare element [130]. (3) Cd is toxic [131].
Thin Films	CIS/CIGS	22.8% [132]	(1) CIGS have a high efficiency that is similar to that of (c-Si) PV cells. (2) Less expensive, as CIGS can absorb light using only ~2.0–2.5 mm layer thickness, which decreases the use of raw materials. (3) Easy to fabricate compared to (c Si)-based PV cells [133].	In and Ga sources are limited [134].
	GaAs	Over 30% [135]	(1) High resistivity to heat damage [136]. (2) High absorption coefficient, as GaAs is a direct bandgap material [137]. (3) The highest PCE [135].	(1) Ga is an expensive material because of its low abundance [138]. (2) As is highly toxic [139].
Organic Semiconductors		18%	Flexibility and versatility; Low-cost production; Tunable properties; Large-area fabrication	Low carrier mobility; Sensitivity to environmental factors; Limited device lifetime; Narrow operational temperature range; Limited energy levels and bandgaps
Quantum Dots		20%	Size-tunable properties; High quantum yield; Broad absorption spectra; Good stability; Compatibility with different substrates	Toxicity concerns; Cost; Limited device lifetime; Difficulty in large-area deposition; Complexity
Quantum Wells		40–50% (only in laboratory)	Bandgap engineering; Improved carrier confinement; Efficient light emission; Compatibility with existing semiconductor technologies; High quantum efficiency	Complexity of fabrication; Strain-related issues: Temperature sensitivity Narrowband emission Sensitivity to defects
Perovskite		24%	High light absorption; Tunable bandgap; Solution processability; High charge carrier mobility; Versatility; High efficiency	Environmental stability; Toxic materials; Performance and reproducibility; Limited device lifetime; Scalability issues

**Table 34:** Main characteristics of SCs based on Si [119].

Material Structure	S, cm^2^	Jsc, mA/cm^2^	FF, %	Efficiency
c-Si	4.00	40.9	82.7	24.0
c-Si	45.7	39.4	78.1	21.6
c-Si	22.1	41.6	80.3	23.4
mc-Si	1.00	36.5	80.4	18.6
mc-Si	100	36.4	77.7	17.2
tf-Si	240	27.4	76.5	12.2
tf-Si	4.04	379	81.1	21.1
a-Si:H	1.06	16.66	71.7	10.3
a-Si:H	0.99	17.46	70.4	10.9
a-Si:H	1.0	19.4	74.1	12.7
a-Si:H	1.08	18.8	70.1	1 1.5
ITO/c-Si/a-Si	1.0	39.4	79.0	20.0
a-Si:H	1.0	19.13	70.0	12.0
a-Si:H	1.0	18.4	72.5	12.3
a-Si/a-Si/a-SiGe		7.3	73.0	12.4
a-Si:H	1.0	19.4	74.1	12.7
a-C/a-SiML/a-SiC/a-Si	1.0	19.6	71.8	13.2
a-C/a-Si/a-SiC/a-Si	1.0	19.8	73.3	13.2
ITO/a-Si:H/a-SiGe:H	0.28	11.72	65.8	12.5
a-Si/k-Si	0.03	16.2	63.0	15.0
a-SiC/a-Si	1.0	8.16	71.2	10.2
a-Si/a-Si	1.0	9.03	74. I	12.0
a-SiC/a-SiGe/a-SiGe	1.0	7.9	68.5	12.4
a-Si/a-Si/a-siGe	1.0	7.66	70.1	13.7
p-a-SiO:H/a-Si:H/n-a-Si:H	1.0	18.8	74.0	12.5
ITO/a-Si:H/Si:H/a-siGe	0.27	6.96	70	12.4
a-Si:H/a-Si:H/a-SiGc:H	1.00	7.9	68.5	12.4
a-Si/CuInSe2		16.4 17.4	72.0 68.0	10.3 5.3
a-Si/mc-Si		10.4 30.2	76.0 79.2	7.25 13.75

**Table 35:** Main characteristics of SCs based on CdTe [140,141,142,143].

Material Structure	S, cm^2^	Jsc, mA/cm^2^	FF%	Efficiency%
ss/ITO/CdS/CdTc//Cu/Au	0.191	20.10	69.4	11.0
ss/SnO_2_/CdS/CdTe	0.824	20.66	74.0	12.8
ss/SnO_2_/CdS/CdTe	0.313	24.98	62.7	12.3
ss/SnO_2_/CdS/CdTe	0.3	26.18	61.4	12.7
ss/SnO_2_/CdS/HgTeGa	1.022	21.9	65.7	10.6
MgF_2_/ss/SnO_2_/CdS//CdTe/C/Ag	1.047	25.09	74.5	15.8
ss/SnO_2_/CdS/CdTe/Ni	1.068	20.93	69.6	11.2
ss/SnO_2_/CdS/CdTe	0.08	22.1	66.0	10.9
MgF2/ss/SnO_2_/CdS/CdTe	1.115	20.9	74.6	12.9
Ss/SnO2/CdS/CdTe/Cu/Au	0.114	17.61	72.8	10.4
CdTe				12.7

**Table 36:** Performance attained by various configurations of Perovskite Solar Cells (PSC) with progressive years.

PCE (%)	Voc (V)	Jsc (mA/cm^2^)	FF	Device Configuration	Year	Reference
7.11	0.63	18.61	0.606	FTO/PCBM/CsSn_0.5_Ge_0.5_I_3_/Spiro-OMeTAD/Au	2019	[146]
7.37	0.73	15.8	0.64	Au/TiO_2_/m-TiO_2_/MASn_0.25_Pb0._75_/Spiro-OMeTAD/Au	2014	[147]
7.66	0.97	11.1	7.66	ITO/ZnO/MASnI_3_/spiro-OMeTAD/Au	2015	[148]
9	0.52	24.1	0.71	ITO/PEDOT:PSS/FASnI_3_/C_60_BCP/Ag	2017	[149]
9.2	0.61	21.2	0.72	ITO/PEDOT:PSS/GA_x_FA_0.98*−*x_SnI_3_–1% EDAI_2_/C_60_ (20 nm)/BCP/Ag	2018	[150]
9.8	0.76	19.1	0.66	ITO/PEDOT:PSS/MAPb_0.85_Sn_0.15_I_3*−*y_Cl_y_/PC_61_BM/Ag	2014	[151]
10.2	0.72	19.2	0.73	FTO/TiO_2_/N719 Dye/Perovskite/ZnO	2012	[152]
12.1	0.78	20.65	0.75	ITO/PEDOT: PSS/MASn_0.6_Pb_0.4_I_3*−*x_Br_x_/PCBM/Ag	2017	[153]
13.24	0.84	20.32	0.78	FTO/PEDOT PSS/EA_0.98_EDA_0.01_SnI_3_/C_60_BCP/Au	2020	[154]
14.06	0.79	22.8	0.78	ITO/PEDOTPSS/MA_0.5_FA_0.5_Pb_0.75_Sn_0.25_I_3_/PC_61_ BM/C_60_/Ag	2016	[155]
10.2	0.7	21.9	0.66	ITO/PEDOT:PSS/FASn0.5Pb0.5I3/C60 BCP/Ag	2016	[156]
14.1	0.74	26.1	0.71	ITO/PEDOT:PSS/C60 BCP/Ag	2016	[157]
15.08	0.79	26.86	0.70	ITO/PEDOT:PSS/(CH_3_NH_3_)_0.4_[HC(NH_2_)_2_]_0.6_Sn_0.6_Pb_0.4_I_3_/C_60_/BCP/Ag	2016	[158]
17.55	1.03	21.9	0.78	ITO/PEDOT:PSS/MAPb_0.85_In_0.15_I_3_Cl_0.15_/PC_61_ BM/Bphen/Ag	2016	[159]
18	1.02	22.4	0.78	FTO/SnO_2_/Cs_0.16_FA_0.84_Pb(I_0.88_Br_0.12_)_3_/Spiro-OMeTAD/Au		[160]
19.1	1.01	22.4	0.78	FTO/Poly-TPD/0.15 mol% Al_3_+-doped CH3NH3PbI3/PCBM/BCP/Ag	2016	[161]
22.3	1.71	24.1	0.81	ITO/PTAA/Cs_0.05_(FA_0.92_MA_0.08_)_0.95_Pb(I_0.92_Br _0.08_)_3_/C_60_/BCP/Cu	2020	[162]
23	1.16	24	0.82	Glass/ITO/PTAA/(Cs_0.05_(FA_5_/MAI)_0.95_Pb(I_0.9_Br_0.1_)_3_)/PCBM/BCP/Ag	2021	[163]
23.7	1.16	24.16	0.84	Glass/ITO/PTAA/PEAI/(Cs_0.05_(FA_5_/MAI)_0.95_Pb(I_0.9_Br_0.1_)_3_)/PEAI/PCBM/BCP/Ag	2021	[164]
24.6	1.05	25.5	0.83	FTO/SnO_2_/(FAPbI3)_0.95_(MAPbBr_3_)_0.05_/P3HT/Au	2023	[165]
24.8	1.16	26.35	0.8	FTO/c-TiO_2_/m-TiO_2_/FAPbI_3_/Spiro-OMeTAD/Au	2020	[166]
25.4	1.19	25.09	0.84	FTO/SnO_2_/MAPbBr_3_/HTL/back contact	2021	[167]
25.5	1.18	25.74	0.83	FTO/SnO_2_-Cl/FAPbI_3_/Spiro-OMETAD/Au	2021	[168]

**Table 37:** The power density of different RF bands.

Source	Conditions	Frequency	Power Density	Efficiency
	DTV	470–610 MHz	0.89 nW/cm^2^	±50%
	GSM (MT)	880–915 MHz	0.45 nW/cm^2^	±50%
	GSM/4G LTE 900 (BT)	920–960 MHz	36 nW/cm^2^	±50%
RF (Average) [170]	GSM/4G LTE 1800 (MT)	1710–1785 MHz	0.5 nW/cm^2^	±50%
	GSM 1800 (BT)	1805–1880 MHz	84 nW/cm^2^	±50%
	3G (MT)	1710–1785 MHz	0.46 nW/cm^2^	±50%
	3G (BT)	2110–2170 MHz	12 nW/cm^2^	±50%
	Wi-Fi	2.4–2.5 GHz	0.18 nW/cm^2^	±50%
	4G LTE 2600	2500–2690	0.3 mW/cm^2^–0.000767 mW/m^2^	±50%

**Table 38:** Comparative analysis of existing circuits for the RF-EHWSN.

Ref.	Frequency (GHz)	Max Conversion Efficiency (%)	Circuit Size (mm^3^)	Pin (dBm)	Max Gain (dBi)	Max Harvested DC Output Voltage (v)	Substrate	Distance (m)	Diode Type
[176]	24	80	40 × 40 × 1.6	4.9	7.8	6.82	FR-4	1.5	Schottky CMOS
[177]	2.45	20	24.9 × 8.6 × 1.6	−20	0.8	0.097	FR-4	0.9	HSMS-2852 Schottky
[178]	2.45	-	160 × 130 × 0.55	–40 to 0	5	1.05@1.5 m; 1@2 m	Cordura fabric	1.5 2	HSMS-2862 Schottky
[179]	3.1–8	69	6.3 × 13 × 0.8	−10	3.2	-	FR-4	0.5	SMS 7630
[180]	1.975–4.744	88.58	40 × 45 × 1.6	0	4.3	10.703	FR-4	2	HSMS 270B Schottky
[181]	0.91–2.55	68	165 × 165 × 0.8	−10	5 to 8.3	0.243	FR-4	-	HSMS-285C
[182]	1.7–3	60	178 × 148 × 0.813	-	9.902	3.7	Roger RO4003C	0.75	SMS7630
[183]	2.4	50	63.7 × 45.6 × 1.6	−10 to 17	5.3	3	FR-4	1–2.5	HSMS 2850 and SMS7630
[184]	2.1 and 3.3	76.3	31 × 18 × 1	4 to 16	-	-	F4B	-	HSMS286
[185]	2.4	69.3	4 × 11.7 × 1.6	5.2	5.9	3.5	RO4003C	-	SMS7630
[186]	2.45	19.5–44.6	150 × 80 × 4	−9.48	8.53	-	RO4003	-	SMS7630
[187]	2.45 and 3.6	59%@ 2.45; 41% @3.6	44 × 24.5 × 0.06	2	2.6@2.45; 1.6@3.6	-	Rogers R04003	0.65	SMS-7630
[188]	2.2	50	71 × 71 × 1.6	29	7.46	0.516 in parallel 1.087 in series	RT/duroid 5880 Rogers	1	SMS7621
[189]	0.909	88	99.5 × 26 × 0.508	−10	4.6	7	Rogers 5880	1.2	HSMS286C SMS7630
[190]	20–26.5	70	32.6 × 16 × 4	27	8	6.5	Textile	0.12	MA4E-1319
[191]	0.915–2.4	80	115 × 15 × 1.4	−7	2.3	1.8	Textile	4.2	BAT15-04R
[192]	0.83	63	-	−10	1.7	0.65	Felt	0.89	SMS7630-079lf

**Table 41:** Summary of recent achievements in the field of graphene-based geometric diodes for THz rectennas.

Diode Configuration	Nanoantenna	Operating Frequency (THz)	Maximum Responsivity (V^−1^)	Zero-Bias Responsivity (V^−1^)	Zero-Bias Resistance (Ω)
Exfoliated monolayer graphene-based arrowhead-shaped diode [234]	metal bowtie 15 nm Cr/40 nm Au	28.3	0.2 for V_DS_ = 1.5 V	0.18 for V_DS_ = 0 V	13 K
Exfoliated monolayer graphene-based arrowhead-shaped diode [235]	metal bowtie 15 nm Cr/40 nm Au	Up to 160	0.8 for V_DS_ = 0.4 V	0.3 for V_DS_ = 0 V	19 K
Exfoliated monolayer graphene-based arrowhead-shaped diode [236]	metal bowtie 15 nm Cr/40 nm Au	28.3	0.2 for V_DS_ (V) = 1.4 V	0.12 for V_DS_ = 0 V	3 K
(CVD) monolayer graphene-based arrowhead-shaped diode [237]	metal bowtie Ti (10 nm)/Au (40 nm)	28.3	0.3 for V_DS_ (V) = 0.5 V	0.1 for V_DS_ (V) = 0 V	5 K
Z-shaped graphene geometric diodes [233]	-	28.3	2.4 for V_0_ (V) = 0.5 V	-	-

## Data Availability

No new data were created or analyzed in this study. Data derived from public domain resources.

## Abbreviations

The following abbreviations are used in this manuscript:

ES	Energy Storage
ULPDT	Ultra-Low-Power Design Techniques
EHT	Energy Harvesting Technique
PMU	Power Management Unit
IoT	Internet of Things
ULP WCP	Ultra-Low Power Wireless Communication Protocol
WSN	Wireless Sensor Network
DC	Direct Current
DVS	Dynamic Voltage Scaling
DFS	Dynamic Frequency Scaling
RBB	Reverse Body Biasing
DT	Direct Tunneling
FNT	Fowler–Nordheim Tunneling
EMI	Electromagnetic Interference
ESP	Energy Saving Protocol
ITA NTP	Integrating Terrestrial and Non-Terrestrial Protocols
QAM	Quadrature amplitude modulation
UL/DL MIMO	Uplink/Downlink MIMO
OFDMA	Orthogonal frequency-division multiple access
BPSK/OQPSK	Binary Phase Shift Keying/Offset Quadrature Phase Shift Keying
WPANs	Wireless Personal Area Networks
6LoWPAN	IPv6 over Low-Power Wireless Personal Area Networks
LoRaWAN	Long Range Low Power Wide Area Network
CSS	Chirp Spread Spectrum
NB-IoT	Narrowband Internet of Things
LPWAN	Low Power Wide Area Network
GSM	Global System for Mobile Communications
WCDMA	Wideband Code Division Multiple Access
TDMA	Time Division Multiple Access
LTE	Long Term Evolution
GPIO	General-Purpose Input and Output Device
DAC	Digital-to-Analog Converter
MPPC/MPPT	Maximum Power Point Control/Tracking
MnO_2_Li	Manganese dioxide lithium
MnO_2_	Manganese Dioxide
LiSOCl_2_	Lithium Thionyl Chloride
LiO_2_S	Lithium sulfite
NiCd	Nickel-Cadmium
NiMH	Nickel-metal hydride
Li-Ion	Lithium-ion
EDLCs	Electric Double-Layer Capacitors
PCs	Pseudo Capacitors
HSCs	Hybrid Supercapacitors
MAB	Metal–air batteries
Li	Lithium
Na	Sodium
K	Potassium
ƞ	Efficiency
Voc	Open-Circuit Voltage
Isc	Short-Circuit Current
FF	Fill Factor
Pmax	Maximum Power Point
Vmpp	Voltage at Maximum Power
Impp	Current at Maximum Power
Rsh	Shunt Resistance
Rs	Series Resistance
c-Si	Single-crystalline silicon
mc-Si	microcrystalline Silicon
tf-Si	Thin-film silicon
a-Si	Amorphous silicon
MIG	Metal–insulator–graphene
IRA	Infrared-frequency Rectifying Antenna
IPA	Isopropyl Alcohol
SEM	Scanning Electron Microscope
GGD	Graphene-based geometric diodes
TEG	Thermoelectric energy generators
ML	Machine Learning
EHWSN	Energy Harvesting Wireless Sensor Network
EH	Energy Harvesting
TX	Transmitter
RX	Receiver
PMOS	Positive-Channel Metal-Oxide Semiconductor
NMOS	Negative-Channel Metal-Oxide Semiconductor
CMOS	Complementary Metal-Oxide-Semiconductor
DIBL	Drain-Induced Barrier Lowering
BLE	Bluetooth Low Energy
LE	Low Energy
BR/EDR	Bluetooth Basic Rate/Enhanced Data Rate
UWB	Ultra-Wideband
Wi-Fi	Wireless Fidelity
LANs	Local Area Networks
DSSS	Direct Sequence Spread Spectrum
MIMO-OFDM	Multiple-input, multiple-output orthogonal frequency-division multiplexing
LOS	Line-Of-Sight
CSMA/CA	Carrier Sense Multiple Access with Collision Avoidance
FSK	Frequency-Shift Keying
GFSK	Gaussian Frequency Shift Keying
RF	Radio Frequency
BDMA	Big Data Management and Analytics
PSTN	Public Switched Telephone Network
GEO	Geostationary Satellites Orbit
LEO	Low Earth Orbit
ENO	Energy-neutral operation
PNO	Power-neutral operation
IC	Intermittent computing
ULPMU	Ultra-low power management unit
IR	Infrared
ADC	Analog-to-Digital Converter
Mg	Magnesium
Al	Aluminum
Fe	Iron
Zn	Zinc
Al–air	Aluminum–air
Ca–air	Calcium–air
Mg–air	Magnesium–air
Fe–air	Iron–air
Li–air	Lithium–air
Zn–air	Zinc–air
TFBs	Thin-film batteries
SC	Solar Cell
PVE	Photovoltaic Effect
Eg	bandgap
AC	Alternating Current
MCU	Microcontroller Unit
EMW	Electromagnetic Wave
SPPs	Surface plasmon polaritons
LPF	Low-Pass Filter
MIM	Metal-insulator-metal
MI^n^M	Metal Multi-Insulator Metal, n represents the number of ultrathin insulator layers
FOM	Figures of Merit
Asym	Asymmetry
NL	nonlinearity
S	Responsivity
TOV	Turn-on Voltage
ZBR	Zero-Bias Resistance
ALD	Atomic Layer Deposition
S&Q	Shockley–Queisser
EBL	Electronic Beam Layer
MIBK	Methyl Isobutyl ketone
ANN	Artificial Neural Networks
HEH	Hybrid Energy Harvesting
RFID	Radio-Frequency Identification
UHF	Ultra-high frequency

## References

[1] Shaikh F.K., Zeadally S. (2016). Energy harvesting in wireless sensor networks: A comprehensive review. Renew. Sustain. Energy Rev..

[2] IRIS, Memsic, Inc. http://www.memsic.com/wireless-sensor-networks/XM2110CA.

[3] MicaZ, Memsic, Inc. http://www.memsic.com/wireless-sensor-networks/MPR2400CB.

[4] iMote2, Intel Research. http://tinyos.stanford.edu/tinyos-wiki/index.php/Imote2.

[5] SunSpot, Sunsystems http://www.sunspotworld.com/.

[6] Waspmote, Libelium Inc. http://www.libelium.com/products/waspmote/.

[7] WiSMote, Aragosystems http://www.aragosystems.com/en/wisnet-item/wisnet-wismote-item.html.

[8] Vullers R.J.M., van Schaijk R., Doms I., Van Hoof C., Mertens R. (2009). Micropower energy harvesting. Solid State Electron..

[9] Pecunia V., Silva S.R.P., Phillips J.D., Artegiani E., Romeo A., Shim H., Park J., Kim J.H., Yun J.S., Welch G.C. (2023). Roadmap on energy harvesting materials. J. Phys. Mater..

[10] Elahi H., Munir K., Eugeni M., Atek S., Gaudenzi P. (2020). Energy Harvesting towards Self-Powered IoT Devices. Energies.

[11] Russo J., Ray W., Litz M.S. (2017). Low light illumination study on commercially available homojunction photovoltaic cells. Appl. Energy.

[12] De Rossi F., Pontecorvo T., Brown T.M. (2015). Characterization of photovoltaic devices for indoor light harvesting and customization of flexible dye solar cells to deliver superior efficiency under artificial lighting. Appl. Energy.

[13] Visser H.J., Reniers A.C., Theeuwes J.A. Ambient RF energy scavenging GSM and WLAN power density measurements. Proceedings of the 2008 38th European Microwave Conference.

[14] Mazunga F., Nechibvute A. (2021). Ultra-low power techniques in energy harvesting wireless sensor networks: Recent advances and issues. Sci. Afr..

[15] Shanawani M., Masotti D., Costanzo A. (2017). THz Rectennas and Their Design Rules. Electronics.

[16] Harb A. (2011). Energy harvesting State-of-the-art. Renew. Energy.

[17] Sanislav T., Mois G.D., Zeadally S., Folea S.C. (2021). Energy Harvesting Techniques for Internet of Things (IoT). IEEE Access.

[18] Rawat S., Das S. (2017). A Review of Existing Techniques for Reducing Power Consumption in VLSI Circuits. Int. J. Emerg. Technol..

[19] Rabaey J., Pedram M. (1996). Low Power Design Methodologies.

[20] Chandrakasan A., Brodersen R. (1998). Low-Power CMOS Design.

[21] Chandrakasan A.P., Sheng S., Brodersen R.W. (1992). Low-Power CMOS Digital Design. IEEE J. Solid-State Circuits.

[22] Najm F. (1994). A Survey of Power Estimation Techniques in VLSI Circuits. IEEE Trans. VLSI Syst..

[23] Pedram M. (1994). Power Estimation and Optimization at the Logic Level. Int. J. High Speed Electron. Syst..

[24] Macii E., Pedram M., Somenzi F. (1998). High-Level Power Modeling, Estimation, and Optimization. IEEE Trans. Comput. Aided Des. Integr. Circuits Syst..

[25] Borkar S. (1999). Design Challenges of Technology Scaling. IEEE Micro.

[26] Thompson S., Packan P., Bohr M. (1998). MOS Scaling: Transistor Challenges for the 21st Century. Intel. Technol. J..

[27] Chen Z., Shott J., Plummer J. CMOS Technology Scaling for Low Voltage Low Power Applications. Proceedings of the ISLPE-98: IEEE International Symposium on Low Power Electronics.

[28] Ye Y., Borkar S., De V. A New Technique for Standby Leakage Reduction in High-Performance Circuits. Proceedings of the 1998 Symposium on VLSI Circuits.

[29] Pedram M. (1996). Power Minimization in IC Design: Principles and Applications. ACM Trans. Des. Autom. Electron. Syst..

[30] Chen B., Nedelchev I. Power Compiler: A Gate Level Power Optimization and Synthesis System. Proceedings of the ICCD’97: IEEE International Conference on Computer Design.

[31] Benini L., Bogliolo A., De Micheli G. (2000). A Survey of Design Techniques for System-Level Dynamic Power Management. IEEE Trans. VLSI Syst..

[32] Roy K., Mukhopadhyay S., Mahmoodi-Meimand H. (2003). Leakage Current Mechanisms and Leakage Reduction Techniques in Deep- Submicrometer CMOS Circuits. Proc. IEEE.

[33] Chandrakasan A.P., Brodersen R.W. (1995). Minimizing Power Consumption in Digital CMOS Circuits. Proc. IEEE.

[34] Gu R.X., Elmasry M.I. (1996). Power Dissipation Analysis and Optimization of Deep Submicron CMOS Digital Circuits. IEEE J. Solid-State Circuits.

[35] Wang A., Calhoun B., Chandrakasan A.P. (2006). Sub-Threshold Design for Ultra-Low-Power Systems.

[36] Butzen P.F., Ribas R.P. Leakage reduction technique for CMOS complex gates. Proceedings of the South Symposium on Microelectronics.

[37] Kim C.H., Roy K. Dynamic Vth Scaling Scheme for Active Leakage Power Reduction. Proceedings of the 2002 Design Automation and Test in Europe Conference.

[38] Rao R., Srivastava A., Blaauw D., Sylvester D. (2004). Statistical Analysis of Subthreshold Leakage Current for VLSI Circuits. IEEE Trans. Very Large Scale Integr. (VLSI) Syst..

[39] Lorenzo R., Chaudhury S. (2016). Review of Circuit Level Leakage Minimization Techniques in CMOS VLSI Circuits. IETE Tech. Rev..

[40] Tsang T.K., El-gamal N.M., Iniewski K., Townsend K.A., Haslett J.W., Wang Y. (2007). Current Status of CMOS Low Voltage and Low Power Wireless IC Designs. Analog. Integr. Circuits Signal Process..

[41] Cheng J.M., Pedram M. (1992). Energy Minimization using Multi Supply Voltage. IEEE Trans. VLSI Syst..

[42] Hanchate N., Ranganathan N. A New Technique for Leakage Reduction in CMOS Circuits using Self-Controlled Stacked Transistors. Proceedings of the 17th International Conference on VLSI Design (VLSID”04) IEEE.

[43] Sánchez-Álvarez D., Linaje M., Rodríguez-Pérez F.-J. (2018). A Framework to Design the Computational Load Distribution of Wireless Sensor Networks in Power Consumption Constrained Environments. Sensors.

[44] Braun T., Kovalenko M.V., Grillo D.E., Dupin G., Brunel L., Colliex C. (2010). Interface Tunneling in Metal-Insulator-Metal Junctions. J. Phys. Rev. Lett..

[45] Morin É., Maman M., Guizzetti R., Duda A. (2017). Comparison of the Device Lifetime in Wireless Networks for the Internet of Things. IEEE Access.

[46] Mansour M., Gamal A., Ahmed A.I., Said L.A., Elbaz A., Herencsar N., Soltan A. (2023). Internet of Things: A Comprehensive Overview on Protocols, Architectures, Technologies, Simulation Tools, and Future Directions. Energies.

[47] Augustin A., Yi J., Clausen T., Townsley W. (2016). A Study of LoRa: Long Range & Low Power Networks for the Internet of Things. Sensors.

[48] Dlodlo N., Kalezhi J., Dlodlo N., Kalezhi J. The internet of things in agriculture for sustainable rural development. Proceedings of the International Conference on Emerging Trends in Networks and Computer Communications (ETNCC).

[49] Lee J.S., Su Y.W., Shen C.C. A Comparative Study of Wireless Protocols: Bluetooth, UWB, ZigBee, and Wi-Fi. Proceedings of the IECON 2007—33rd Annual Conference of the IEEE Industrial Electronics Society.

[50] Jawad H.M., Nordin R., Gharghan S.K., Jawad A.M., Ismail M. (2017). Energy-Efficient Wireless Sensor Networks for Precision Agriculture: A Review. Sensors.

[51] Tang Y., Dananjayan S., Hou C., Guo Q., Luo S., He Y. (2021). A survey on the 5G network and its impact on agriculture: Challenges and opportunities. Comput. Electron. Agric..

[52] Heikkilä M., Suomalainen J., Saukko O., Kippola T., Lähetkangas K., Koskela P., Kalliovaara J., Haapala H., Pirttiniemi J., Yastrebova A. (2022). Unmanned Agricultural Tractors in Private Mobile Networks. Network.

[53] Foubert B., Mitton N. (2020). Long-Range Wireless Radio Technologies: A Survey. Future Internet.

[54] Hughes L., Wang X., Chen T. (2012). A Review of Protocol Implementations and Energy Efficient Cross-Layer Design for Wireless Body Area Networks. Sensors.

[55] Tomaszewski L., Kołakowski R. (2023). Mobile Services for Smart Agriculture and Forestry, Biodiversity Monitoring, and Water Management: Challenges for 5G/6G Networks. Telecom.

[56] Liao Y., Liu S., Hong X., Shi J., Cheng L. (2023). Integration of Communication and Navigation Technologies toward LEO-Enabled 6G Networks: A Survey. Space Sci. Technol..

[57] Banafaa M., Shayea I., Din J., Azmi M.H., Alashbi A., Daradkeh Y.I., Alhammadi A. (2023). 6G Mobile Communication Technology: Requirements, Targets, Applications, Challenges, Advantages, and Opportunities. Alex. Eng. J..

[58] Barua A., Bhadra G.P., Rasel M.S. A Universal Energy Harvesting System for Ultra-Low Power Management and IoT Applications. Proceedings of the 2021 5th International Conference on Electrical Engineering and Information Communication Technology (ICEEICT).

[59] Lhermet H., Condemine C., Plissonnier M., Salot R., Audebert P., Rosset M. (2008). Efficient Power Management Circuit: From Thermal Energy Harvesting to Above-IC Microbattery Energy Storage. IEEE J. Solid-State Circuits.

[60] Shirvanimoghaddam M., Shirvanimoghaddam K., Abolhasani M.M., Farhangi M., Barsari V.Z., Liu H., Dohler M., Naebe M., Kara G. (2019). Towards a Green and Self-Powered Internet of Things Using Piezoelectric Energy Harvesting. IEEE Access.

[61] Vallem V., Sargolzaeiaval Y., Ozturk M., Lai Y.C., Dickey M.D. (2021). Energy Harvesting and Storage with Soft and Stretchable Materials. Adv. Mater..

[62] LTC3108. https://www.analog.com/en/products/ltc3108.html.

[63] LTC3105. https://www.analog.com/en/products/ltc3105.html.

[64] Pimentel D., Musilek P. Power management with energy harvesting devices. Proceedings of the CCECE 2010.

[65] Kansal A., Hsu J., Zahedi S., Srivastava M.B. (2007). Power management in energy harvesting sensor networks. ACM Trans. Embed. Comput. Syst..

[66] Rahimi M., Shah H., Sukhatme G.S., Heideman J., Estrin D. Studying the feasibility of energy harvesting in a mobile sensor network. Proceedings of the 2003 IEEE International Conference on Robotics and Automation (Cat. No. 03CH37422).

[67] Abdin Z., Alim M.A., Saidur R., Islam M.R., Rashmi W., Mekhilef S., Wadi A. (2013). Solar energy harvesting with the application of nanotechnology. Renew. Sustain. Energy Rev..

[68] Wayu M. (2021). Manganese Oxide Carbon-Based Nanocomposite in Energy Storage Applications. Solids.

[69] Nkembi A.A., Simonazzi M., Santoro D., Cova P., Delmonte N. (2024). Comprehensive Review of Energy Storage Systems Characteristics and Models for Automotive Applications. Batteries.

[70] Hezekiah J.D.K., Ramya K.C., Radhakrishnan S.B.K., Kumarasamy V.M., Devendran M., Ramalingam A., Maheswar R. (2023). Review of Next-Generation Wireless Devices with Self-Energy Harvesting for Sustainability Improvement. Energies.

[71] Itani K., De Bernardinis A. (2023). Review on New-Generation Batteries Technologies: Trends and Future Directions. Energies.

[72] Johnson M., Healy M., van de Ven P., Hayes M.J., Nelson J., Newe T., Lewis E. A comparative review of wireless sensor network mote technologies. Proceedings of the SENSORS, 2009 IEEE.

[73] Pham N.N., Bloudicek R., Leuchter J., Rydlo S., Dong Q.H. (2024). Comparative Analysis of Energy Storage and Buffer Units for Electric Military Vehicle: Survey of Experimental Results. Batteries.

[74] Yaqoob L., Noor T., Iqbal N. (2022). An overview of metal-air batteries, current progress, and future perspectives. J. Energy Storage.

[75] Aneke M., Wang M. (2016). Energy storage technologies and real life applications—A state of the art review. Appl. Energy.

[76] Zhang C., Wei Y.L., Cao P.F., Lin M.C. (2017). Energy storage system: Current studies on batteries and power condition system. Renew. Sustain. Energy Rev..

[77] Taneja J., Jeong J., Culler D. Design, modeling, and capacity planning for micro-solar power sensor networks. Proceedings of the 7th International Conference on Information Processing in Sensor Networks.

[78] Yadav G.G., Wei X., Huang J., Turney D., Nyce M., Banerjee S. (2018). Accessing the second electron capacity of MnO2 by exploring complexation and intercalation reactions in energy dense alkaline batteries. Int. J. Hydrog. Energy.

[79] Akinyele D., Rayudu R. (2014). Review of energy storage technologies for sustainable power networks. Sustain. Energy Technol. Assess..

[80] Kaldellis J., Zafirakis D. (2007). Optimum energy storage techniques for the improvement of renewable energy sources-based electricity generation economic efficiency. Energy.

[81] Hannan M.A., Hoque M.M., Mohamed A., Ayob A. (2017). Review of Energy Storage Systems for Electric Vehicle Applications: Issues and Challenges. Renew. Sustain. Energy Rev..

[82] Upadhyaya A., Mahanta C. (2024). An Overview of Battery Based Electric Vehicle Technologies with Emphasis on Energy Sources, Their Configuration Topologies and Management Strategies. IEEE Trans. Intell. Transp. Syst..

[83] Beardsall J.C., Gould C.A., Al-Tai M. Energy Storage Systems: A Review of the Technology and Its Application in Power Systems. Proceedings of the 2015 50th International Universities Power Engineering Conference (UPEC).

[84] Abbas Q., Mirzaeian M., Hunt M.R.C., Hall P., Raza R. (2020). Current State and Future Prospects for Electrochemical Energy Storage and Conversion Systems. Energies.

[85] Hylla P., Trawinski T., Polnik B., Burlikowski W., Prostanski D. (2023). Overview of Hybrid Energy Storage Systems Combined with RES in Poland. Energies.

[86] Sahin M., Blaabjerg F., Sangwongwanich A. (2022). A Comprehensive Review on Supercapacitor Applications and Developments. Energies.

[87] Rudra S., Seo H.W., Sarker S., Kim D.M. (2024). Supercapatteries as Hybrid Electrochemical Energy Storage Devices: Current Status and Future Prospects. Molecules.

[88] Aftab J., Mehmood S., Ali A., Ahmad I., Bhopal M.F., Rehman M.Z.U., Shah M.Z.U., Shah A.U., Wang M., Khan M.F. (2023). Synergetic electrochemical performance of tungsten oxide/tungsten disulfide/MWCNTs for high-performance aqueous asymmetric supercapattery devices. J. Alloys Compd..

[89] Khan M.F., Marwat M.A., Abdullah, Shah S.S., Karim M.R.A., Aziz M.A., Din Z.U., Ali S., Adam K.M. (2023). Novel MoS2-sputtered NiCoMg MOFs for high-performance hybrid supercapacitor applications. Sep. Purif. Technol..

[90] Raja T.A., Vickraman P. (2022). Role of dual redox additives KI/VOSO4 in manganese ammonium phosphate at graphene quantum dots for supercapattery. Int. J. Energy Res..

[91] Shehzad W., Karim M.R.A., Iqbal M.Z., Shahzad N., Ali A. (2022). Sono-chemical assisted synthesis of carbon nanotubes-nickel phosphate nanocomposites with excellent energy density and cyclic stability for supercapattery applications. J. Energy Storage.

[92] Ghanem L.G., Taha M.M., Salama M., Allam N.K. (2022). Binder-free Mn-V-Sn oxyhydroxide decorated with metallic Sn as an earth-abundant supercapattery electrode for intensified energy storage. Sustain. Energy Fuels.

[93] Moradi M., Mousavi M., Pooriraj M., Babamoradi M., Hajati S. (2022). Enhanced pseudocapacitive performance of two-dimensional Zn-metal organic framework through a post-synthetic amine functionalization. Thin Solid Film..

[94] Iqbal M.Z., Khan J., Afzal A.M., Aftab S. (2021). Exploring the synergetic electrochemical performance of cobalt sulfide/cobalt phosphate composites for supercapattery devices with high-energy and rate capability. Electrochim. Acta.

[95] Wu H., Qiu Z. (2021). Fe_3_O_4_@N-porous carbon nano rice/rGO sheet as positive electrode material for a high performance supercapattery. J. Alloys Compd..

[96] Sharmila V., Packiaraj R., Nallamuthu N., Parthibavarman M. (2020). Fabrication of MWCNTs wrapped nickel manganese phosphate asymmetric capacitor as a supercapattery electrode for energy storage applications. Inorg. Chem. Commun..

[97] Liu G., Xie J., Sun Y., Zhang P., Li X., Zheng L., Hao L., Shanmin G. (2021). Constructing 3D honeycomb-like CoMn_2_O_4_ nanoarchitecture on nitrogen-doped graphene coating Ni foam as flexible battery-type electrodes for advanced supercapattery. Int. J. Hydrog. Energy.

[98] Murugan C., Subramani K., Subash R., Sathish M., Pandikumar A. (2020). High-performance high-voltage symmetric supercapattery based on a graphitic carbon nitride/bismuth vanadate nanocomposite. Energy Fuels.

[99] Yu F., Zhang C., Wang F., Gu Y., Zhang P., Waclawik E.R., Du A., Ostrikov K., Wang H. (2020). A zinc bromine “supercapattery” system combining triple functions of capacitive, pseudocapacitive and battery-type charge storage. Mater. Horiz..

[100] Nadeem F., Hussain S.M.S., Tiwari P.K., Goswami A.K., Ustun T.S. (2019). Comparative Review of Energy Storage Systems, Their Roles, and Impacts on Future Power Systems. IEEE Access.

[101] Neburchilov V., Zhang J. (2016). Metal-Air and Metal-Sulfur Batteries.

[102] Riaz A., Sarker M.R., Saad M.H.M., Mohamed R. (2021). Review on Comparison of Different Energy Storage Technologies Used in Micro-Energy Harvesting, WSNs, Low-Cost Microelectronic Devices: Challenges and Recommendations. Sensors.

[103] Wu B., Chen C., Danilov D.L., Eichel R.A., Notten P.H.L. (2023). All-Solid-State Thin Film Li-Ion Batteries: New Challenges, New Materials, and New Designs. Batteries.

[104] Kanamura K., Toriyama S., Shiraishi S., Takehara Z. (1995). Studies on Electrochemical Oxidation of Nonaqueous Electrolytes Using In Situ FTIR Spectroscopy: I. The Effect of Type of Electrode on On-Set Potential for Electrochemical Oxidation of Propylene Carbonate Containing 1.0 mol dm^−3^. J. Electrochem. Soc..

[105] Huang S., Chen Y., Liu Q. (2018). Electrochemical behavior and application of a silver electrode in a 1 M LiPF6 solution. J. Alloys Compd..

[106] Gu Y., Federici J.F. (2018). Fabrication of a Flexible Current Collector for Lithium Ion Batteries by Inkjet Printing. Batteries.

[107] Fredriksson W., Edström K. (2012). XPS study of duplex stainless steel as a possible current collector in a Li-ion battery. Electrochim. Acta.

[108] Myung S.-T., Sasaki Y., Sakurada S., Sun Y.-K., Yashiro H. (2009). Electrochemical behavior of current collectors for lithium batteries in non-aqueous alkyl carbonate solution and surface analysis by ToF-SIMS. Electrochim. Acta.

[109] Gao T., Qu Q., Zhu G., Shi Q., Qian F., Shao J., Zheng H. (2016). A self-supported carbon nanofiber paper/sulfur anode with high capacity and high-power for application in Li-ion batteries. Carbon.

[110] Adu-Manu K.S., Adam N., Tapparello C., Ayatollahi H., Heinzelman W. (2018). Energy-Harvesting Wireless Sensor Networks (EH-WSNs): A Review. ACM Trans. Sens. Netw..

[111] Akbari S. Energy harvesting for wireless sensor networks review. Proceedings of the 2014 Federated Conference on Computer Science and Information Systems (FedCSIS).

[112] Jiang X., Polastre J., Culler D. Perpetual environmentally powered sensor networks. Proceedings of the 4th International Symposium on Information Processing in Sensor Networks (IPSN’05).

[113] Peng S., Low C.P. Energy-neutral routing for energy-harvesting wireless sensor networks. Proceedings of the 2013 IEEE Wireless Communications and Networking Conference (WCNC).

[114] Peng S., Wang T., Low C.P. (2015). Energy-neutral clustering for energy-harvesting wireless sensors networks. Ad Hoc Netw..

[115] Kanoun O., Bradai S., Khriji S., Bouattour G., El Houssaini D., Ben Ammar M., Naifar S., Bouhamed A., Derbel F., Viehweger C. (2021). Energy-Aware System Design for Autonomous Wireless Sensor Nodes: A Comprehensive Review. Sensors.

[116] Al-Ezzi A.S., Ansari M.N.M. (2022). Photovoltaic Solar Cells: A Review. Appl. Syst. Innov..

[117] Kawamoto H. (2019). Electrostatic cleaning equipment for dust removal from soiled solar panels. J. Electrostat..

[118] Mahdi Elsiddig Haroun F., Mohamad Deros S.N., Ahmed Alkahtani A., Md Din N. (2022). Towards Self-Powered WSN: The Design of Ultra-Low-Power Wireless Sensor Transmission Unit Based on Indoor Solar Energy Harvester. Electronics.

[119] Dallaev R., Pisarenko T., Papež N., Holcman V. (2023). Overview of the Current State of Flexible Solar Panels and Photovoltaic Materials. Materials.

[120] Deshpande R.A. (2021). Advances in Solar Cell Technology: An Overview. J. Sci. Res..

[121] Roy S., Baruah M.S., Sahu S., Nayak B.B. (2021). Computational analysis on the thermal and mechanical properties of thin film solar cells. Mater. Today Proc..

[122] Zekry A., Shaker A., Salem M. (2018). Solar Cells and Arrays: Principles, Analysis, and Design.

[123] Rouway M., Boulahia Z., Chakhchaoui N., Fouzia F., El Hachemi Omari L., Cherkaoui O., Van Langenhove L. (2020). Mathematical and numerical modelling of soiling effects of photovoltaic solar panels on their electrical performance. IOP Conf. Ser. Mater. Sci. Eng..

[124] Yu P.Y., Cardona M. (2001). Fundamentals of Semiconductors: Physics and Materials Properties.

[125] He G., Zhou C., Li Z. (2011). Review of Self-Cleaning Method for Solar Cell Array. Procedia Eng..

[126] Lee T.D., Ebong A.U. (2017). A review of thin film solar cell technologies and challenges. Renew. Sustain. Energy Rev..

[127] Qarony W., Hossain M.I., Hossain M.K., Uddin M.J., Haque A., Saad A.R., Tsang Y.H. (2017). Efficient amorphous silicon solar cells: Characterization, optimization, and optical loss analysis. Results Phys..

[128] Almosni S., Delamarre A., Jehl Z., Suchet D., Cojocaru L., Giteau M., Behaghel B., Julian A., Ibrahim C., Tatry L. (2018). Material challenges for solar cells in the twenty-first century: Directions in emerging technologies. Sci. Technol. Adv. Mater..

[129] Gray J.L. (2011). The Physics of the Solar Cell. Handbook of Photovoltaic Science and Engineering.

[130] Shah D.K., KC D., Muddassir M., Akhtar M.S., Kim C.Y., Yang O.B. (2021). A simulation approach for investigating the performances of cadmium telluride solar cells using doping concentrations, carrier lifetimes, thickness of layers, and band gaps. Sol. Energy.

[131] Godt J., Scheidig F., Grosse-Siestrup C., Esche V., Brandenburg P., Reich A., Groneberg D.A. (2006). The toxicity of cadmium and resulting hazards for human health. J. Occup. Med. Toxicol..

[132] Ramanujam J., Singh U.P. (2017). Copper indium gallium selenide based solar cells—A review. Energy Environ. Sci..

[133] Mufti N., Amrillah T., Taufiq A., Diantoro M., Nur H. (2020). Review of CIGS-based solar cells manufacturing by structural engineering. Sol. Energy.

[134] Frenzel M., Mikolajczak C., Reuter M.A., Gutzmer J. (2017). Quantifying the relative availability of high-tech by-product metals—The cases of gallium, germanium and indium. Resour. Policy.

[135] Gul M., Kotak Y., Muneer T. (2016). Review on recent trend of solar photovoltaic technology. Energy Explor. Exploit..

[136] Papež N., Škvarenina L., Tofel P., Sobola D. Thermal stability of gallium arsenide solar cells. Proceedings of the Photonics, Devices, and Systems VII.

[137] Pouladi S., Asadirad M., Oh S.K., Shervin S., Chen J., Wang W., Manh C.N., Choi R., Kim J., Khatiwada D. (2019). Effects of grain boundaries on conversion efficiencies of single-crystal-like GaAs thin-film solar cells on flexible metal tapes. Sol. Energy Mater. Sol. Cells.

[138] Park S., Simon J., Schulte K.L., Ptak A.J., Wi J.S., Young D.L., Oh J. (2019). Germanium-on-Nothing for Epitaxial Liftoff of GaAs Solar Cells. Joule.

[139] Rahaman M.S., Rahman M.M., Mise N., Sikder M.T., Ichihara G., Uddin M.K., Kurasaki M., Ichihara S. (2021). Environmental arsenic exposure and its contribution to human diseases, toxicity mechanism and management. Environ. Pollut..

[140] Green M.A., Emery K., King D.L., Igari S., Warta W. (2002). Solar cell efficiency tables (version 19). Prog. Photovolt. Res. Appl..

[141] Green M.A., Emery K., King D.L., Igari S., Warta W. (2003). Solar cell efficiency tables (version 22). Prog. Photovolt. Res. Appl..

[142] Green M.A., Emery K., King D.L., Igari S., Warta W. (2005). Solar cell efficiency tables (version 26). Prog. Photovolt. Res. Appl..

[143] Green M.A., Emery K., King D.L., Igari S., Warta W. (2006). Solar cell efficiency tables (version 27). Prog. Photovolt. Res. Appl..

[144] Maziviero F.V., Melo D.M.A., Medeiros R.L.B.A., Oliveira Â.A.S., Macedo H.P., Braga R.M., Morgado E. (2024). Advancements and Prospects in Perovskite Solar Cells: From Hybrid to All-Inorganic Materials. Nanomaterials.

[145] Bett A.J., Schulze P.S.C., Winkler K.M., Kabakli Ö.S., Ketterer I., Mundt L.E., Reichmuth S.K., Siefer G., Cojocaru L., Tutsch L. (2020). Two-terminal Perovskite silicon tandem solar cells with a high-Bandgap Perovskite absorber enabling voltages over 1.8 V. Prog. Photovolt..

[146] Chen M., Ju M.G., Garces H.F., Carl A.D., Ono L.K., Hawash Z., Zhang Y., Shen T., Qi Y., Grimm R.L. (2019). Highly stable and efficient all-inorganic lead-free perovskite solar cells with native-oxide passivation. Nat. Commun..

[147] Hao F., Stoumpos K., Cao D.H., Chang R.P.H., Kanatzidis M. (2014). Lead-free solid-state organic-inorganic halide perovskite solar cells. Nat. Photonics.

[148] Bansode U., Naphade R., Game O., Agarkar S., Ogale S. (2015). Hybrid perovskite films by a new variant of pulsed excimer laser deposition: A roomerature dry process. J. Phys. Chem. C.

[149] Shao S., Liu J., Portale G., Fang H.H., Blake G.R., ten Brink G.H., Koster L.J.A., Loi M.A. (2018). Highly Reproducible Sn-Based Hybrid Perovskite Solar Cells with 9% Efficiency. Adv. Energy Mater..

[150] Jokar E., Chien C., Tsai C., Fathi A., Diau E.W. (2018). Robust Tin-Based Perovskite Solar Cells with Hybrid Organic Cations to Attain Efficiency Approaching 10%. Adv. Mater..

[151] Zuo F., Williams S.T., Liang P.W., Chueh C.C., Liao C.Y., Jen A.K.Y. (2014). Binary-Metal Perovskites Toward High-Performance Planar-Heterojunction Hybrid Solar Cells. Adv. Mater..

[152] Chung I., Lee B., He J., Chang R.P.H., Kanatzidis M.G. (2012). All-solid-state dye-sensitized solar cells with high efficiency. Nature.

[153] Lee S., Kang D. (2017). Highly Efficient and Stable Sn-rich Perovskite Solar Cells by Introducing Bromine Highly Efficient and Stable Sn-rich Perovskite Solar Cells by Introducing Bromine. ACS Appl. Mater. Interfaces.

[154] Mohanty I., Mangal S., Udai P.S. (2021). Performance optimization of lead free-MASnI3/CIGS heterojunction solar cell with 28.7% efficiency: A numerical approach. Opt. Mater..

[155] Yang Z., Rajagopal A., Chueh C., Jo S.B., Liu B., Zhao T., Jen A.K. (2016). Stable Low-Bandgap Pb–Sn Binary Perovskites for Tandem Solar Cells. Adv. Mater..

[156] Eperon G.E., Leijtens T., Bush K.A., Prasanna R., Green T., Wang J., McMeekin D.P., Volonakis G., Milot R.L., May R. (2016). Perovskite-perovskite tandem photovoltaics with optimized band gaps. Science.

[157] Liao W., Zhao D., Yu Y., Shrestha N., Ghimire K., Grice C.R., Wang C., Xiao Y., Cimaroli A.J., Ellingson R.J. (2016). Fabrication of Efficient Low-Bandgap Perovskite Solar Cells by Combining Formamidinium Tin Iodide with Methyl ammonium Lead Iodide. J. Am. Chem. Soc..

[158] Wang Z.K., Li M., Yang Y.G., Hu Y., Ma H., Gao X.Y., Liao L.S. (2016). High Efficiency Pb–In Binary Metal Perovskite Solar Cells. Adv. Mater..

[159] Fievez M., Rana P.J.S., Koh T.M., Manceau M., Lew J.H., Jamaludin N.F., Ghosh B., Bruno A., Cros S., Berson S. (2021). Slot-die coated methyl ammonium-free perovskite solar cells with 18% efficiency. Sol. Energy Mater. Sol. Cells.

[160] Ramirez I., Zhang J., Ducati C., Grovenor C., Johnston M.B., Ginger D.S., Nicholas J., Snaith H.J. (2016). Environmental Science. Energy Environ. Sci..

[161] Zheng X., Hou Y., Bao C., Yin J., Yuan F., Huang Z., Song K., Liu J., Troughton J., Gasparini N. (2020). Managing grains and interfaces via ligand anchoring enables 22.3%-efficiency inverted perovskite solar cells. Nat. Energy.

[162] Cacovich S., Vidon G., Degani M., Legrand M., Gouda L., Puel J., Vaynzof Y., Guillemoles J., Ory D., Grancini G. (2022). Imaging and quantifying non-radiative losses at 23% efficient inverted perovskite solar cells interfaces. Nat. Commun..

[163] Degani M., An Q., Albaladejo-Siguan M., Hofstetter Y.J., Cho C., Paulus F., Grancini G., Vaynzof Y. (2021). 23.7% Efficient inverted perovskite solar cells by dual interfacial modification. Sci. Adv..

[164] Jeong M.J., Yeom K.M., Kim S.J., Jung E.H., Noh J.H. (2021). Spontaneous interface engineering for dopant-free poly(3-hexylthiophene) perovskite solar cells with efficiency over 24%. Energy Environ. Sci..

[165] Jeong M., Choi I.W., Go E.M., Cho Y., Kim M., Lee B., Jeong S., Jo Y., Choi H.W., Lee J. (2020). Stable perovskite solar cells with efficiency exceeding 24.8% and 0.3-V voltage loss. Science.

[166] Feng X., Guo Q., Xiu J., Ying Z., Ng K.W., Huang L., Wang S., Pan H., Tang Z., He Z. (2021). Close-loop recycling of perovskite solar cells through dissolution-recrystallization of perovskite by butylamine. Cell Rep. Phys. Sci..

[167] Min H., Lee D.Y., Kim J., Kim G., Lee K.S., Kim J., Paik M.J., Kim Y.K., Kim K.S., Kim M.G. (2021). Perovskite solar cells with atomically coherent interlayers on SnO_2_ electrodes. Nature.

[168] Shriwastava S., Tripathi C.C. (2019). Metal–Insulator–Metal Diodes: A Potential High Frequency Rectifier for Rectenna Application. J. Electron. Mater..

[169] Luo Y., Pu L., Wang G., Zhao Y. (2019). RF Energy Harvesting Wireless Communications: RF Environment, Device Hardware and Practical Issues. Sensors.

[170] Piñuela M., Mitcheson P.D., Lucyszyn S. (2013). Ambient RF energy harvesting in urban and semi-urban environments. IEEE Trans. Microw. Theory Tech..

[171] Roy S., Tiang J.J., Roslee M.B., Ahmed M.T., Kouzani A.Z., Mahmud M.A.P. (2021). Quad-Band Rectenna for Ambient Radio Frequency (RF) Energy Harvesting. Sensors.

[172] Lee W., Choi S., Kim H., Hwang S., Jeon S., Yoon Y.-K. (2021). Metamaterial-Integrated High-Gain Rectenna for RF Sensing and Energy Harvesting Applications. Sensors.

[173] Gasulla M., Ripoll-Vercellone E., Reverter F. (2019). A Compact Thévenin Model for a Rectenna and Its Application to an RF Harvester with MPPT. Sensors.

[174] Wang Y., Lu N., Sun H., Ren R. (2023). A Dual-Polarized Omnidirectional Rectenna Array for RF Energy Harvesting. Micromachines.

[175] De Donno D., Catarinucci L., Tarricone L. (2014). RAMSES: RFID Augmented Module for Smart Environmental Sensing. IEEE Trans. Instrum. Meas..

[176] Singh N., Kumar S., Kanaujia B.K., Beg M.T., Mainuddin, Kumar, S (2020). A compact broadband GFET based rectenna for RF energy harvesting applications. Microsyst. Technol..

[177] Koohestani M., Tissier J., Latrach M. (2020). A miniaturized printed rectenna for wireless RF energy harvesting around 2.45 GHz. AEU Int. J. Electron. Commun..

[178] Chi Y.-J., Lin C.-H., Chiu C.-W. (2020). Design and modeling of a wearable textile rectenna array implemented on Cordura fabric for batteryless applications. J. Electromagn. Waves Appl..

[179] Potti D., Mohammed G.N.A., Savarimuthu K., Narendhiran S., Rajamanickam G. (2020). An ultra-wideband rectenna using optically transparent Vivaldi antenna for radio frequency energy harvesting. Int. J. RF Microw. Comput. Eng..

[180] Pandey R., Shankhwar A.K., Singh A.A. (2021). An Improved Conversion efficiency of 1.975 to 4.744 GHz Rectenna for Wireless Sensor Applications. Prog. Electromagn. Res. C.

[181] Fakharian M.M. (2020). A Wideband Rectenna Using High Gain Fractal Planar Monopole Antenna Array for RF Energy Scavenging. Int. J. Antennas Propag..

[182] Eltresy N.A., Dardeer O.M., Al-Habal A., ElHariri E., Abotaleb A.M., Elsheakh D.N., Khattab A., Taie S.A., Mostafa H., Elsadek H.A. (2020). Smart Home IoT System by Using RF Energy Harvesting. J. Sens..

[183] Benhamou A., Tellache M., Hebib S., Mahfoudi H. (2020). A wide input power range rectenna for energy harvesting and wireless power transfer applications. Int. J. RF Microw. Comput. Eng..

[184] He Z., Liu C. (2020). A Compact High-Efficiency Broadband Rectifier with a Wide Dynamic Range of Input Power for Energy Harvesting. IEEE Microw. Wirel. Compon. Lett..

[185] Reed R., Pour F.L., Ha D.S. An Efficient 2.4 GHz Differential Rectenna for Radio Frequency Energy Harvesting. Proceedings of the 2020 IEEE 63rd International Midwest Symposium on Circuits and Systems (MWSCAS).

[186] Hu Y.-Y., Sun S., Su H.-J., Yang S., Hu J. (2021). Dual-Beam Rectenna Based on a Short Series-Coupled Patch Array. IEEE Trans. Antennas Propag..

[187] Chandrasekaran K.T., Agarwal K., Alphones A., Mittra R., Karim M.F. (2020). Compact Dual-Band Metamaterial-Based High-Efficiency Rectenna: An Application for Ambient Electromagnetic Energy Harvesting. IEEE Antennas Propag. Mag..

[188] Almoneef T.S. (2020). Design of a Rectenna Array without a Matching Network. IEEE Access.

[189] Lin W., Ziolkowski R.W. (2020). Electrically Small, Single-Substrate Huygens Dipole Rectenna for Ultra-compact Wireless Power Transfer Applications. IEEE Trans. Antennas Propag..

[190] Wagih M., Hilton G.S., Weddell A.S., Beeby S. (2020). Broadband Millimeter-Wave Textile-Based Flexible Rectenna for Wearable Energy Harvesting. IEEE Trans. Microw. Theory Tech..

[191] Wagih M., Hillier N., Yong S., Weddell A.S., Beeby S. (2021). RF-Powered Wearable Energy Harvesting and Storage Module Based on E-Textile Coplanar Waveguide Rectenna and Supercapacitor. IEEE Open J. Antennas Propag..

[192] Wagih M., Hilton G.S., Weddell A.S., Beeby S. (2021). Dual-Band Dual-Mode Textile Antenna/Rectenna for Simultaneous Wireless Information and Power Transfer (SWIPT). IEEE Trans. Antennas Propag..

[193] Citroni R., Di Paolo F., Livreri P. (2019). Evaluation of an optical energy harvester for SHM application. Int. J. Electron. Commun. (AEÜ).

[194] Citroni R., Di Paolo F., Livreri P. (2019). A Novel Energy Harvester for Powering Small UAVs: Performance Analysis, Model Validation and Flight Results. Sensors.

[195] Citroni R., Leggieri A., Passi D., Di Paolo F., Di Carlo A. (2017). Nano Energy Harvesting with Plasmonic Nano-Antennas: A review of MID-IR Rectenna and Application. Adv. Electromagn..

[196] Citroni R., Passi D., Leggieri A., Di Paolo F., Di Carlo A. The next generation: Miniaturized objects, self-powered using nanostructures to harvest ambient energy. Proceedings of the 18th Italian National Conference on Photonic Technologies (Fotonica 2016).

[197] Byrness S.J., Blanchard R., Capasso F. (2014). Harvesting renewable energy from Earth’s mid-infrared emissions. Proc. Natl. Acad. Sci. USA.

[198] Donchev E., Pang J.S., Gammon P.M., Centeno A., Xie F., Petrov P.K., Breeze J.D., Ryan M.P., Riley D.J., Alford N.M. (2014). The rectenna device: From theory to practice (a review). MRS Energy Sustain. Rev. J..

[199] Gadalla M.N., Abdel-Rahman M., Shamim A. (2014). Design, optimization and fabrication of a 28.3 THz nano-rectenna for infrared detection and rectification. Sci. Rep..

[200] Davids P.S., Jarecki R.L., Starbuck A., Burckel D.B., Kadlec E.A., Ribaudo T., Shaner E.A., Peters D.W. (2015). Infrared rectification in a nanoantenna-coupled metal-oxide-semiconductor tunnel diode. Nat. Nanotechnol..

[201] Belkadi A., Weerakkody A., Moddel G. (2021). Demonstration of resonant tunneling effects in metal-double-insulator-metal (MI^2^M) diodes. Nat. Commun..

[202] Hamied F.M.A., Mahmoud K.R., Hussein M., Obayya S.A.A. (2022). Design and analysis of a nano-rectenna based on multi-insulator tunnel barrier for solar energy harvesting. Opt. Quant. Electron..

[203] Moddel G., Grover S. (2013). Rectenna Solar Cells.

[204] Chien Chiu F. (2014). A Review on Conduction Mechanisms in Dielectric Films. Adv. Mater. Sci. Eng..

[205] Khan A.A., Jayaswal G., Gahaffar F.A., Shamim A. (2017). Metal-insulator-metal diodes with sub-nanometre surface roughness for energy-harvesting applications. Microelectron. Eng..

[206] Periasamy P., Gathers H.L., Abdulagatov A.I., Ndione P.F., Berry J.J., Ginley D.S., George S.M., Parilla P.A., O’Hayre R.P. (2013). Metal–Insulator–Metal Diodes: Role of the Insulator Layer on the Rectification Performance. Adv. Mater..

[207] Citroni R., Di Paolo F., Di Carlo A. (2018). Replacing Noble Metals with Alternative Metals in MID-IR Frequency: A Theoretical Approach. AIP Conf. Proc..

[208] Rawal Y., Ganguly S., Baghini M.S. (2012). Fabrication and Characterization of New Ti-TiO_2_-Al and Ti-TiO_2_-Pt Tunnel Diodes. Act. Passiv. Electron. Compon..

[209] Periasamy P., Berry J.J., Dameron A.A., Bergeson J.D., Ginley D.S., O’Hayre R.P., Parilla P.A. (2011). Fabrication and Characterization of MIM Diodes Based on Nb/Nb_2_O_5_ via a Rapid Screening Technique. Adv. Mater..

[210] Gadalla M.N., Shamim A. 28.3 THz Bowtie Antenna Integrated Rectifier for Infrared Energy Harvesting. Proceedings of the 2014 44th European Microwave Conference.

[211] Grover S., Dmitriyeva O., Estes M.J., Moddel G. (2010). Traveling-wave metal/insulator/metal diodes for improved infrared bandwidth and efficiency of antenna-coupled rectifiers. IEEE Trans. Nanotechn..

[212] Jin J., Wang L., Zheng Z., Zhang J., Hu X., Lu J.R., Etor D., Pearson C., Song A., Wood D. (2019). Metal-insulator-metal diodes based on alkyltrichlorosilane self-assembled monolayers. AIP Adv..

[213] China M.L., Periasamy P., O’Regan T.P., Amani M., Tan C., O’Hayre R.P., Berry J.J., Osgood R.M., Parilla P.A., Ginley D.S. (2013). Planar Metal-Insulator-Metal Diodes Based on the Nb/Nb_2_O_5_/X Material System. J. Vac. Sci. Technol..

[214] Lee J.H., Lin Y.C., Chen B.H., Tsai C.Y. New metal-insulator-metal capacitor based on SrTiO_3_/Al_2_O_3_/SrTiO_3_ laminate dielectric. Proceedings of the 2010 10th IEEE International Conference on Solid-State and Integrated Circuit Technology.

[215] Abdel-Rahman M., Syaryadhi M., Debbar N. (2013). Fabrication and characterization of high sensitivity copper-copper oxide-copper (Cu-CuO-Cu) metal-insulator-metal tunnel junctions. Electron. Lett..

[216] Alshehri A.H., Mistry K., Nguyen V.H., Ibrahim K.H., Muñoz-Rojas D., Yavuz M., Musselman K.P. (2018). Quantum-Tunneling Metal-Insulator-Metal Diodes Made by Rapid Atmospheric Pressure Chemical Vapor Deposition. Adv. Funct. Mater..

[217] Inac M., Shafique A., Ozcan M., Gurbuz Y. (2015). Model, design, and fabrication of antenna coupled metal-insulator-metal diodes for IR sensing. Proc. SPIE.

[218] Bhatt K., Shriwastava S., Kumar S., Tripathi S., Tripathi C.C. (2017). Chapter Terahertz Detectors (THzDs): Bridging the Gap for Energy Harvesting. Terahertz Spectroscopy—A Cutting Edge Technology.

[219] Matsuura D., Shimizu M., Yugami H. (2019). High-current density and high asymmetry MIIM diode based on oxygen-nonstoichiometry controlled homointerface structure for optical rectenna. Sci. Rep..

[220] Citroni R., Di Paolo F., Livreri P. (2022). Progress in THz Rectifier Technology: Research and Perspectives. Nanomaterials.

[221] Grover S., Moddel G. (2012). Engineering the current–voltage characteristics of metal–insulator–metal diodes using double-insulator tunnel barriers. Solid-State Electron..

[222] Aydinoglu F., Alhazmi M., Cui B., Ramahi O.M., Irannejad M., Brzezinski A., Yavuz M. (2014). Higher Performance Metal-Insulator-Metal Diodes using Multiple Insulator Layers. Austin. J. Nanomed. Nanotechnol..

[223] Weerakkody A.D., Sedghi N., Mitrovic I.Z., van Zalinge H., Nemr Noureddine I., Hall S., Wrench J.S., Chalker P.R., Phillips L.J., Treharne R. (2015). Enhanced low voltage nonlinearity in resonant tunneling metal-insulator-insulator-metal nanostructures. Microelectron. Eng..

[224] Herner S.B., Weerakkody A.D., Belkadi A., Moddel G. (2017). High performance MIIM diode based on cobalt oxide/titanium oxide. Appl. Phys. Lett..

[225] Elsharabasy A.Y., Alshehri A.H., Bakr M.H., Deen M.J., Musselman K.P., Yavuz M. (2019). Near zero-bias MIIM diode based on TiO2/ZnO for energy harvesting applications. AIP Adv..

[226] Maraghechi P., Foroughi-Abari A., Cadien K., Elezzabi A.Y. (2012). Observation of resonant tunneling phenomenon in metal-insulator-insulator- insulator-metal electron tunnel devices. Appl. Phys. Lett..

[227] Alisson B.J. (2001). Metal–Insulator–Metal Diodes for Solar Energy Conversion. Ph.D. Thesis.

[228] Maraghechi P., Foroughi-Abari A., Cadien K., Elezzabi A.Y. (2011). Enhanced rectifying response from metal-insulator-insulator-metal junctions. Appl. Phys. Lett..

[229] Alimardani N., Conley J.F. (2013). Step tunneling enhanced asymmetry in asymmetric electrode metal-insulator-insulator-metal tunnel diodes. Appl. Phys. Lett..

[230] Ajayi O.A. (2014). DC and RF Characterization of High Frequency ALD Enhanced Nanostructured Metal-Insulator-Metal Diodes. Ph.D. Thesis.

[231] Elsharabasy A.Y., Bakr M.H., Deen M.J. (2021). Towards an optimal MIIM diode for rectennas at 10.6 μm. Results Mater..

[232] Singha A., Ratnaduraib R., Kumara R., Krishnana S., Emirovc Y., Bhansali S. (2015). Fabrication and current–voltage characteristics of NiOx/ZnO based MIIM tunnel diode. Appl. Surf. Sci..

[233] Stearns J., Moddel G. (2021). Simulation of Z-Shaped Graphene Geometric Diodes Using Particle-in-Cell Monte Carlo Method in the Quasi-Ballistic Regime. Nanomaterials.

[234] Joshi S., Zhu Z., Grover S., Moddel G. Infrared optical response of geometric diode rectenna solar cells. Proceedings of the 2012 38th IEEE Photovoltaic Specialists Conference.

[235] Zhu Z., Grover S., Krueger K., Moddel G. Optical rectenna solar cells using graphene geometric diodes. Proceedings of the 2011 37th IEEE Photovoltaic Specialists Conference.

[236] Zhu Z., Joshi S., Grover S., Moddel G. (2013). Graphene geometric diodes for terahertz rectennas. J. Phys. D Appl. Phys..

[237] Wang H., Jayaswal G., Deokar G., Stearns J., Costa P.M.F.J., Moddel G., Shamim A. (2021). CVD-Grown Monolayer Graphene-Based Geometric Diode for THz Rectennas. Nanomaterials.

[238] Citroni R., D’Arrigo G., Livreri P. A mid-IR Plasmonic Graphene Nanorectenna-based Energy Harvester to Power IoT Sensors. Proceedings of the 2022 11th International Conference on Renewable Energy Research and Application (ICRERA).

[239] Citroni R., Livreri P. A Novel Shape of Bowtie Antenna Arranged in a Linear Array for Energy Harvesting in MID-IR Band. Proceedings of the 2023 12th International Conference on Renewable Energy Research and Applications (ICRERA).

[240] Biagioni P., Huang J.S., Hecht B. (2012). Nanoantennas for visible and infrared radiation. Rep. Prog. Phys..

[241] Maksymov I.S., Staude I., Miroshnichenko A.E., Kivshar Y.S. (2012). Optical Yagi-Uda nanoantennas. Nanophotonics.

[242] Di Garbo C., Livreri P., Vitale G. Optimal matching between optical rectennas and harvester circuits. Proceedings of the 2017 IEEE International Conference on Environment and Electrical Engineering and 2017 IEEE Industrial and Commercial Power Systems Europe (EEEIC/I&CPS Europe).

[243] Di Garbo C., Livreri P., Vitale G. (2016). Solar Nanoantennas energy based characterization. Renew. Energy Power Qual. J..

[244] Shuvo M.M.H. (2022). Edge AI: Leveraging the Full Potential of Deep Learning. Recent Innovations in Artificial Intelligence and Smart Applications, Studies in Computational Intelligence.

[245] Wahba M.A., Ashour A.S., Ghannam R. (2020). Prediction of Harvestable Energy for Self-Powered Wearable Healthcare Devices: Filling a Gap. IEEE Access.

[246] Kwan J.C., Chaulk J.M., Fapojuwo A.O. (2020). A Coordinated Ambient/Dedicated Radio Frequency Energy Harvesting Scheme Using Machine Learning. IEEE Sens. J..

[247] Hussein D., Bhat G., Doppa J.R. Adaptive Energy Management for Self-Sustainable Wearables in Mobile Health. Proceedings of the AAAI.

[248] Akinaga H. (2020). Recent advances and future prospects in energy harvesting technologies. Jpn. J. Appl. Phys..

[249] Ye Y., Azmat F., Adenopo I., Chen Y., Shi R. (2021). RF energy modelling using machine learning for energy harvesting communications systems. Int. J. Commun. Syst..

[250] Politi B., Foucaran A., Camara N. (2022). Low-Cost Sensors for Indoor PV Energy Harvesting Estimation Based on Machine Learning. Energies.

[251] Park Y., Cho K., Kim S. (2022). Performance Prediction of Hybrid Energy Harvesting Devices Using Machine Learning. ACS Appl. Mater. Interfaces.

[252] Zhu J., Cho M., Li Y., He T., Ahn J., Park J., Ren T.L., Lee C., Park I. (2021). Machine learning-enabled textile-based graphene gas sensing with energy harvesting-assisted IoT application. Nano Energy.

[253] Hamdi R., Chen M., Said A.B., Qaraqe M., Poor H.V. (2022). Federated Learning over Energy Harvesting Wireless Networks. IEEE Internet Things J..

[254] Guler B., Yener A. Energy-Harvesting Distributed Machine Learning. Proceedings of the 2021 IEEE International Symposium on Information Theory.

[255] Pervez I., Antoniadis C., Massoud Y. (2022). A Reduced Search Space Exploration Metaheuristic Algorithm for MPPT. IEEE Access.

[256] Peng Y., Wang Y., Liu Y., Gao K., Yin T., Yu H. (2022). Few-shot learning based multi-weather-condition impedance identification for MPPT-controlled PV converters. IET Renew. Power Gener..

[257] Singh Y., Pal N. (2021). Reinforcement learning with fuzzified reward approach for MPPT control of PV systems. Sustain. Energy Technol. Assess..

[258] Zafar M.H., Khan N.M., Mansoor M., Khan U.A. (2022). Towards green energy for sustainable development: Machine learning based MPPT approach for thermoelectric generator. J. Clean. Prod..

[259] Zhu W., Deng Y., Wang Y., Shen S., Gulfam R. (2016). High-performance photovoltaic-thermoelectric hybrid power generation system with optimized thermal management. Energy.

[260] Kraemer D., Hu L., Muto A., Chen X., Chen G., Chiesa M. (2008). Photovoltaic-thermoelectric hybrid systems: A general optimization methodology. Appl. Phys. Lett..

[261] Park K.-T., Shin S.M., Tazebay A.S., Um H.D., Jung J.Y., Jee S.W., Oh M.W., Park S.D., Yoo B., Yu C. (2013). Lossless hybridization between photovoltaic and thermoelectric devices. Sci. Rep..

[262] Li Y., Liu Y., Liu X., Wang X., Li Q. (2019). An energy extraction enhanced interface circuit for piezoelectric and thermoelectric energy harvesting. IEICE Electron. Express.

[263] Veloo S.G., Tiang J.J., Muhammad S., Wong S.K. (2023). A Hybrid Solar-RF Energy Harvesting System Based on an EM4325-Embedded RFID Tag. Electronics.

[264] Zhang Z., He T., Zhao J., Liu G., Wang Z.L., Zhang C. (2021). Tribo-thermoelectric and tribovoltaic coupling effect atmetal-semiconductor interface. Mater. Today Phys..

